# Supplementary material for: Combining accurate tumor genome simulation with crowdsourcing to benchmark somatic structural variant detection
Source: Genome Biol. 2018 Nov 6;19:188. doi: 10.1186/s13059-018-1539-5 (PMC6219177; doi:10.1186/s13059-018-1539-5)
Supplement: Supplementary file 1 — Figures S1-S26. (PDF 3614 kb) [file 13059_2018_1539_MOESM1_ESM.pdf]

Combining accurate tumour genome simulation with crowd-sourcing to benchmark somatic structural variant detection

Anna Y. Lee[1,10], Adam D. Ewing[2,3,10], Kyle Ellrott[2,10], Yin Hu[4], Kathleen E. Houlahan[1], J. Christopher Bare[4], Shadrielle Melijah G. Espiritu[1], Vincent Huang[1], Kristen Dang[4], Zechen Chong[5], Cristian Caloian[1], Takafumi N. Yamaguchi[1], ICGC-TCGA DREAM Somatic Mutation Calling Challenge Participants, Michael R. Kellen[4], Ken Chen[5], Thea C. Norman[4], Stephen H. Friend[4], Justin Guinney[4], Gustavo Stolovitzky[6], David Haussler[2], Adam A. Margolin[4,7,11], Joshua M. Stuart[2,11], Paul C. Boutros[1,8,9,11]

1 Informatics and Biocomputing Program; Ontario Institute for Cancer Research; Toronto, Ontario, Canada
2 Department of Biomolecular Engineering; University of California, Santa Cruz; Santa Cruz, CA, USA
3 Mater Research Institute; University of Queensland; Woolloongabba, QLD, Australia
4 Sage Bionetworks; Seattle, WA, USA
5 Department of Bioinformatics and Computational Biology, The University of Texas MD Anderson Cancer Center, Houston, TX, USA
6 IBM Computational Biology Center; T.J.Watson Research Center; Yorktown Heights, NY, USA
7 Computational Biology Program; Oregon Health & Science University; Portland, OR, USA
8 Department of Medical Biophysics; University of Toronto; Toronto, Ontario, Canada
9 Department of Pharmacology & Toxicology; University of Toronto; Toronto, Ontario, Canada
10 These authors contributed equally
11 Corresponding authors

Figures S1-S26

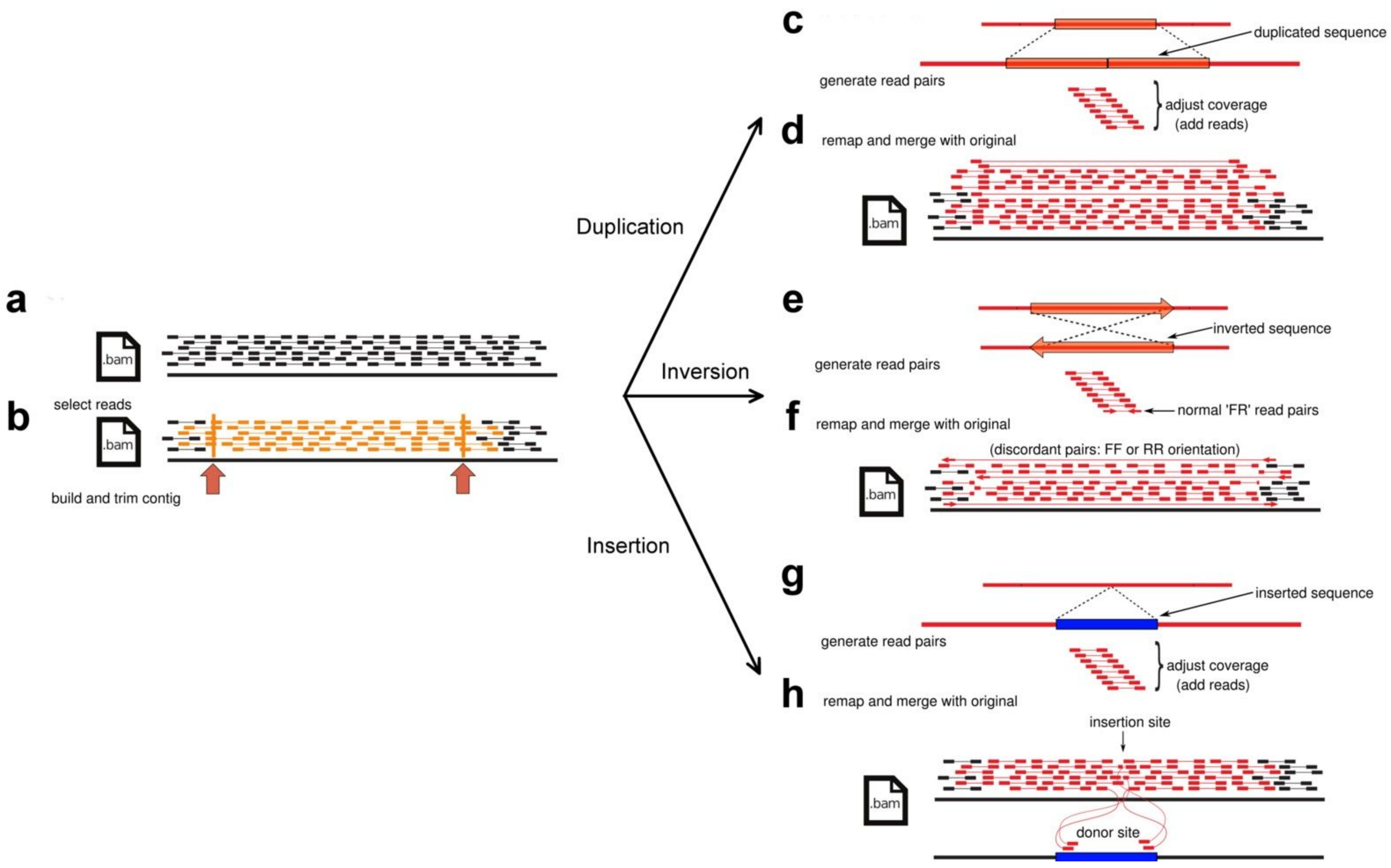

a
.bam
b
select reads
.bam
build and trim contig
Duplication
Inversion
Insertion
c
duplicated sequence
generate read pairs
adjust coverage
(add reads)
d
remap and merge with original
.bam
e
inverted sequence
generate read pairs
normal 'FR' read pairs
f
remap and merge with original
(discordant pairs: FF or RR orientation)
.bam
g
inserted sequence
generate read pairs
adjust coverage
(add reads)
h
remap and merge with original
insertion site
.bam
donor site

Figure S1 | BAMSurgeon process for creating structural variants.

Starting with an original BAM (**a**), a region (**b**) is selected where a structural variant (SV) is desired. **c,e,g** Contigs are assembled from reads in the selected region, and the contig is rearranged according to the type of SV desired. Read coverage is generated over the altered contig using wgsim to match the number of reads per base in the original BAM. **d,f,h** Generated reads will have different properties when mapped back to the reference genome, depending on the type of variant. Duplication: **c** The contig is rearranged by duplicating a segment of the contig (orange box). The number of duplications of the contig segment is a user-definable parameter. Since the duplication contig is longer than the original, more reads will be required to achieve the desired coverage. **d** Generated read pairs include discordant pairs and clipped reads spanning the junction between duplicated segments relative to the reference genome. Inversion: **e** The contig is rearranged by inverting a segment of the contig. Inversions by themselves do not alter read coverage except very locally over the breakpoints. **f** Whereas normal Illumina read pairs have a forward-reverse (FR) orientation relative to the reference genome, discordant reads mapping between the inverted segment and non-inverted sequence will display forward-forward (FF) or reverse-reverse (RR) orientations as shown. Insertion: **g** A user-defined inserted sequence is added to the midpoint of the contig. Additional reads are generated over the inserted sequence to achieve the desired coverage. **h** Generated read pairs include clipped reads overlapping the insertion site, and discordant reads linking the donor site (blue box) to the insertion point in cases where the donor sequence exists in the reference genome.

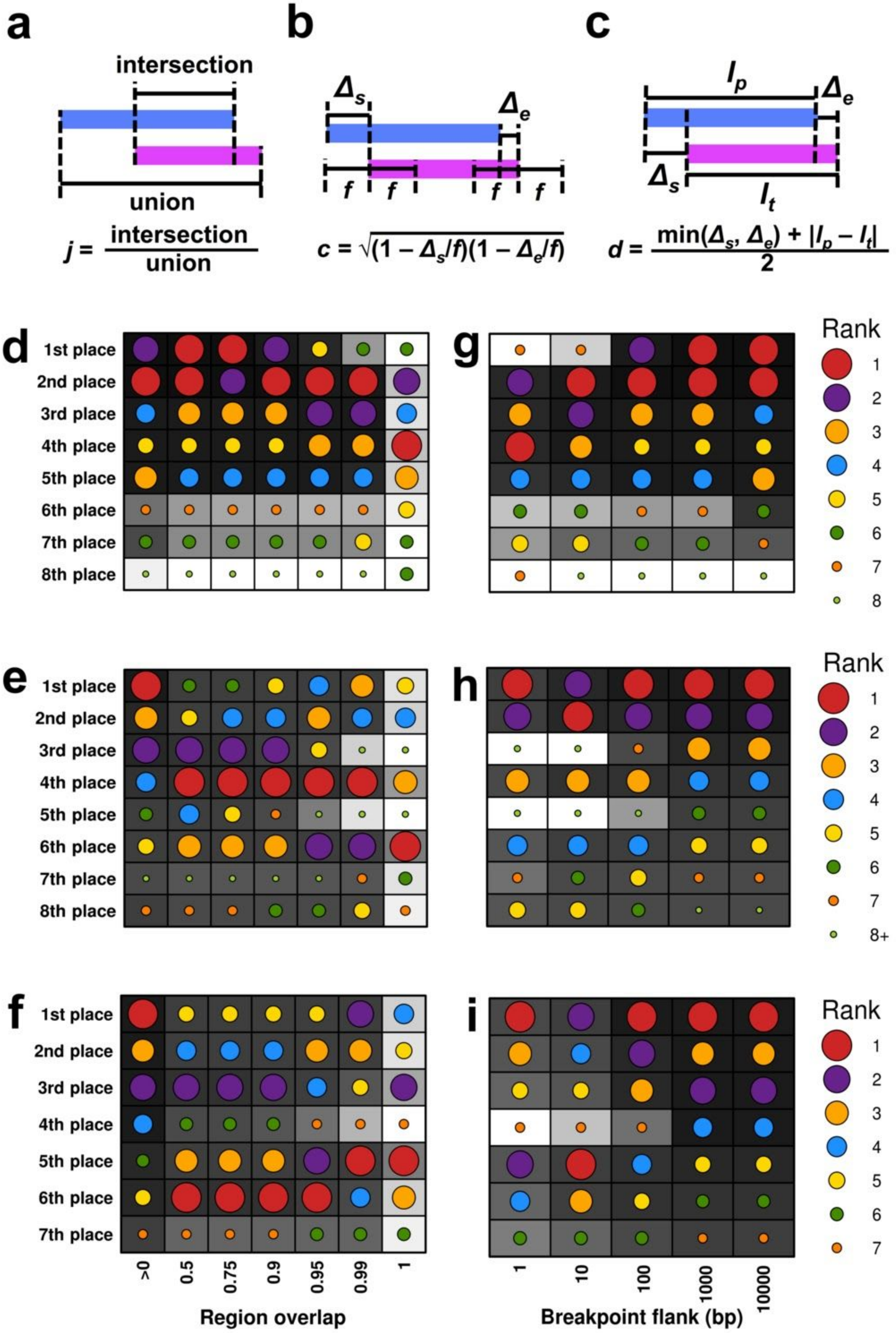

a
intersection
union
$j = \frac{\text{intersection}}{\text{union}}$
b
Δ_s
Δ_e
f
$c = \sqrt{(1 - \Delta_s/f)(1 - \Delta_e/f)}$
c
l_p
Δ_e
Δ_s
l_t
$d = \frac{\min(\Delta_s, \Delta_e) + |l_p - l_t|}{2}$
d
e
f
g
h
i
1st place
2nd place
3rd place
4th place
5th place
6th place
7th place
8th place
Rank
1
2
3
4
5
6
7
8
8+
>0
0.5
0.75
0.9
0.95
0.99
1
Region overlap
10
100
1000
10000
Breakpoint flank (bp)
0
0.25
0.5
0.75

Figure S2 | Caller scoring schemes.

Each scheme involves the comparison of a called SV (blue) to a known SV (pink). **a** Using the region overlap scheme, the degree of overlap in SV regions is measured with the Jaccard coefficient (*j*), with values ranging from 0 to 1 indicating no overlap and exact overlap, respectively. If $j \geq j_{min}$ where j_{min} is a selected threshold, the called SV is considered a true positive. **b** Using the breakpoint closeness scheme, the called SV is considered a true positive if its breakpoints fall within *f* bp of the breakpoints of the known SV, where *f* is a selected flank amount. Furthermore, we quantified closeness with the equation shown. **c** The breakpoint-length distance quantifies the distance between two SVs. It is defined as the average of (i) the minimum breakpoint distance and (ii) the difference in SV region length. Team performances on (**d**) IS1, (**e**) IS2 and (**f**) IS3, from scoring with the region overlap scheme at different overlap thresholds, are illustrated in *F*-score heatmaps. Teams are listed in their leaderboard order at the end of the sub-challenge (rows). For teams with multiple submissions, the greatest resulting *F*-score is shown. Darker shades indicate greater *F*-scores, and dots indicate team ranks by *F*-score. Team performances on (**g**) IS1, (**h**) IS2 and (**i**) IS3, when scoring with the breakpoint closeness scheme at different flank thresholds, are shown in similar *F*-score heatmaps.

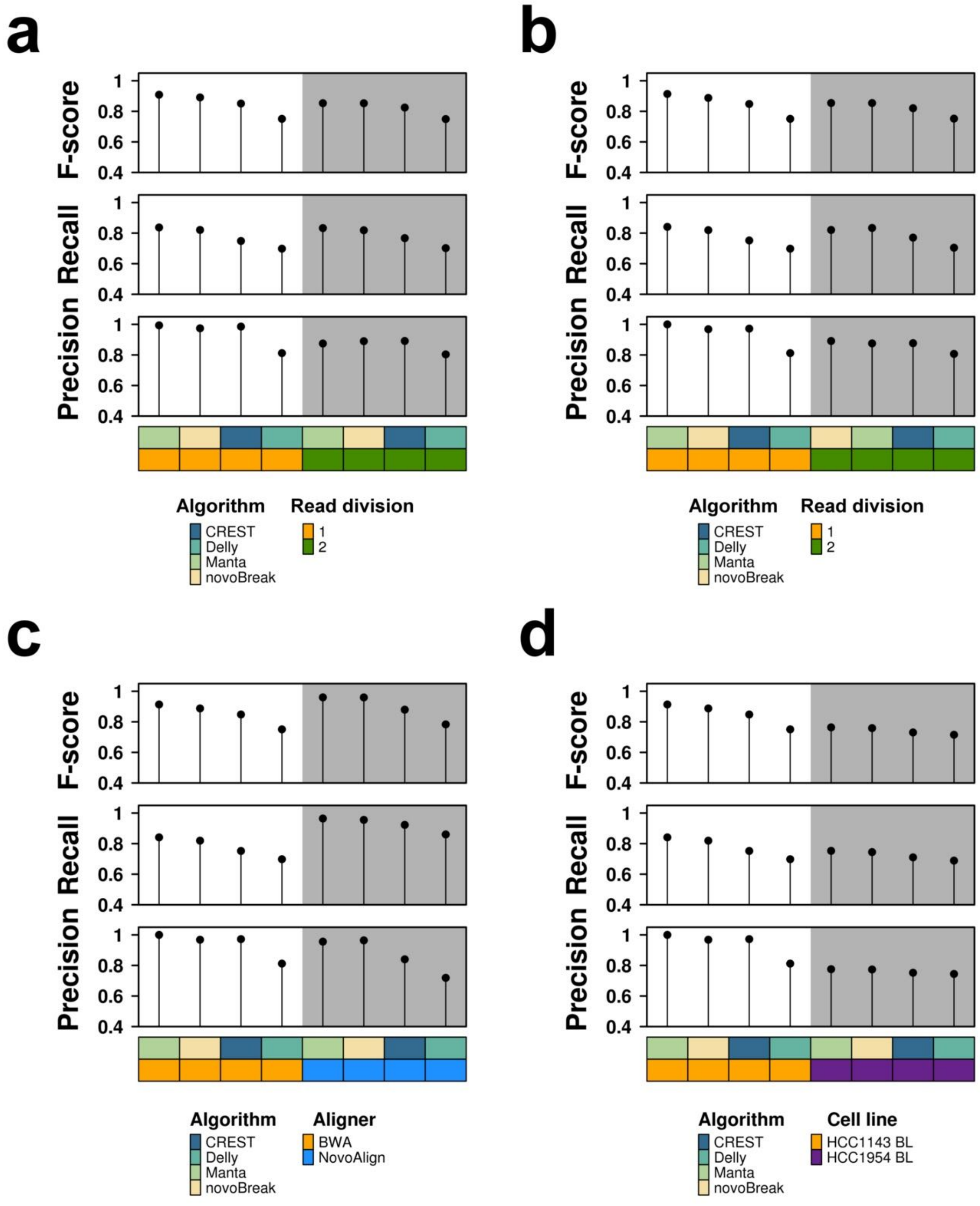

Figure S3 | BAMSurgeon validation (continued).

To test the robustness of simulating somatic SVs with BAMSurgeon with respect to changes in (**a**,**b**) read division, (**c**) aligner and (**d**) cell line, we compared the ranks of CREST, Delly, Manta and novoBreak on three tumour-normal data sets generated with the same set of target mutations, that differ by the above variables. Callers were scored with (**a**) f = 100 bp (Additional file 1: Figure S2b) and (**b-d**) j > 0 (Additional file 1: Figure S2a). With both scoring schemes, caller ordering was independent of read division, aligner and cell line (also see Fig. 1b,c).

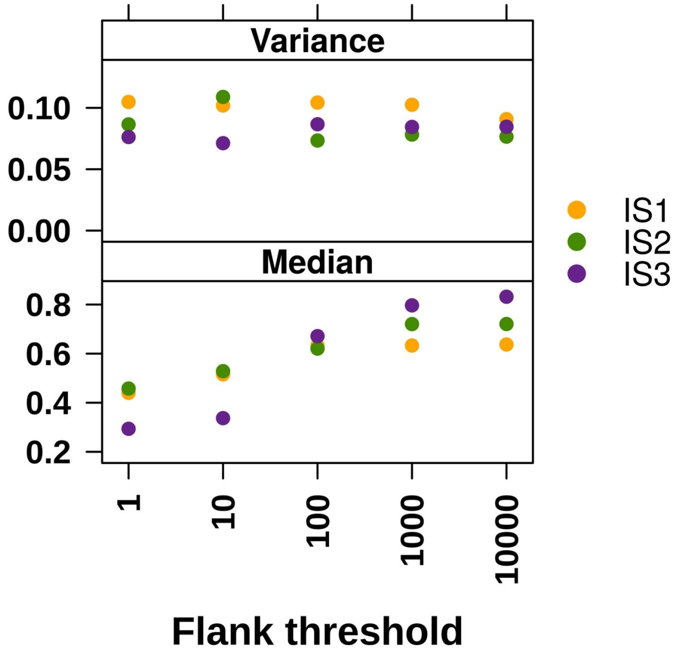

Figure S4 | Characteristics of *F*-scores resulting from different flank thresholds.
The median and variance of the *F*-scores resulting from different flank threshold values are shown for IS1-IS3.

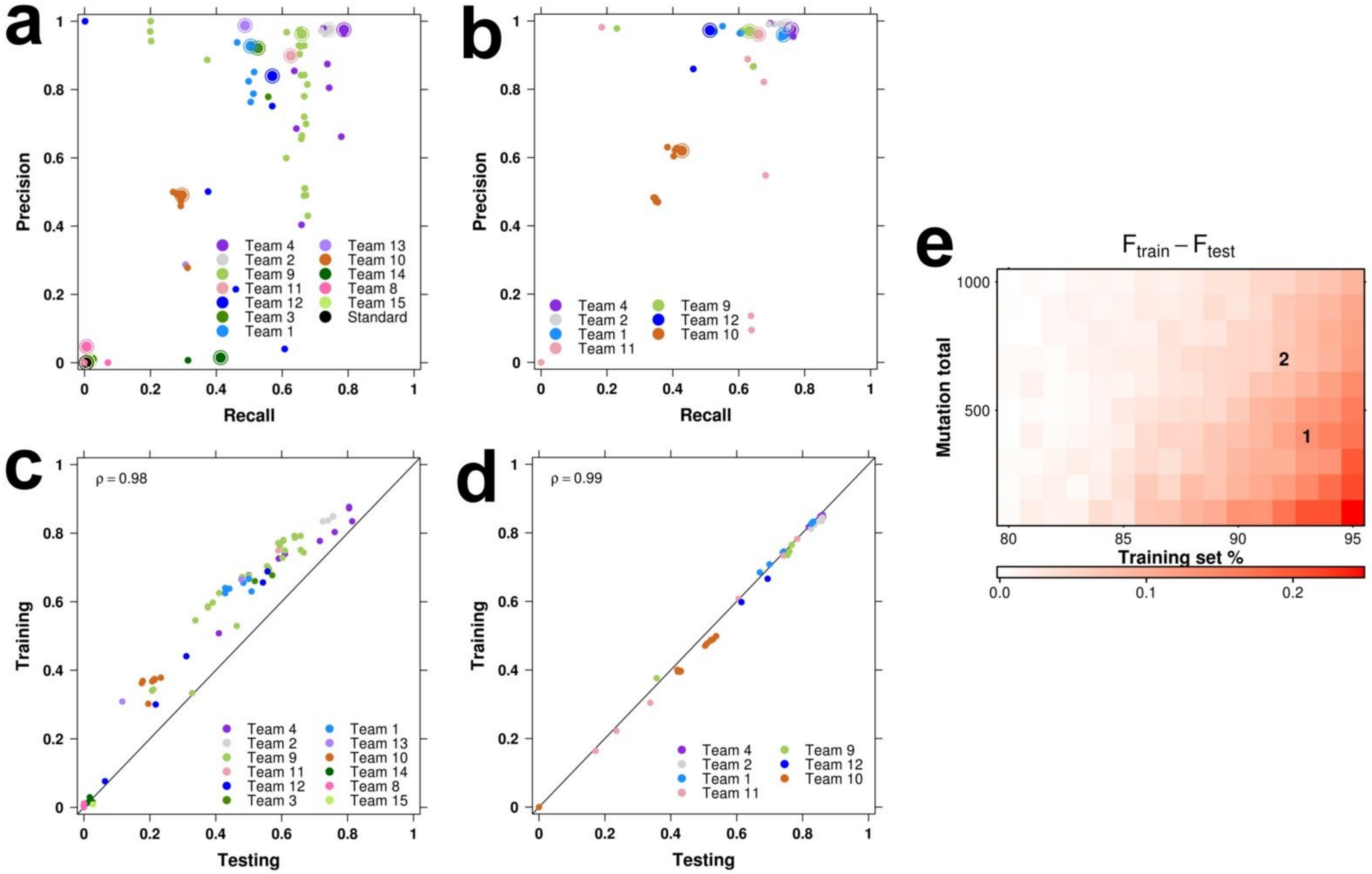
a
b
c
d
e
Precision
Recall
Training
Testing
Team 4
Team 2
Team 9
Team 11
Team 12
Team 3
Team 1
Team 13
Team 10
Team 14
Team 8
Team 15
Standard
ρ = 0.98
ρ = 0.99
$F_{train} - F_{test}$
Mutation total
Training set %

Figure S5 | Overview of SV Calling Challenge submissions (continued).
a,b Precision-recall plots of IS2 and IS3 submissions, respectively. Each point represents a submission, each colour represent a team and the best submission from each team (top *F*-score) is circled. **c,d** The performance of submissions on the training and testing sets are highly correlated for IS2 and IS3, respectively (Spearman's $\rho \geq 0.98$), falling near the plotted $y = x$ line. **a**,**c** The 'Standard' point corresponds to the reference point submission provided by Challenge organizers. **e** Differences in IS3 submission *F*-scores computed on simulated training and testing sets of different sizes. Darker shades of red indicate greater values of (F_{train} - F_{test}), where F_{train} and F_{test} are median *F*-scores on simulated training and testing sets (100 re-samplings for each pair of mutation total and training set percentage values), respectively. The mutation totals and training set percentages used for IS1 and IS2 are labeled with 1 and 2, respectively. For both challenges, the simulated (F_{train} - F_{test}) values suggest overfitting but are an artefact of training set size since no fitting/training was done in this analysis.

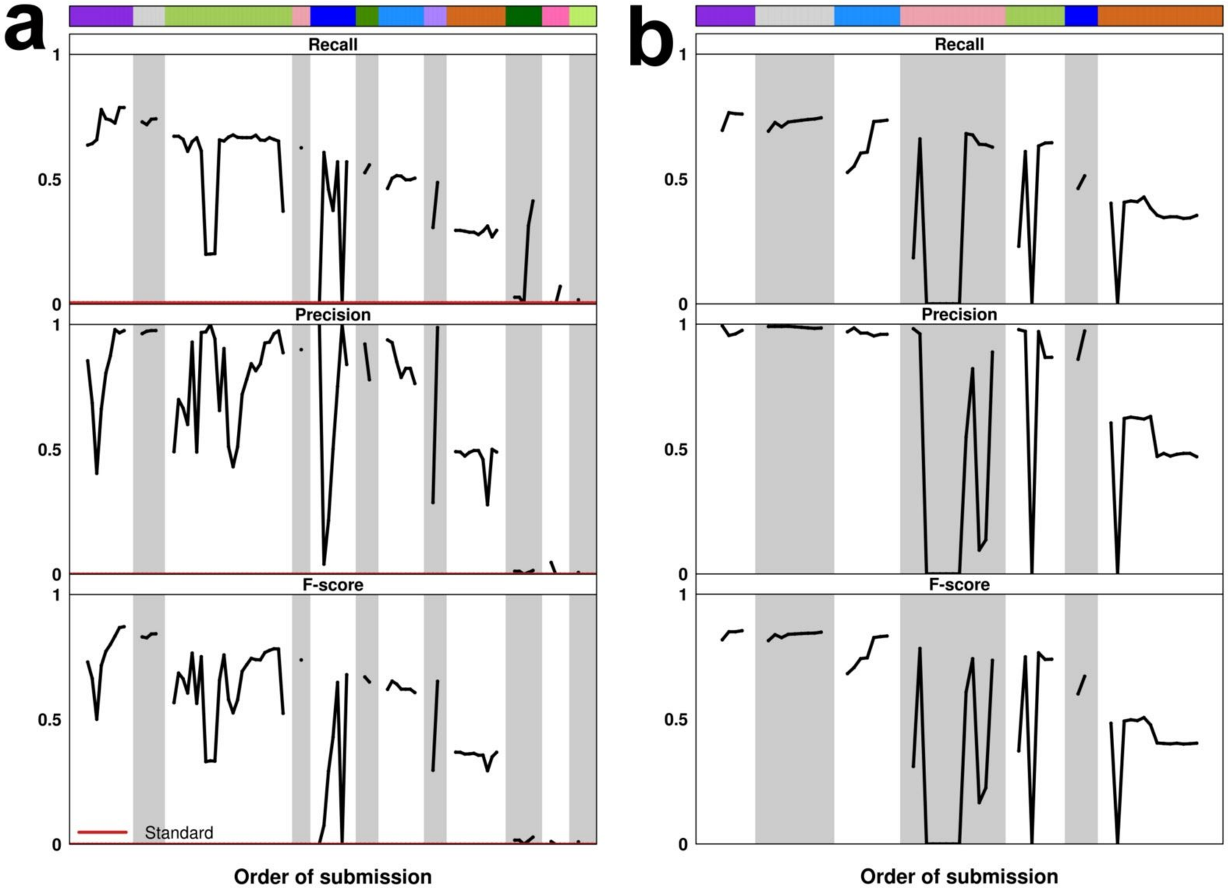

Figure S6 | Performance optimization by parameterization (continued).

Recall, precision and *F*-score of all (**a**) IS2 and (**b**) IS3 submissions plotted by team, then submission order. Teams were ranked by the *F*-score of their best submission, colour coding (top bar) as in Additional file 1: Figure S5a,b. The 'Standard' lines correspond to the reference point submission provided by Challenge organizers.

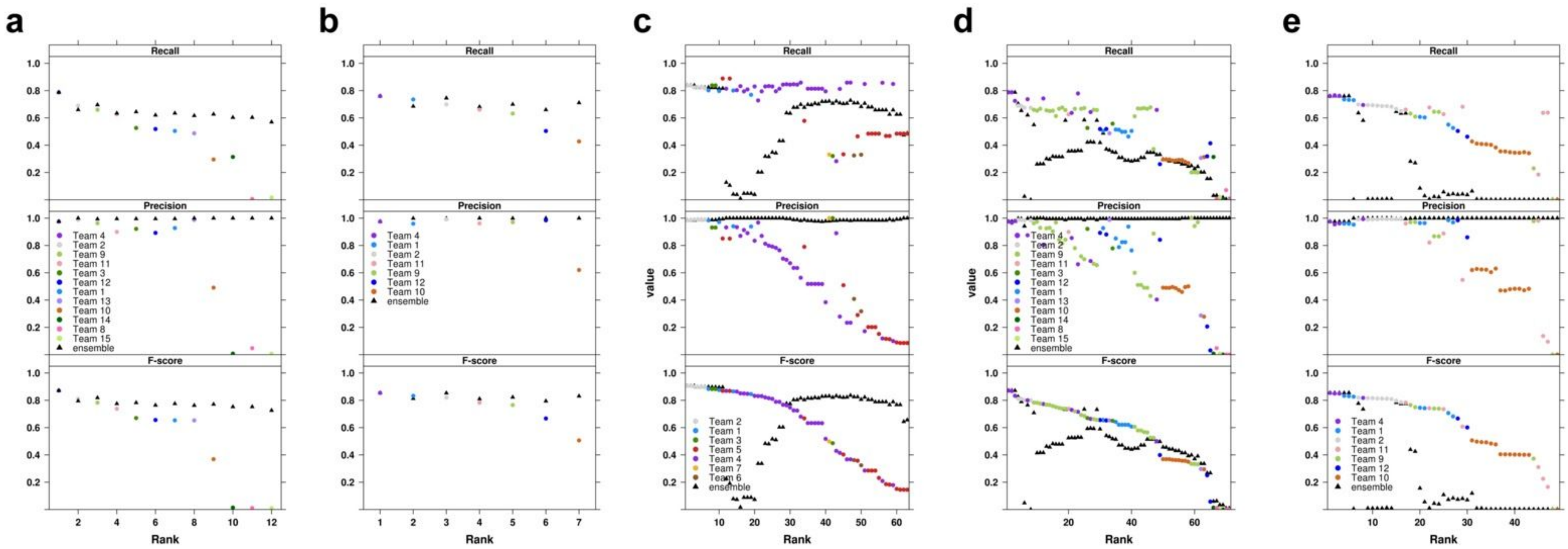

Figure S7 | Performance optimization by ensembles (continued).

Recall, precision and *F*-score of baseline ensembles (see Methods) versus individual submissions for (**a**) IS2 and (**b**) IS3. At the *k*th rank, the triangles indicate performance of the ensemble of the top *k* submissions, and the circles indicate performance of the *k*th ranked submission. The baseline ensembles focused on the best submission from each team. Similar comparisons are shown for the conservative ensembles for (**c**) IS1, (**d**) IS2 and (**e**). IS3. The conservative ensembles considered all submissions (see Methods).

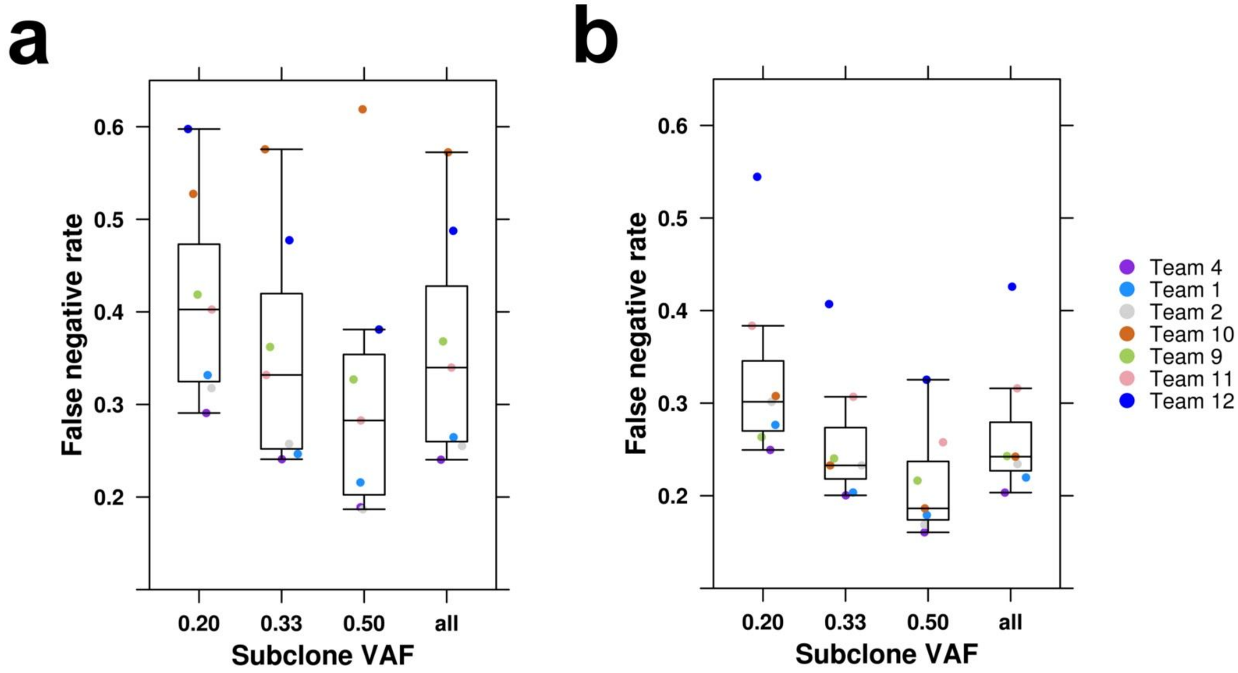

Figure S8 | False negative rate increases with decreasing subclone variant allele frequency (SCVAF).

In IS3, SVs were spiked-in at different SCVAFs, with about a third of SVs at each SCVAF. For the best submission from each team (determined by *F*-score computed on all SVs), the false negative rate was computed on the subset of SVs at a given SCVAF, or all SVs, scored with (**a**) f = 100 bp and (**b**) $j > 0$. Each box indicates the distribution of submission false negative rates and the point colour indicates the team.

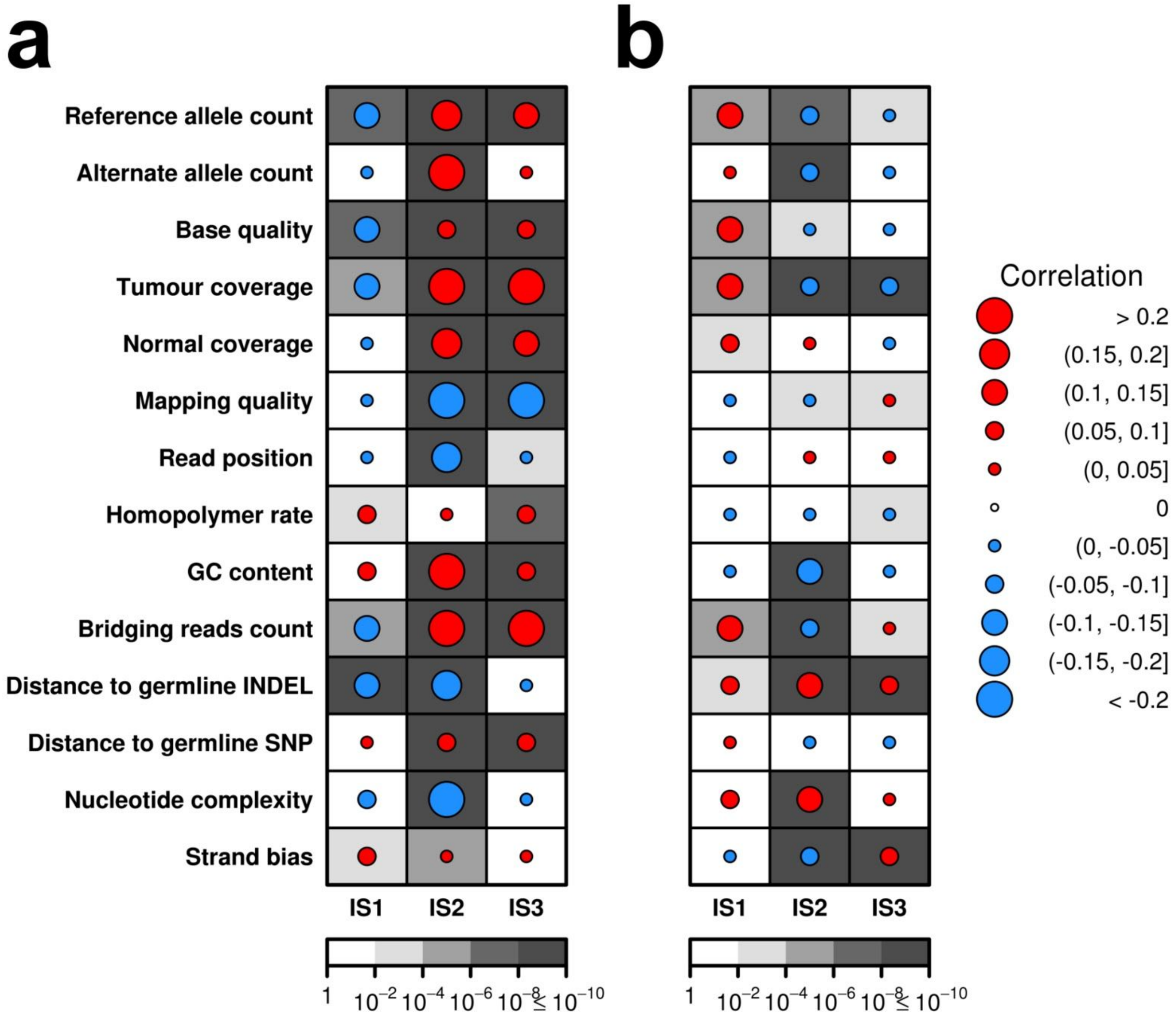

Figure S9 | Associations between sequence-based variables and the number of prediction errors.

Each row indicates a variable that describes the sequence at a given breakpoint and each column indicates an *in silico* tumour. The size of the dot reflects the magnitude of the Pearson correlation between a given variable and the number of teams (focusing on the best submission per team) with a FP (**a**) or FN (**b**) at various breakpoints in the given tumour. Darker background shading indicates more significant correlations (see colour bar at the bottom). Abbreviations: SNP, single-nucleotide polymorphism; INDEL, short insertion or deletion.

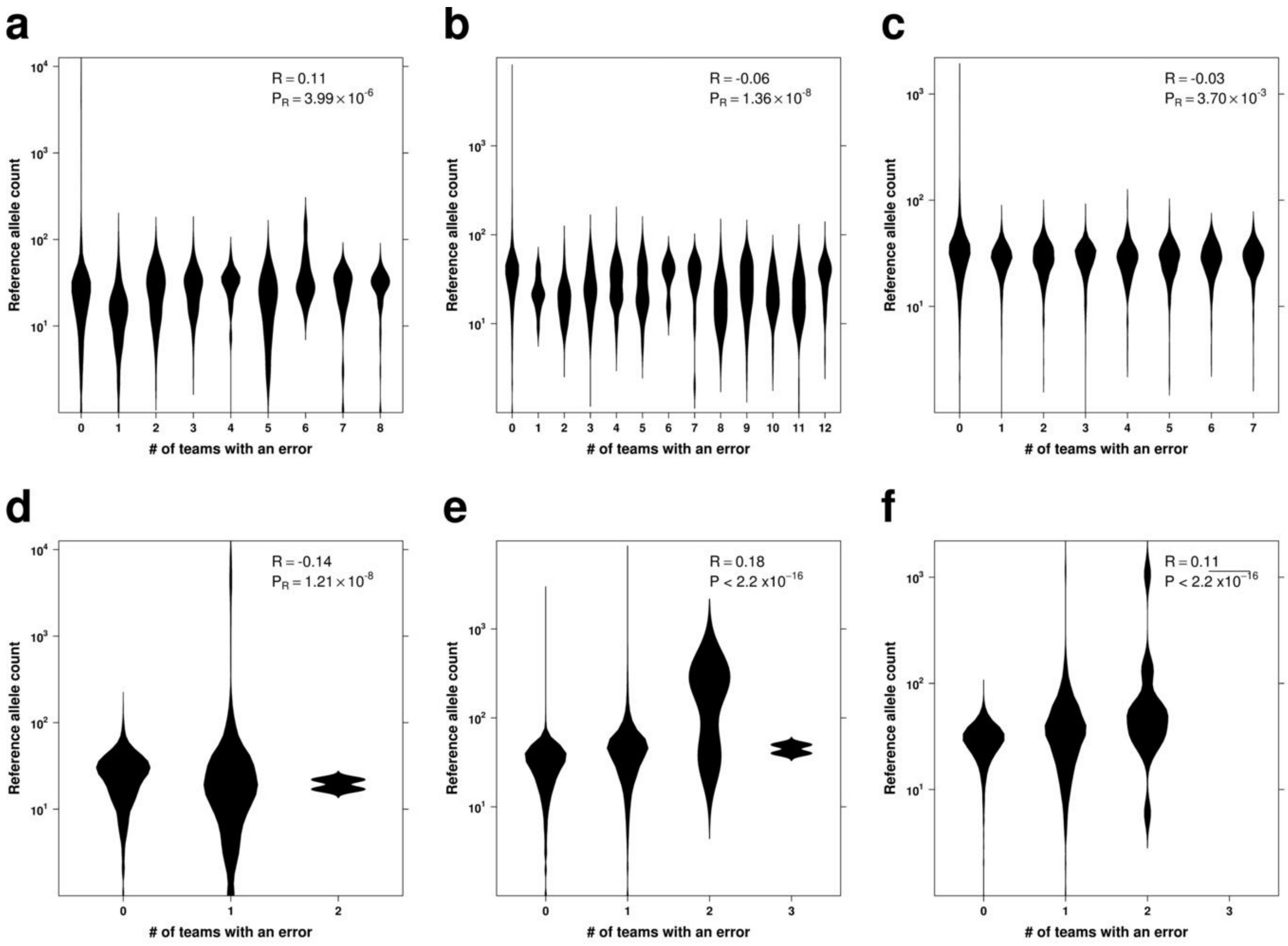

Figure S10 | Associations between reference allele count and the number of prediction errors.
Violin plots show the relationship between reference allele count and the number of teams (focusing on the best submission per team) with a FN (**a-c**) or FP (**d-f**) at various breakpoints in IS1 (**a,d**), IS2 (**b,e**), and IS3 (**c,f**). Pearson correlation between the counts and error rates are shown, together with the corresponding *P*-values.

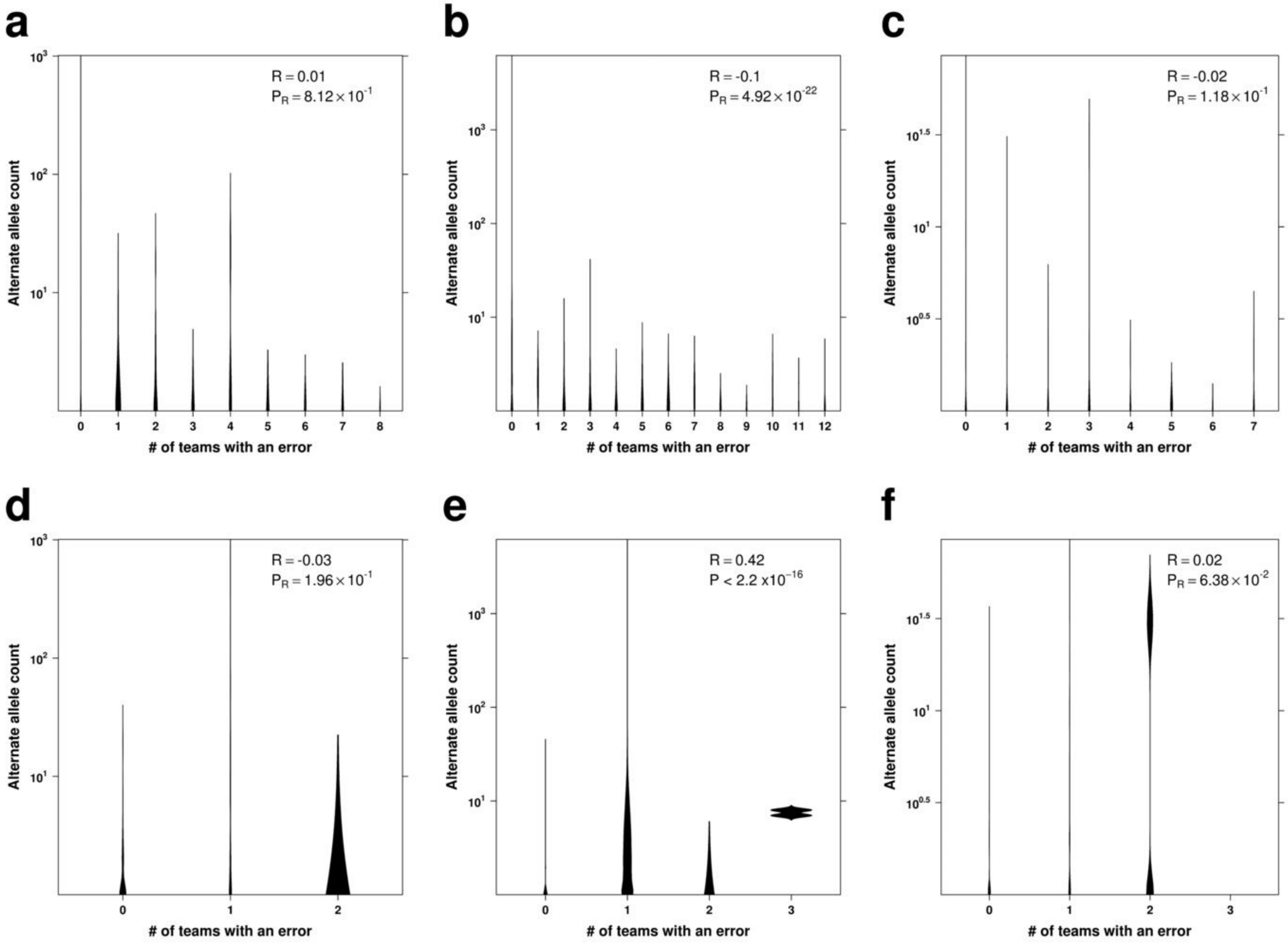

Figure S11 | Associations between alternate allele count and the number of prediction errors.
Violin plots show the relationship between alternate allele count and the number of teams (focusing on the best submission per team) with a FN (**a-c**) or FP (**d-f**) at various breakpoints in IS1 (**a,d**), IS2 (**b,e**), and IS3 (**c,f**). Pearson correlations between the counts and error rates are shown, together with the corresponding *P*-values.

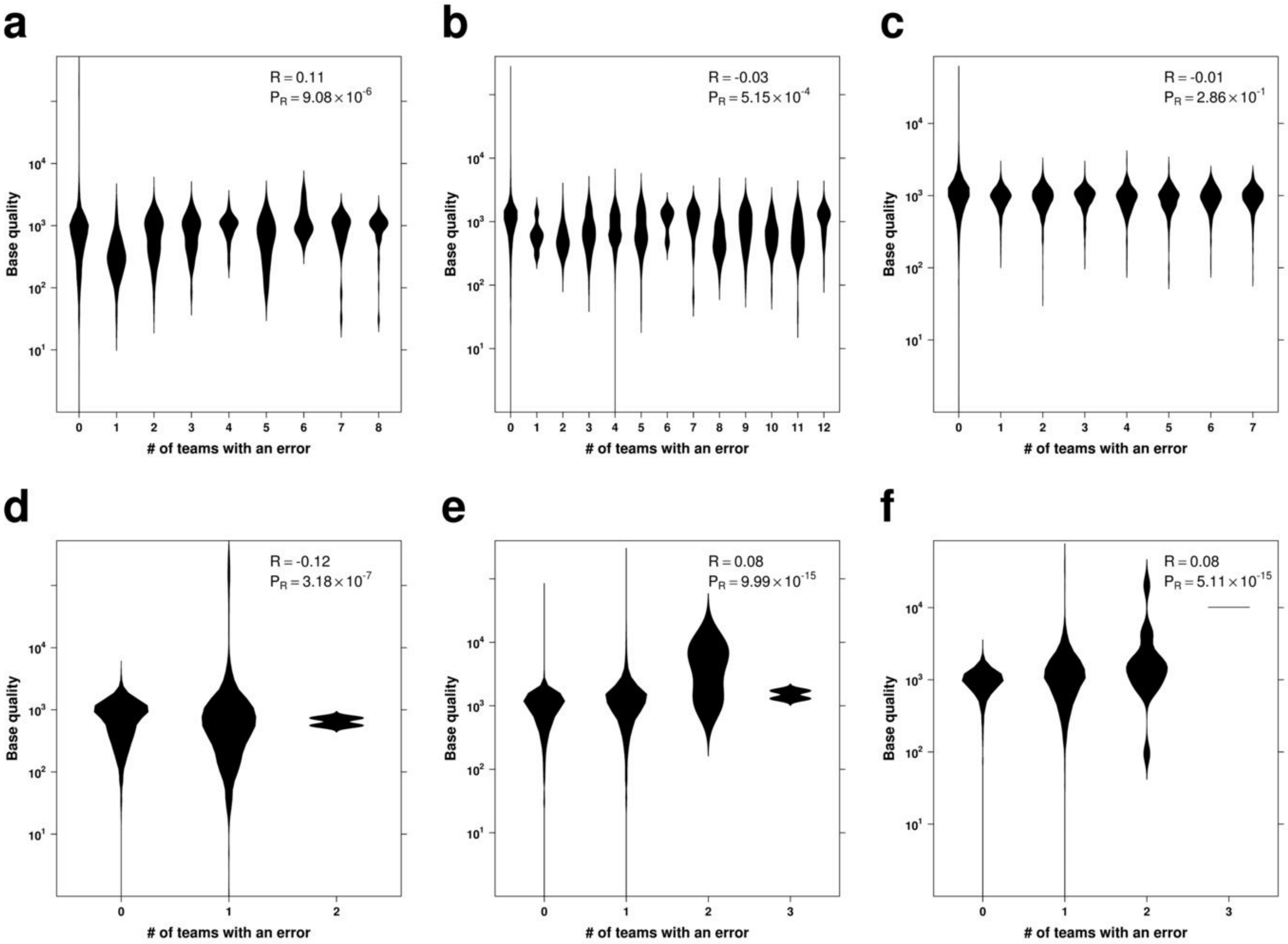

Figure S12 | Associations between base quality and the number of prediction errors.
Violin plots show the relationship between base quality and the number of teams (focusing on the best submission per team) with a FN (**a-c**) or FP (**d-f**) at various breakpoints in IS1 (**a,d**), IS2 (**b,e**), and IS3 (**c,f**). Pearson correlations between the quality values and error rates are shown, together with the corresponding *P*-values.

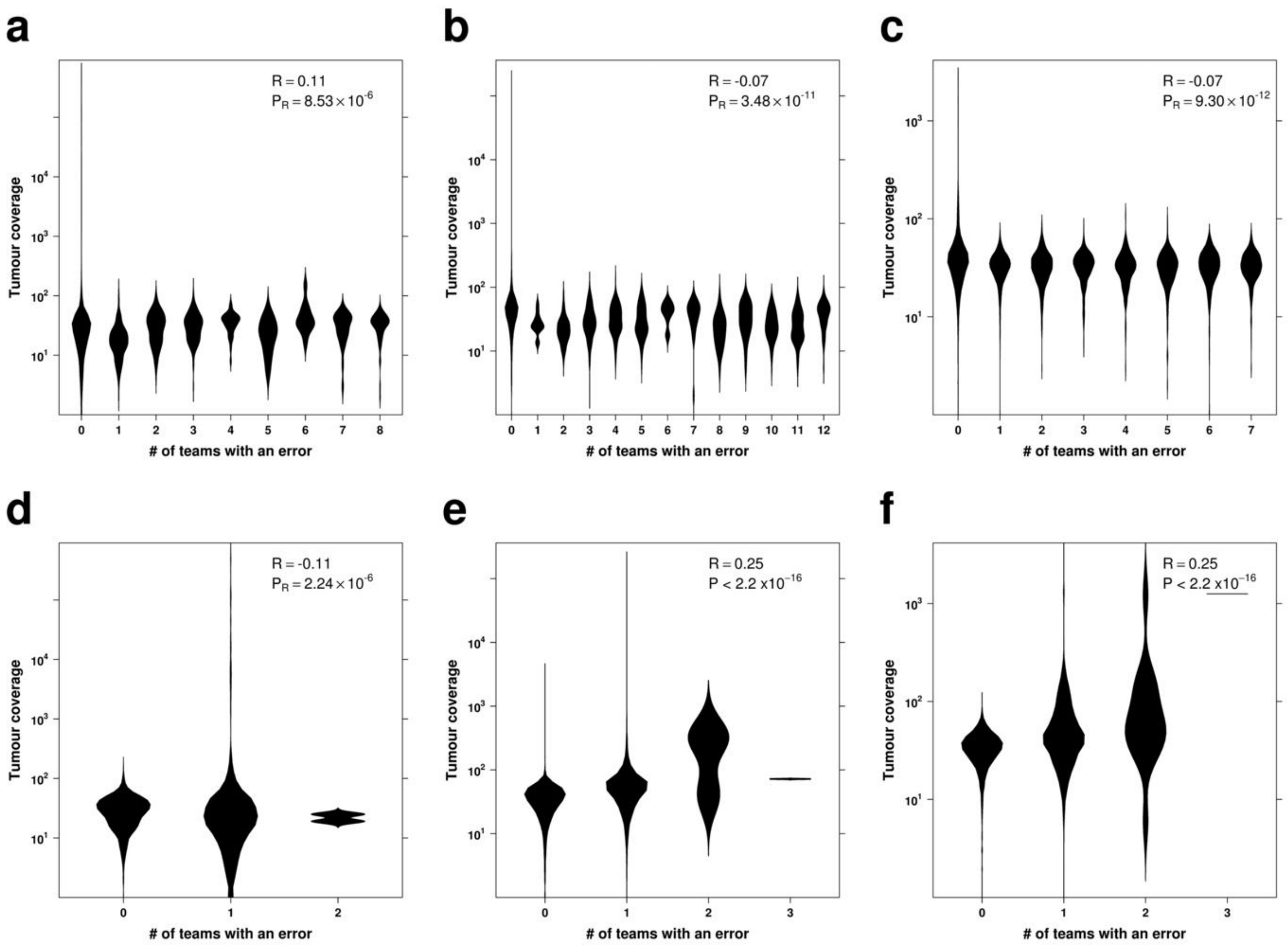

Figure S13 | Associations between tumour coverage and the number of prediction errors.
Violin plots show the relationship between tumour coverage and the number of teams (focusing on the best submission per team) with a FN (**a-c**) or FP (**d-f**) at various breakpoints in IS1 (**a,d**), IS2 (**b,e**), and IS3 (**c,f**). Pearson correlations between the coverage values and error rates is shown, together with the corresponding *P*-values.

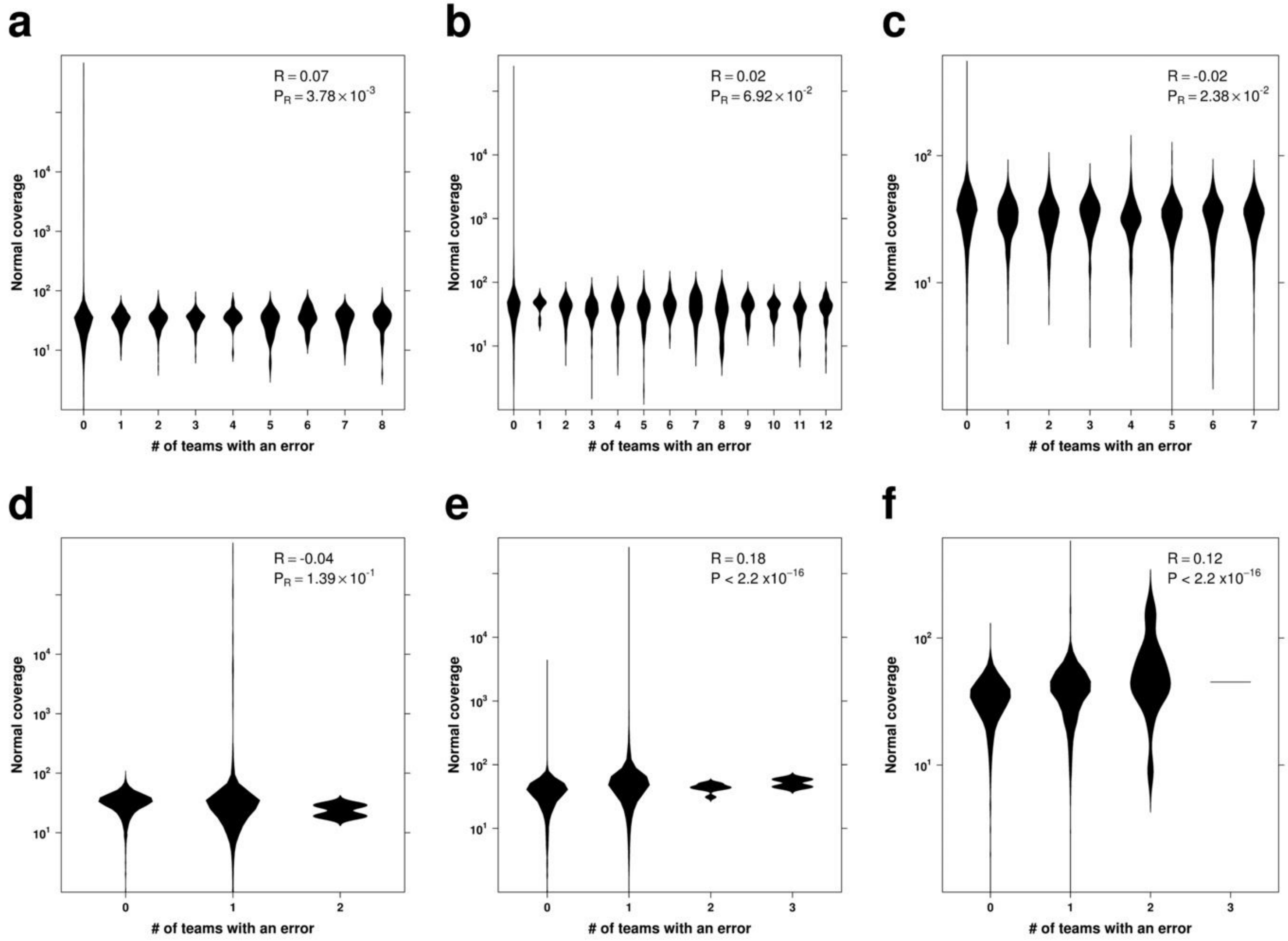

Figure S14 | Associations between normal coverage and the number of prediction errors.
Violin plots show the relationship between normal coverage and the number of teams (focusing on the best submission per team) with a FN (**a-c**) or FP (**d-f**) at various breakpoints in IS1 (**a,d**), IS2 (**b,e**), and IS3 (**c,f**). Pearson correlations between the coverage values and error rates is shown, together with the corresponding *P*-values.

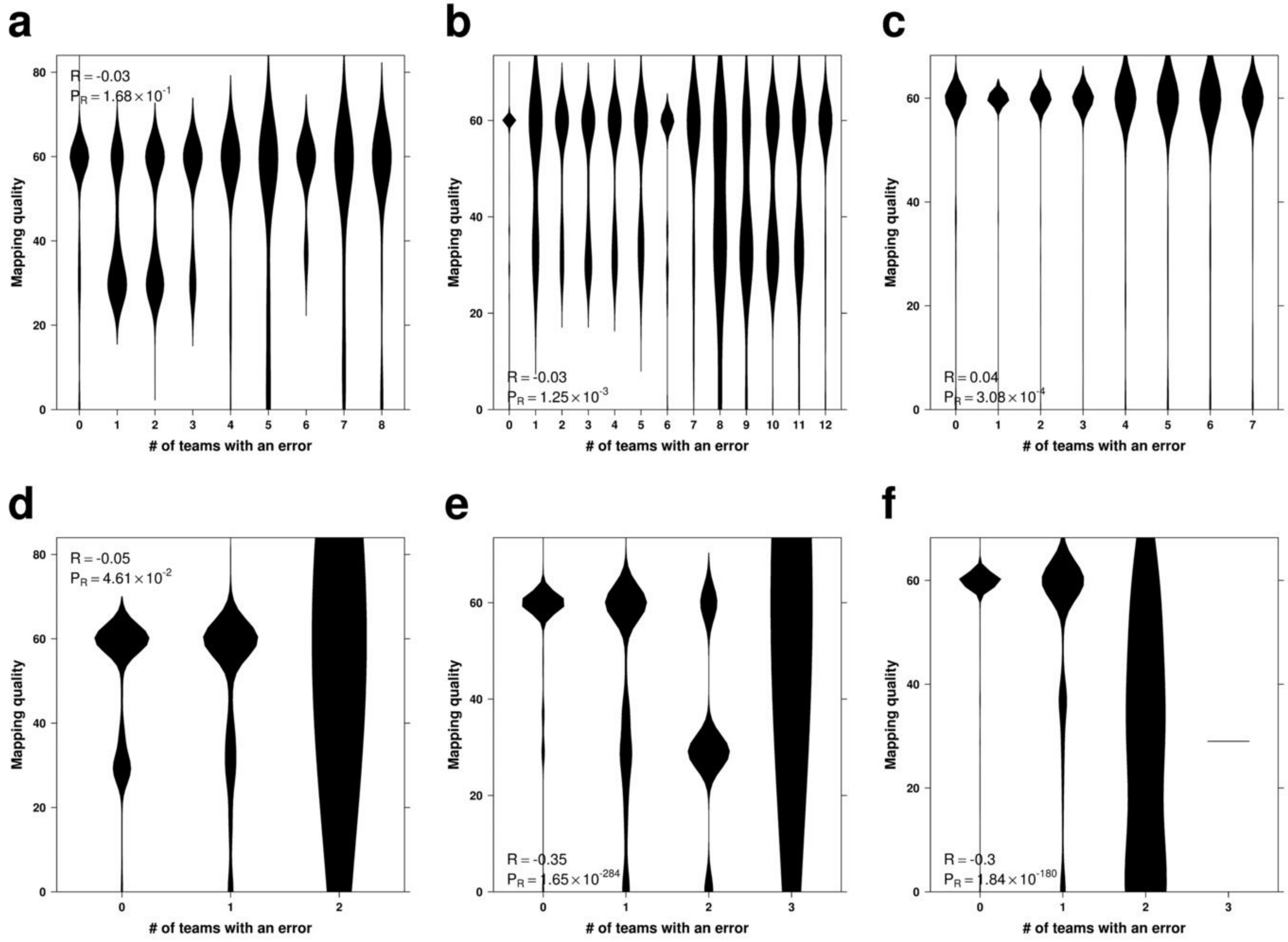

Figure S15 | Associations between map quality and the number of prediction errors.
Violin plots show the relationship between map quality and the number of teams (focusing on the best submission per team) with a FN (**a-c**) or FP (**d-f**) at various breakpoints in IS1 (**a,d**), IS2 (**b,e**), and IS3 (**c,f**). Pearson correlations between the quality values and error rates are shown, together with the corresponding *P*-values.

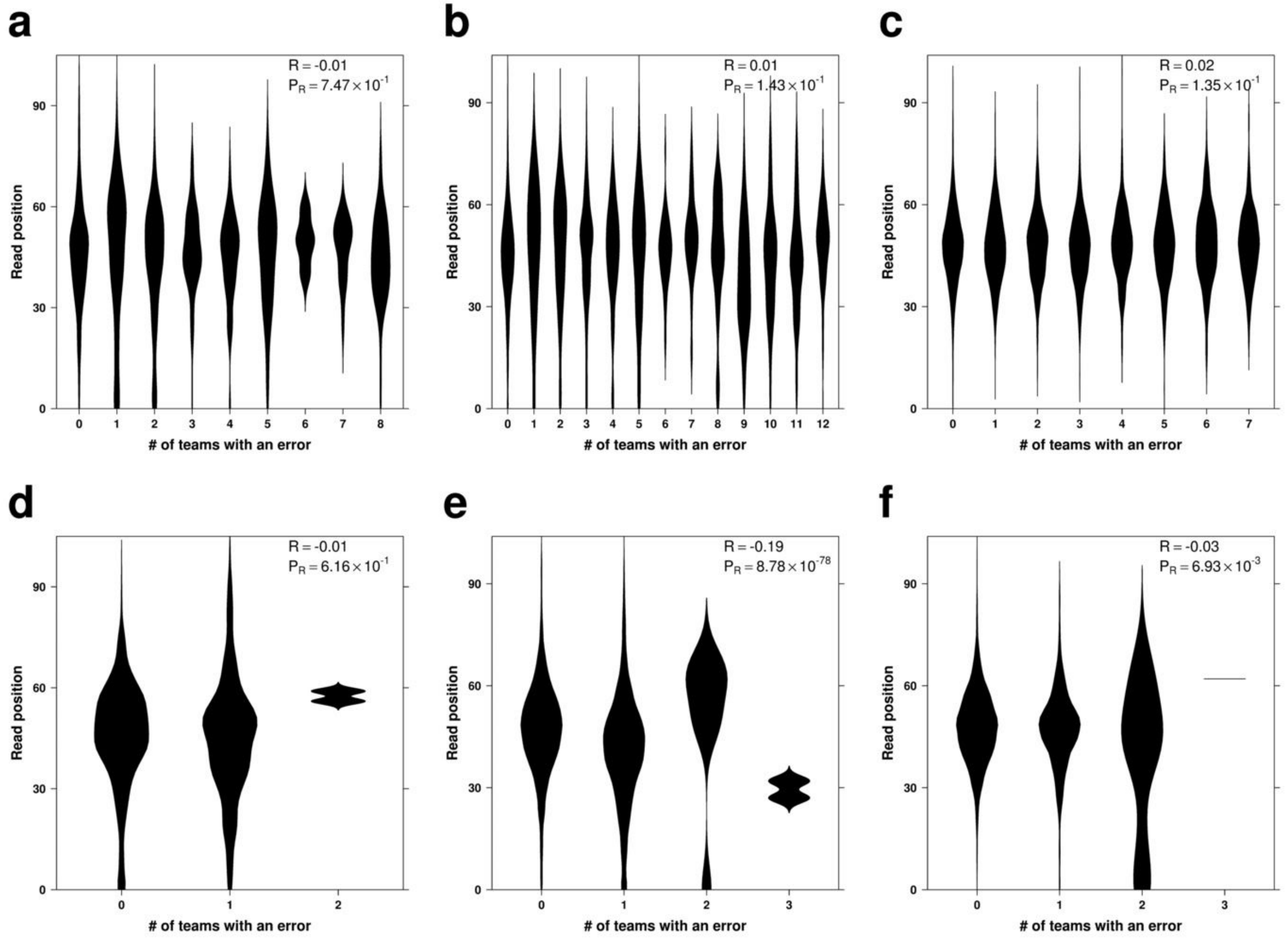

Figure S16 | Associations between read position and the number of prediction errors.
Violin plots show the relationship between read position and the number of teams (focusing on the best submission per team) with a FN (**a-c**) or FP (**d-f**) at various breakpoints in IS1 (**a,d**), IS2 (**b,e**), and IS3 (**c,f**). Pearson correlations between the positions and error rates are shown, together with the corresponding *P*-values.

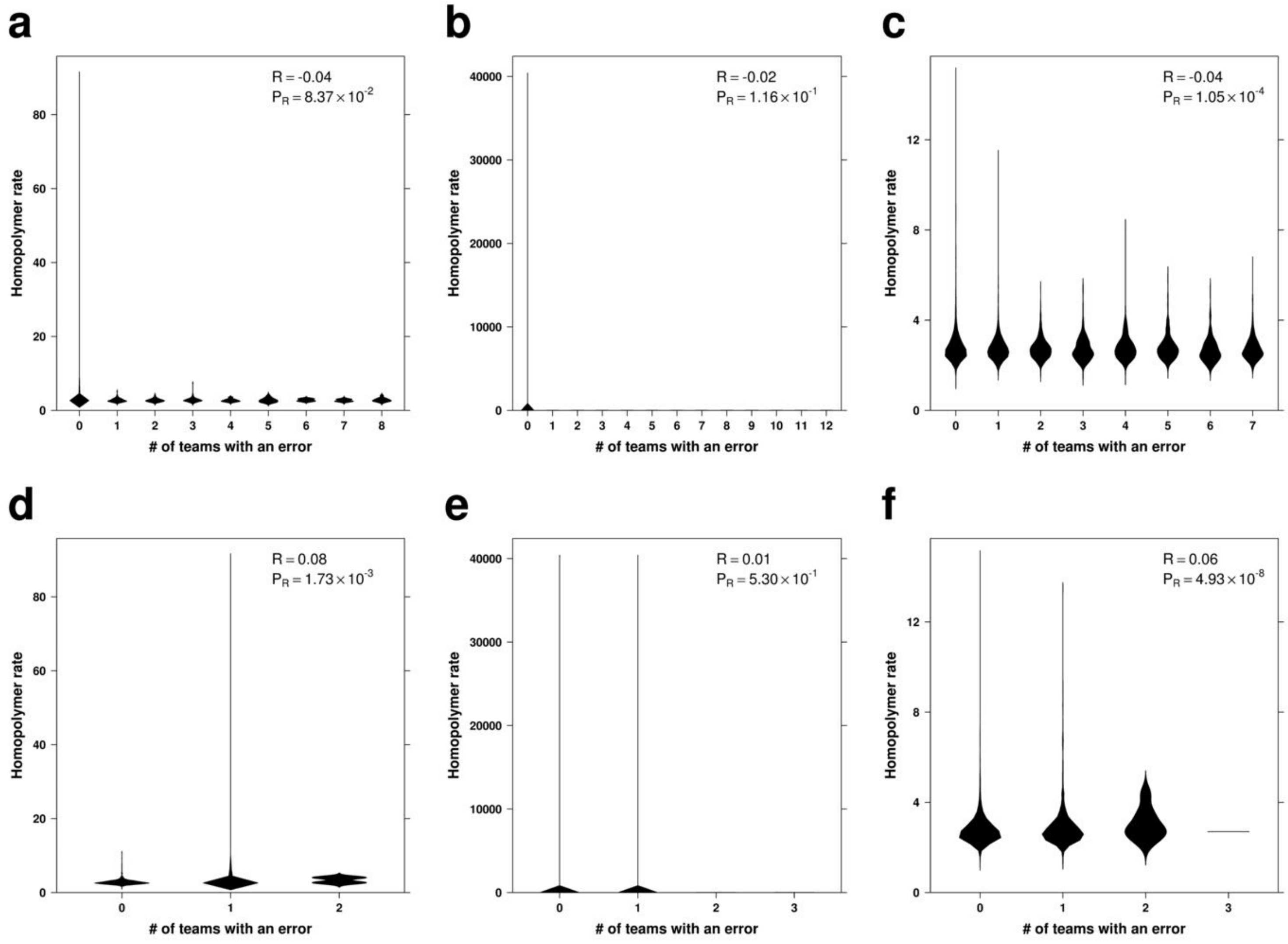

Figure S17 | Associations between homopolymer rate and the number of prediction errors.
Violin plots show the relationship between homopolymer rate and the number of teams (focusing on the best submission per team) with a FN (**a-c**) or FP (**d-f**) at various breakpoints in IS1 (**a,d**), IS2 (**b,e**), and IS3 (**c,f**). Pearson correlations between the homopolymer and error rates are shown, together with the corresponding *P*-values.

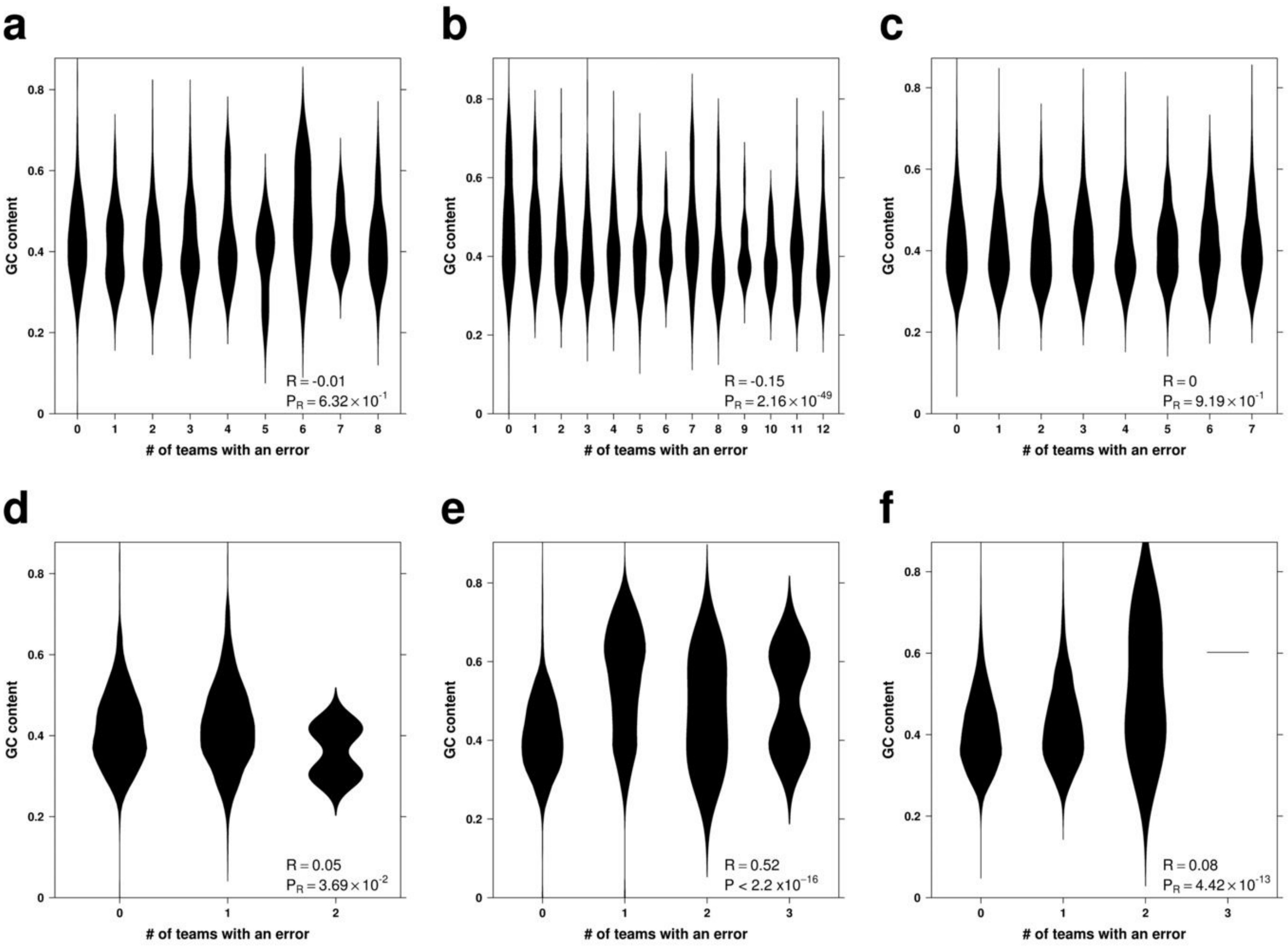

Figure S18 | Associations between GC content and the number of prediction errors.
Violin plots show the relationship between GC content and the number of teams (focusing on the best submission per team) with a FN (**a-c**) or FP (**d-f**) at various breakpoints in IS1 (**a,d**), IS2 (**b,e**), and IS3 (**c,f**). Pearson correlations between the GC content values and error rates are shown, together with the corresponding *P*-values.

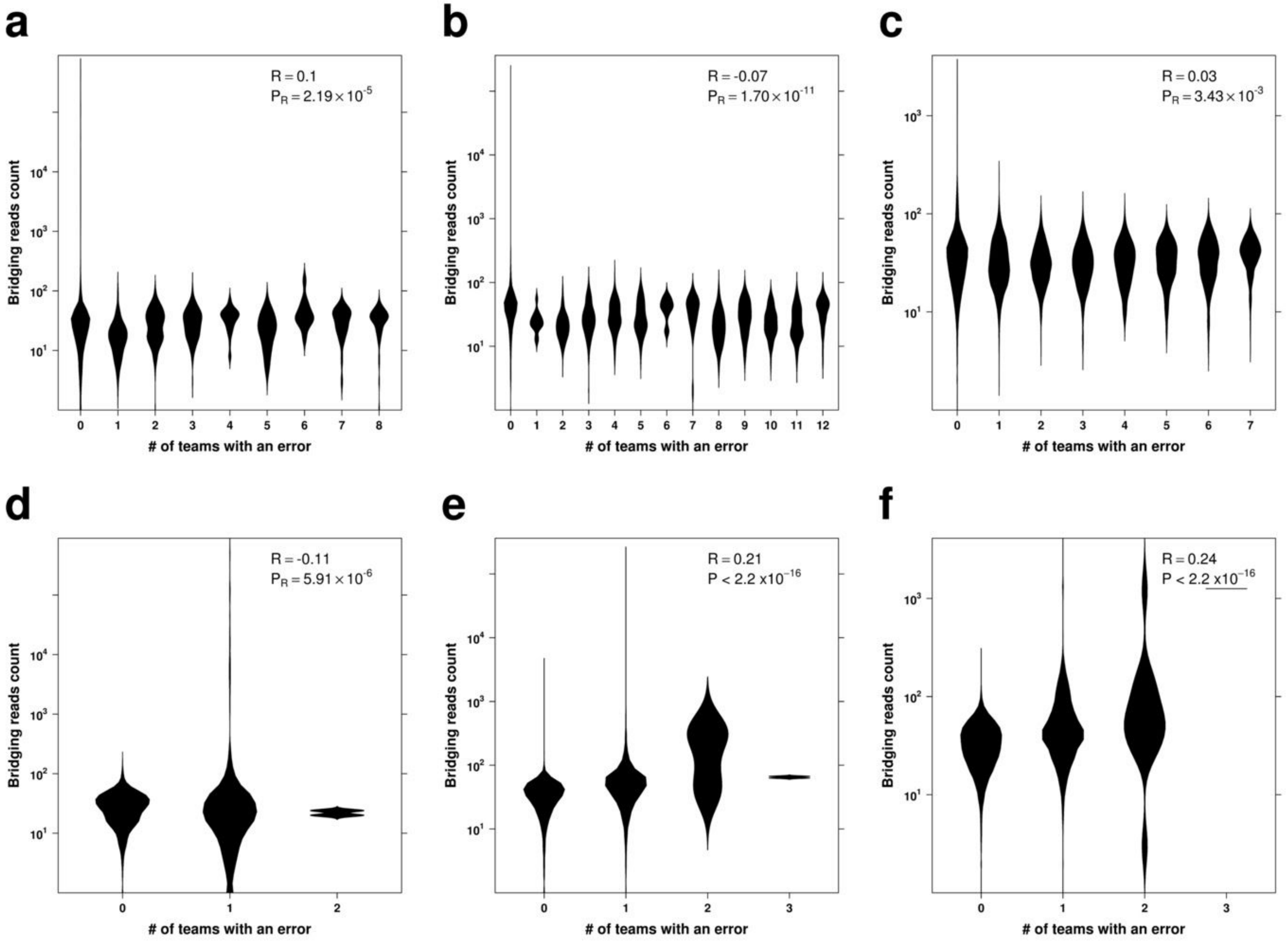

Figure S19 | Associations between bridging read count and the number of prediction errors.
Violin plots show the relationship between bridging read count and the number of teams (focusing on the best submission per team) with a FN (**a-c**) or FP (**d-f**) at various breakpoints in IS1 (**a,d**), IS2 (**b,e**), and IS3 (**c,f**). Pearson correlations between the counts and error rates are shown, together with the corresponding *P*-values.

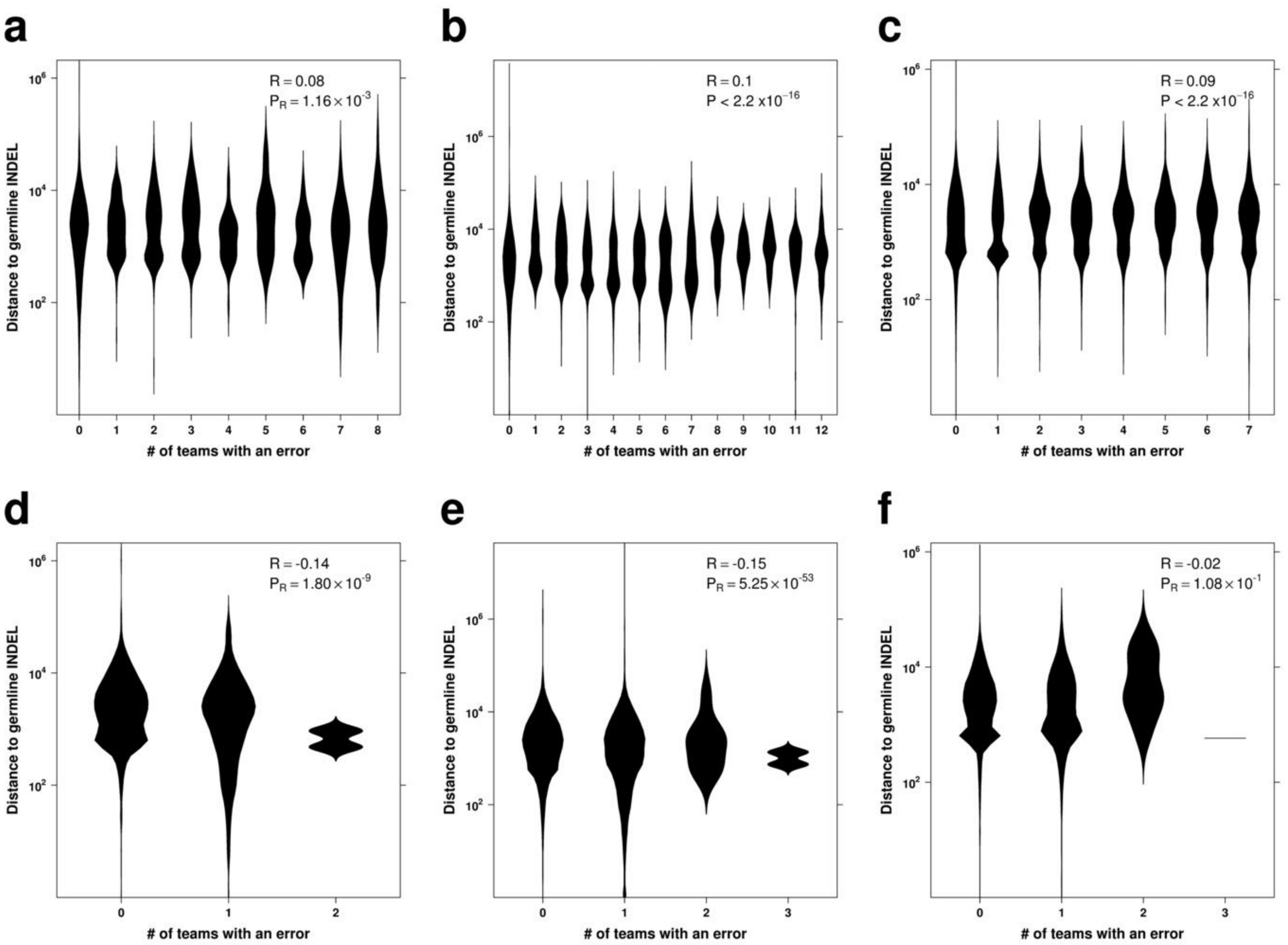

Figure S20 | Associations between distance to germline INDEL and the number of prediction errors.
Violin plots show the relationship between distance to the nearest germline INDEL and the number of teams (focusing on the best submission per team) with a FN (**a-c**) or FP (**d-f**) at various breakpoints in IS1 (**a,d**), IS2 (**b,e**), and IS3 (**c,f**). Pearson correlations between the distances and error rates are shown, together with the corresponding *P*-values. Abbreviation: INDEL, short insertion or deletion.

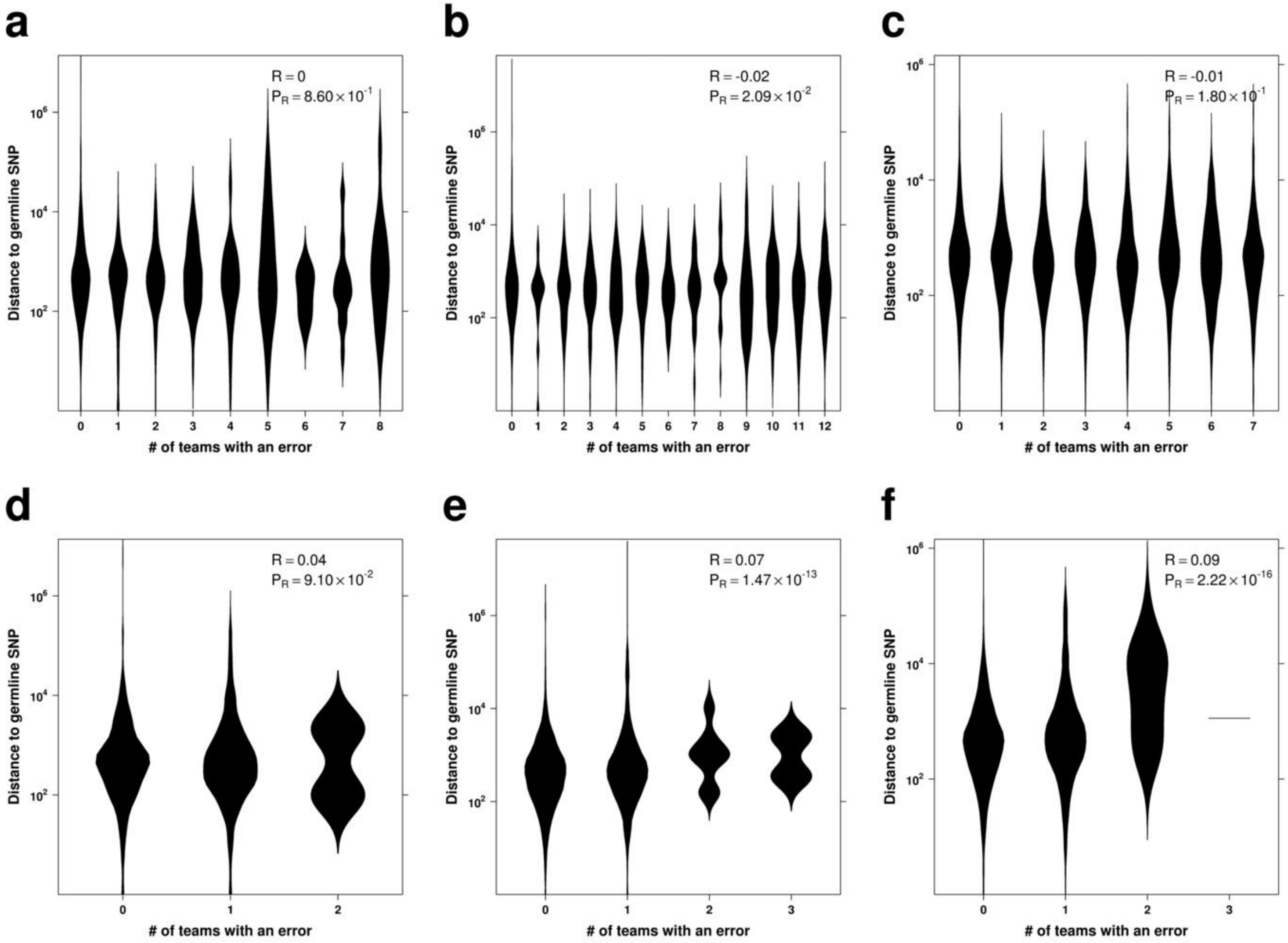

Figure S21 | Associations between distance to germline SNP and the number of prediction errors.
Violin plots show the relationship between distance to the nearest germline SNP and the number of teams (focusing on the best submission per team) with a FN (**a-c**) or FP (**d-f**) at various breakpoints in IS1 (**a,d**), IS2 (**b,e**), and IS3 (**c,f**). Pearson correlations between the distances and error rates are shown, together with the corresponding *P*-values. Abbreviation: SNP, single-nucleotide polymorphism.

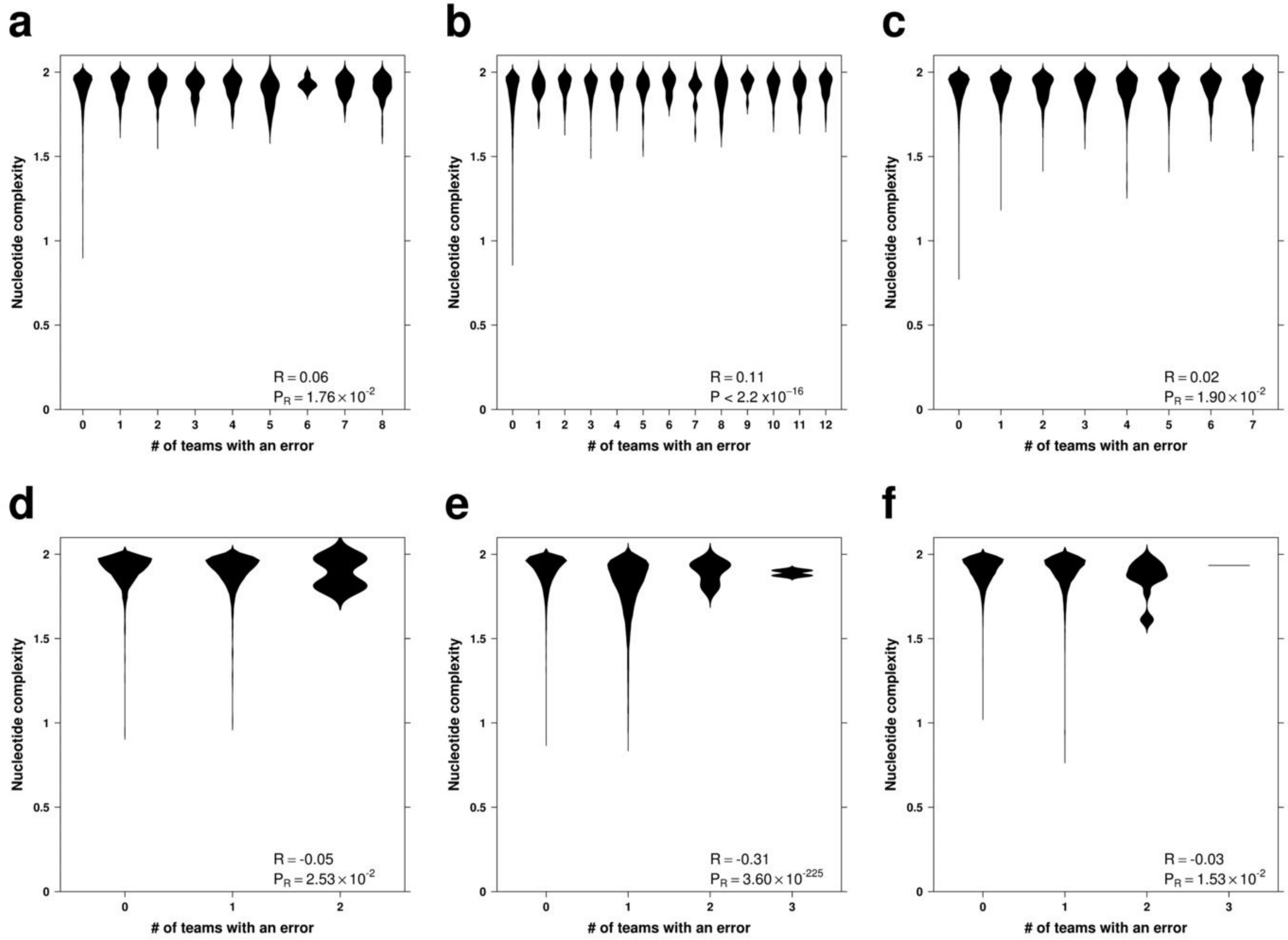

Figure S22 | Associations between nucleotide complexity and the number of prediction errors.
Violin plots show the relationship between nucleotide complexity and the number of teams (focusing on the best submission per team) with a FN (**a-c**) or FP (**d-f**) at various breakpoints in IS1 (**a,d**), IS2 (**b,e**), and IS3 (**c,f**). Pearson correlations between the complexity values and error rates are shown, together with the corresponding *P*-values.

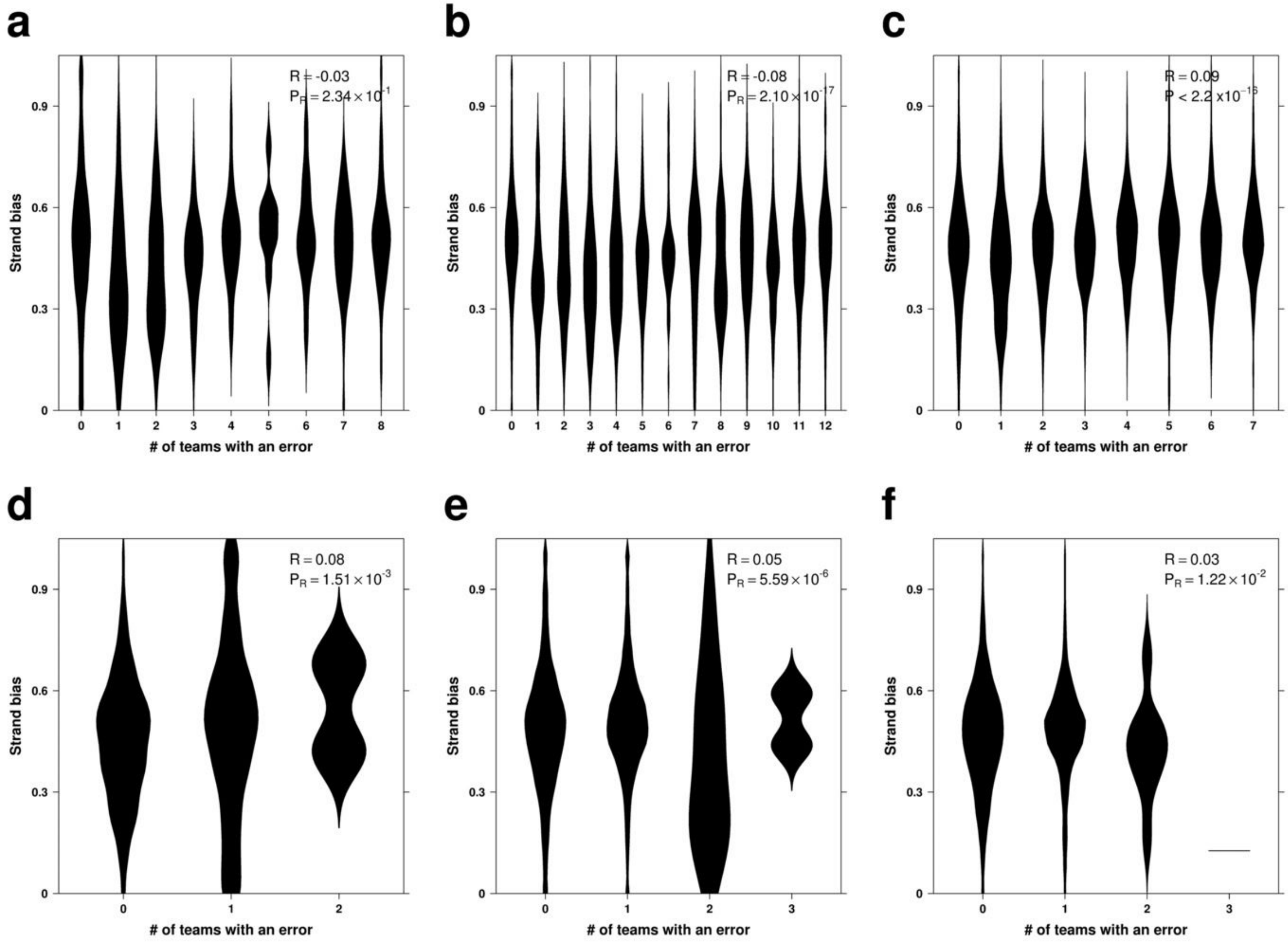

Figure S23 | Associations between strand bias and the number of prediction errors.
Violin plots show the relationship between strand bias and the number of teams (focusing on the best submission per team) with a FN (**a-c**) or FP (**d-f**) at various breakpoints in IS1 (**a,d**), IS2 (**b,e**), and IS3 (**c,f**). Pearson correlations between the bias values and error rates are shown, together with the corresponding *P*-values.

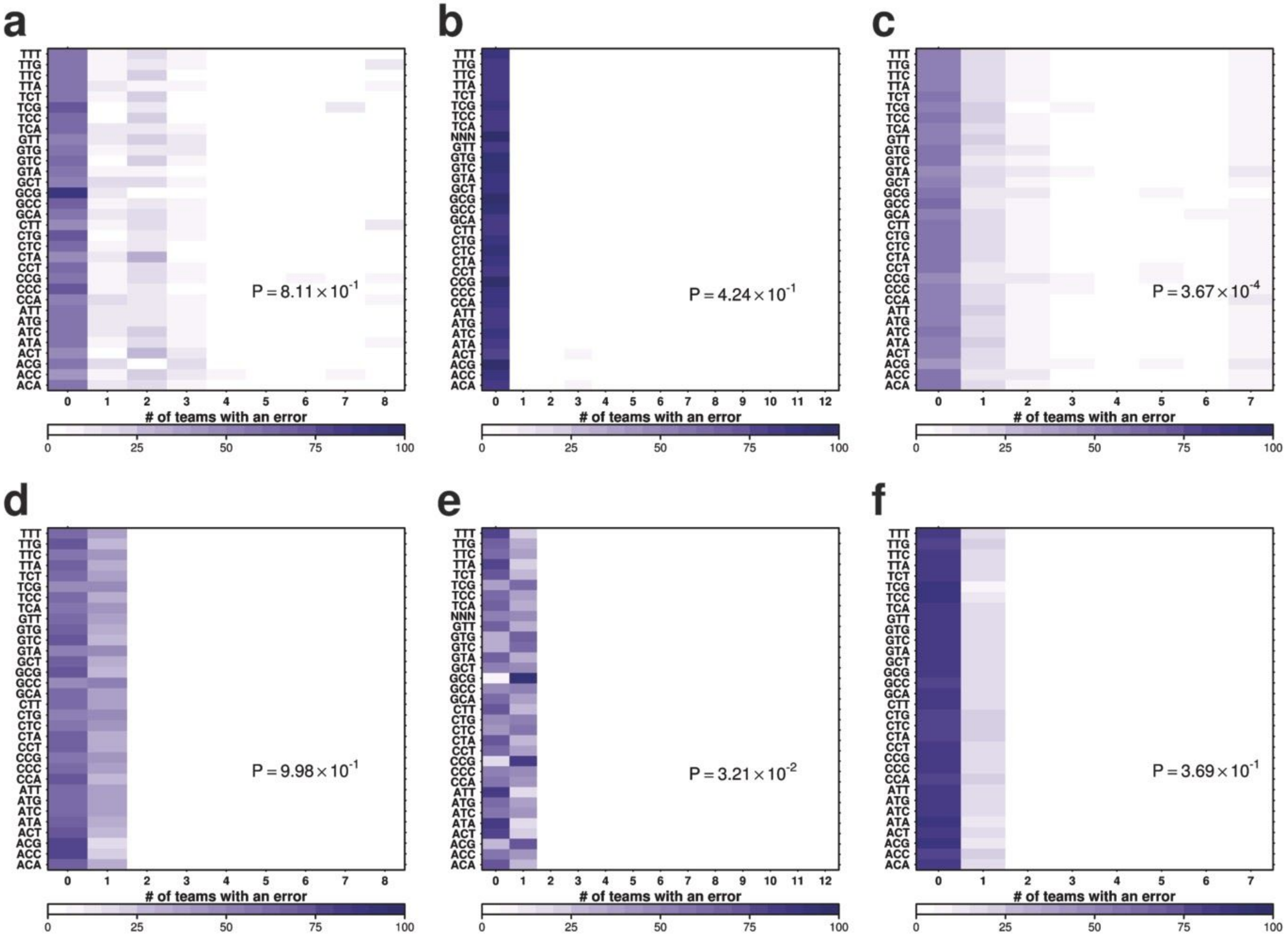

Figure S24 | Associations between trinucleotide and the number of prediction errors.
Heatmaps show the relationship between trinucleotide and the number of teams (focusing on the best submission per team) with a FN (**a-c**) or FP (**d-f**) at various breakpoints in IS1 (**a,d**), IS2 (**b,e**), and IS3 (**c,f**). Darker shades indicate greater percentages of breakpoints, with the trinucleotide indicated by the row, have the number of teams with an error indicated by the column (see colour bar at the bottom). The *P*-value estimating the significance of the association between the trinucleotides and error rates (obtained from a fitted binomial model) is shown.

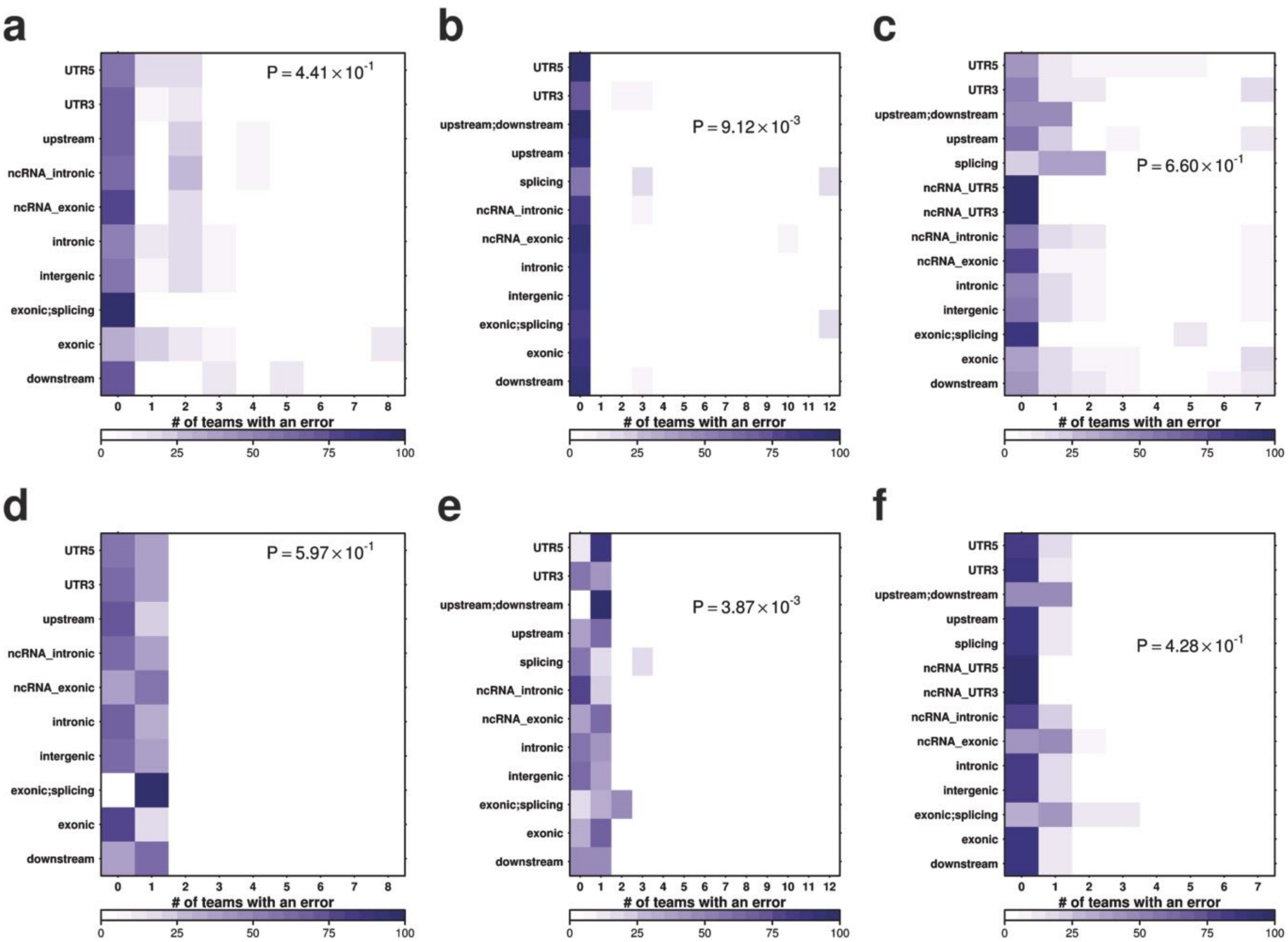

Figure S25 | Associations between genomic location and the number of prediction errors.
Heatmaps show the relationship between genomic location and the number of teams (focusing on the best submission per team) with a FN (**a-c**) or FP (**d-f**) at various breakpoints in IS1 (**a,d**), IS2 (**b,e**), and IS3 (**c,f**). Darker shades indicate greater percentages of breakpoints, with the genomic location indicated by the row, have the number of teams with an error at the breakpoint indicated by the column (see colour bar at the bottom). The *P*-value estimating the significance of the association between the genomic locations and error rates (obtained from a fitted binomial model) is shown.

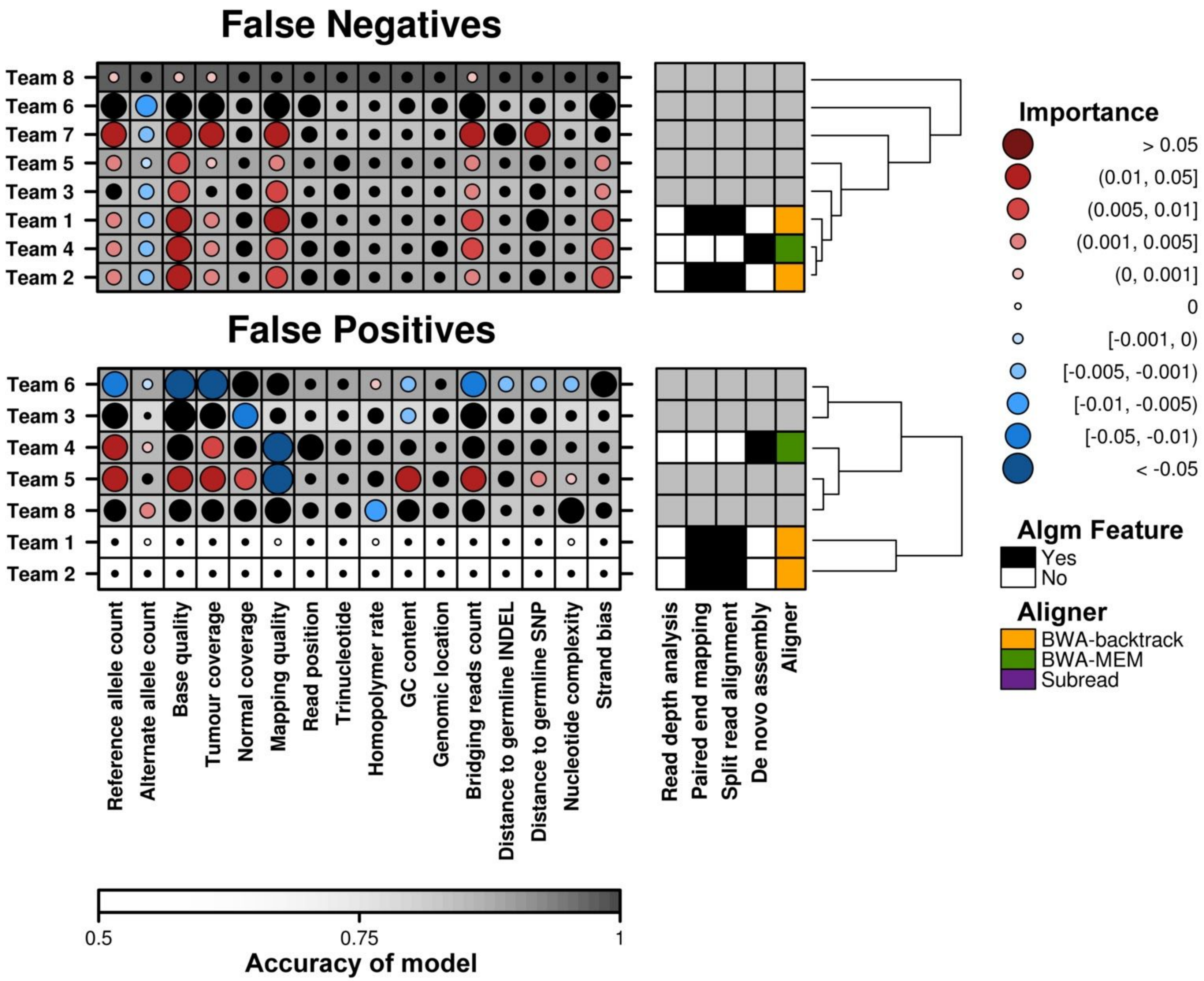

False Negatives
Team 8
Team 6
Team 7
Team 5
Team 3
Team 1
Team 4
Team 2
False Positives
Team 6
Team 3
Team 4
Team 5
Team 8
Team 1
Team 2
Reference allele count
Alternate allele count
Base quality
Tumour coverage
Normal coverage
Mapping quality
Read position
Trinucleotide
Homopolymer rate
GC content
Genomic location
Bridging reads count
Distance to germline INDEL
Distance to germline SNP
Nucleotide complexity
Strand bias
Read depth analysis
Paired end mapping
Split read alignment
De novo assembly
Aligner
Importance
> 0.05
(0.01, 0.05]
(0.005, 0.01]
(0.001, 0.005]
(0, 0.001]
0
[-0.001, 0)
[-0.005, -0.001)
[-0.01, -0.005)
[-0.05, -0.01)
< -0.05
Algm Feature
Yes
No
Aligner
BWA-backtrack
BWA-MEM
Subread
0.5
0.75
1
Accuracy of model

Figure S26 | Characteristics of prediction errors (continued).

Random Forests assess the importance of 16 sequence-based variables for each team's FN (**a**) and FP (**b**) breakpoints on IS1. Each panel shows variable importance on the left, and algorithmic approaches and aligners used by teams on the right. In the left plot, each row represents the best performing set of predictions by the indicated team, and each column represents the indicated variable. Dot size reflects variable importance, *i.e.* the mean change in accuracy caused by removing the variable from the model (generated to predict erroneous breakpoints). Colour reflects the directional effect of each variable (red and blue for greater and lower variable values, respectively, associated with erroneous breakpoints; black for categorical variables or insignificant directional associations, Mann-Whitney $P > 0.01$). Background shading indicates the accuracy of the model (see colour bar at the bottom). In the right plot, the first four columns indicate usage of the indicated algorithmic approaches by each team and the last column indicates the aligner used. Grey indicates that algorithmic approaches and aligner are unknown for the given team. Abbreviations: Algm, algorithm; SNP, single-nucleotide polymorphism; INDEL, short insertion or deletion.
