# Supplementary material for: Combining accurate tumor genome simulation with crowdsourcing to benchmark somatic structural variant detection
Source: Genome Biol. 2018 Nov 6;19:188. doi: 10.1186/s13059-018-1539-5 (PMC6219177; doi:10.1186/s13059-018-1539-5)
Supplement: Supplementary file 4 — Descriptions of pipelines used to generate submissions. (PDF 3875 kb) [file 13059_2018_1539_MOESM4_ESM.pdf]

ICGC-TCGA DREAM Somatic Mutation Calling Challenge
Submission Documentation Form

Submission name	Subfusion-indel2 (18th, March 2014)
Team	WEHI-Subread
Submission type	☐ INDEL or ☒ SV or ☐ SNV (Only choose one)

1. Read alignment. Did you use the BAM files provided?

☒ Yes ☐ No (Specify your aligner below)

Aligner	Subread	Version	1.4.4
Command lines with parameters	subread-align -r tumor.BAM --BAMinput --reportFusions -i hg19 -o tumor-fusions.sam -m 1 -p 1 -n 28 -d 50 -D 600 -I 5<br>(We used the provided BAM files only for the purpose of retrieving the read sequences. We re-aligned all the reads. The mapping results included in the provided BAM were not used in our analysis.)		

2. Pre-processing steps to calibrate the BAM files. (Command lines and parameters)

N/A

3. Mutation calling algorithm.

Algorithm name	subread	Version	1.4.4

Command lines with parameters	subread-align -r tumor.BAM --BAMinput --reportFusions -i hg19 -o tumor-fusions.sam -m 1 -p 1 -n 28 -d 50 -D 600 -I 5<br>(This command is the same as the command shown above for read alignment. The SVs were called during read alignment and we do not need an extra step to call them.)
Thresholds not indicated by command line	N/A
Reference (check if unpublished ☐)	Liao Y, Smyth GK and Shi W (2013). The Subread aligner: fast, accurate and scalable read mapping by seed-and-vote. *Nucleic Acids Research*, 41(10):e108

4. Post-VCF filtering steps. (Command lines and parameters)

We performed filtering for subread-align output using the following criteria
N >= 10 && (absent in matched normal)
We required each called SV to have at least 10 supporting reads and each called SV should not be present in matched normal sample.
.

5. (Optional) Any other comments or steps not covered.

ICGC-TCGA DREAM Somatic Mutation Calling Challenge
Submission Documentation Form

Submission name	Delly
Team	DellyTeam
Submission type	☐ INDEL or ☒ SV or ☐ SNV (Only choose one)

1. Read alignment. Did you use the BAM files provided?

☒ Yes ☐ No (Specify your aligner below)

Aligner		Version	
Command lines with parameters			

2. Pre-processing steps to calibrate the BAM files. (Command lines and parameters)

None

3. Mutation calling algorithm.

Algorithm name	Delly	Version	IS1: v0.3.2<br>IS2: v0.3.3<br>IS3: v0.5.3
Command lines with parameters	./delly –t DEL –s 9 –o tumor.DEL.pe.vcf –x human.hg19.excl.tsv tumor.bam normal.bam<br>./delly –t DUP –s 9 –o tumor.DUP.pe.vcf –x human.hg19.excl.tsv tumor.bam normal.bam<br>./delly –t INV –s 9 –o tumor.INV.pe.vcf –x human.hg19.excl.tsv tumor.bam normal.bam<br>./delly –t TRA –s 9 –o tumor.TRA.pe.vcf –x human.hg19.excl.tsv tumor.bam normal.bam		

Thresholds not indicated by command line	
Reference (check if unpublished ☐)	Tobias Rausch, Thomas Zichner, Andreas Schlattl, Adrian M. Stuetz, Vladimir Benes, Jan O. Korbel. DELLY: structural variant discovery by integrated paired-end and split-read analysis. Bioinformatics 2012 28: i333-i339.

4. Post-VCF filtering steps. (Command lines and parameters)

Delly calls somatic and germline structural variants. Hence, we post-filtered the raw VCF for somatic variants using a custom python script available in the Delly distribution: https://github.com/tobiasrausch/delly

python ./somaticFilter.py –v tumor.DEL.pe.vcf –o tumor.somatic.DEL.vcf –t DEL –m 400 –a 0.05 –r 0.5 –f
python ./somaticFilter.py –v tumor.DUP.pe.vcf –o tumor.somatic.DUP.vcf –t DUP –m 400 –a 0.05 –r 0.5 –f
python ./somaticFilter.py –v tumor.INV.pe.vcf –o tumor.somatic.INV.vcf –t INV –m 400 –a 0.05 –r 0.5 –f
python ./somaticFilter.py –v tumor.TRA.pe.vcf –o tumor.somatic.TRA.vcf –t TRA –m 0 –a 0.05 –r 0.5 –f

We iteratively used the above commands with different cutoffs for the minimum variant allele frequency '-a' and the minimum SV length '-m'. A subset of low confident calls was manually inspected using a read-depth profile and IGV (http://www.broadinstitute.org/igv/) to define suitable somatic filtering parameters.

5. (Optional) Any other comments or steps not covered.

ICGC-TCGA DREAM Somatic Mutation Calling Challenge
Submission Documentation Form

Submission name	novoBreak
Team	Ken_Chen_Lab
Submission type	☐ INDEL or ☒ SV or ☐ SNV (Only choose one)

1. Read alignment. Did you use the BAM files provided?

☒ Yes ☐ No (Specify your aligner below)

Aligner		Version	
Command lines with parameters			

2. Pre-processing steps to calibrate the BAM files. (Command lines and parameters)

java -jar picard.jar SamToFastq I=synthetic.challenge.set1.tumor.v2.bam F=read1.fq F2=read2.fq
java -jar picard.jar SamToFastq I=synthetic.challenge.set1.normal.v2.bam F=read1.fq F2=read2.fq

3. Mutation calling algorithm.

Algorithm name	novoBreak	Version	v1.02
Command lines with parameters	novobreak $tumor_pair1 $tumor_pair2 $normal_pair1 $normal_pair2 -r $ref -m3 -o kmer.stat -k 31<br>bwa mem -t8 -M $ref somatic_novo_kmer_read1.fq somatic_novo_kmer_read2.fq > somaticreads.sam<br><br>perl group_bp_reads.pl kmer.stat somatic_novo_kmer_read1.fq somatic_novo_kmer_read2.fq somaticreads.sam > bp_reads.txt<br><br>perl run_ssake.pl bp_reads.txt > /dev/null<br><br>bwa mem -t8 -M $ref ssake.fa > ssake.sam<br><br>perl infer_sv.pl ssake.sam > ssake.vcf		

Thresholds not indicated by command line	
Reference (check if unpublished ☒)	Li, Heng. "Aligning sequence reads, clone sequences and assembly contigs with BWA-MEM." arXiv preprint arXiv:1303.3997 (2013). Warren, René L., et al. "Assembling millions of short DNA sequences using SSAKE." Bioinformatics 23.4 (2007): 500-501.

4. Post-VCF filtering steps. (Command lines and parameters)

We used some naive filter, like mapping quality, SV size.

5. (Optional) Any other comments or steps not covered.

ICGC-TCGA DREAM Somatic Mutation Calling Challenge
Submission Documentation Form

Submission name	novoBreak
Team	Ken_Chen_Lab
Submission type	☐ INDEL or ☒ SV or ☐ SNV (Only choose one)

1. Read alignment. Did you use the BAM files provided?

☒ Yes ☐ No (Specify your aligner below)

Aligner		Version	
Command lines with parameters			

2. Pre-processing steps to calibrate the BAM files. (Command lines and parameters)

java -jar picard.jar SamToFastq I=synthetic.challenge.set2.tumor.bam F=read1.fq F2=read2.fq
java -jar picard.jar SamToFastq I=synthetic.challenge.set2.normal.bam F=read1.fq F2=read2.fq

3. Mutation calling algorithm.

Algorithm name	novoBreak	Version	v1.03
Command lines with parameters	novobreak $tumor_pair1 $tumor_pair2 $normal_pair1 $normal_pair2 -r $ref -m3 -o kmer.stat -k 31<br>bwa mem -t8 -M $ref somatic_novo_kmer_read1.fq somatic_novo_kmer_read2.fq > somaticreads.sam<br><br>perl group_bp_reads.pl kmer.stat somatic_novo_kmer_read1.fq somatic_novo_kmer_read2.fq somaticreads.sam > bp_reads.txt<br><br>perl run_ssake.pl bp_reads.txt > /dev/null<br><br>bwa mem -t8 -M $ref ssake.fa > ssake.sam<br><br>perl infer_sv.pl ssake.sam > ssake.vcf		

Thresholds not indicated by command line	
Reference (check if unpublished ☒)	Li, Heng. "Aligning sequence reads, clone sequences and assembly contigs with BWA-MEM." arXiv preprint arXiv:1303.3997 (2013). Warren, René L., et al. "Assembling millions of short DNA sequences using SSAKE." Bioinformatics 23.4 (2007): 500-501.

4. Post-VCF filtering steps. (Command lines and parameters)

```
perl select_pass.pl ssake.vcf > ssake.pass.vcf
perl infer_bp.pl ssake.pass.vcf $tumor_bam $normal_bam > nbasm.pass.sp.vcf
perl filter_sv.pl nbasm.pass.sp.vcf > novoBreak.pass.flt.vcf
```

5. (Optional) Any other comments or steps not covered.

ICGC-TCGA DREAM Somatic Mutation Calling Challenge
Submission Documentation Form

Submission name	novoBreak
Team	Ken_Chen_Lab
Submission type	☐ INDEL or ☒ SV or ☐ SNV (Only choose one)

1. Read alignment. Did you use the BAM files provided?

☒ Yes ☐ No (Specify your aligner below)

Aligner		Version	
Command lines with parameters			

2. Pre-processing steps to calibrate the BAM files. (Command lines and parameters)

java -jar picard.jar SamToFastq I=synthetic.challenge.set3.tumor.bam F=read1.fq F2=read2.fq
java -jar picard.jar SamToFastq I=synthetic.challenge.set3.normal.bam F=read1.fq F2=read2.fq

3. Mutation calling algorithm.

Algorithm name	novoBreak	Version	v1.04

Command lines with parameters

```
novobreak $tumor_pair1 $tumor_pair2 $normal_pair1 $normal_pair2 -r $ref -m3 -o kmer.stat -k 31
 bwa mem -t8 -M $ref somatic_novo_kmer_read1.fq somatic_novo_kmer_read2.fq > somaticreads.sam

perl group_bp_reads.pl kmer.stat somatic_novo_kmer_read1.fq somatic_novo_kmer_read2.fq somaticreads.sam > bp_reads.txt

perl run_ssake.pl bp_reads.txt > /dev/null

bwa mem -t8 -M $ref ssake.fa > ssake.sam

perl infer_sv.pl ssake.sam > ssake.vcf
```

Thresholds not indicated by command line	
Reference (check if unpublished ☒)	Li, Heng. "Aligning sequence reads, clone sequences and assembly contigs with BWA-MEM." arXiv preprint arXiv:1303.3997 (2013). Warren, René L., et al. "Assembling millions of short DNA sequences using SSAKE." Bioinformatics 23.4 (2007): 500-501.

4. Post-VCF filtering steps. (Command lines and parameters)

```
perl select_pass.pl ssake.vcf > ssake.pass.vcf
perl infer_bp.pl ssake.pass.vcf $tumor_bam $normal_bam > nbasm.pass.sp.vcf
perl filter_sv.pl nbasm.pass.sp.vcf > novoBreak.pass.flt.vcf
```

5. (Optional) Any other comments or steps not covered.

ICGC-TCGA DREAM Somatic Mutation Calling Challenge
Submission Documentation Form

Submission name	Manta_sens5 (synthetic_1 ID: 2350297)
Team	MantaStrelka
Submission type	☐ INDEL or ☒ SV or ☐ SNV (Only choose one)

1. Read alignment. Did you use the BAM files provided?

☒ Yes ☐ No (Specify your aligner below)

Aligner		Version	
Command lines with parameters			

2. Pre-processing steps to calibrate the BAM files. (Command lines and parameters)

3. Mutation calling algorithm.

Algorithm name	Manta	Version	v0.18.0-7-ge843cb3
Command lines with parameters	/home/csaunders/devel/manta/install/bin/configManta.py --referenceFasta=/bioinfoSD/smc-dream/ref/Homo_sapiens _assembly19.fasta --normalBam synth_set1/ae16ceb3-ce31-4648-840c-66f3c5d180a6/synthetic.challenge.set1.normal.v2.bam --tumorBam synth_set1/06683320-dbd9-464e-b 6f2-143e715b2981/synthetic.challenge.set1.tumor.v2.bam --runDir=mantaSynthSet1_sens5 && \ /bioinfoSD/users/csaunders/smc-dream/synth1/mantaSynthSet1_sens5/runWorkflow.py -m sge -j 64		

Thresholds not indicated by command line	In the manta global parameter file “configManta.py.ini” the minimum quality scored variant size was changed from the default:<br>minScoredVariantSize = 51<br><br>to:<br>minScoredVariantSize = 100<br><br>…so as to agree with the minimum size variant described in the DREAM simulation data.
Reference (check if unpublished ☐)	Chen X, Schulz-Trieglaff O, Shaw R, Barnes B, Schlesinger F, Källberg M, Cox AJ, Kruglyak S, Saunders CT. Manta: rapid detection of structural variants and indels for germline and cancer sequencing applications. Bioinformatics. 2016;32:1220-2.

4. Post-VCF filtering steps. (Command lines and parameters)

```
# (1) awk filter for PASS’d variants only
#
# (2) inversionFilter.py script condenses inversions from manta’s default
# 1-record per-breakend format to the single-record per inversion
# format presumably expected by the DREAM SCM scoring script.
# This script is also part of v0.18.0-7-ge843cb3 code distribution.
#
gzip –dc ${MANTA_RUN_DIR}/results/variants/somaticSV.vcf.gz |\
awk '/^#/ || ($7=="PASS")' |\
${MANTA_CLONE_DIR}/scratch/util/inversionFilter.py |\
bgzip -c >|\
somaticSV.sens5.pass.vcf.gz
```

5. (Optional) Any other comments or steps not covered.

ICGC-TCGA DREAM Somatic Mutation Calling Challenge
Submission Documentation Form

Submission name	Manta_v20b (synthetic_2 ID: 2385728)
Team	MantaStrelka

Submission type	☐ INDEL or ☒ SV or ☐ SNV (Only choose one)

1. Read alignment. Did you use the BAM files provided?

☒ Yes ☐ No (Specify your aligner below)

Aligner		Version	

Command lines with parameters	

2. Pre-processing steps to calibrate the BAM files. (Command lines and parameters)

3. Mutation calling algorithm.

Algorithm name	Manta	Version	v0.19.0-63-ga22d8e2

Command lines with parameter	/home/csaunders/devel/manta-135/install/bin/configManta.py --referenceFasta /illumina/scratch/TUNE/data/references/broad_assembly19/Homo_sapiens_assembly19.fasta --normalBam /illumina/scratch/TUNE/data/DREAM_SMC/synth2/orig_bams/865fa3d6-2024-47cf-bf66-c258f8c0efcf/synthetic.challenge.set2.normal.bam --tumorBam /illumina/scratch/TUNE/data/DREAM_SMC/synth2/orig_bams/7cf8416e-6055-4a0e-86ee-b719db9fbc16/synthetic.challenge.set2.tumor.bam --runDir=/illumina/scratch/TUNE/shortterm/manta_test/m135_test/synth2/m135_synth2_v5 && \ /illumina/scratch/TUNE/shortterm/manta_test/m135_test/synth2/m135_synth2_v5/runWorkflow.py -m local -j 12
Thresholds not indicated by command line	In the manta global parameter file “configManta.py.ini” the minimum quality scored variant size was changed from the default:<br>minScoredVariantSize = 51<br><br>to:<br>minScoredVariantSize = 100<br><br>…so as to agree with the minimum size variant described in the DREAM simulation data.
Reference (check if unpublished ☐)	Chen X, Schulz-Trieglaff O, Shaw R, Barnes B, Schlesinger F, Källberg M, Cox AJ, Kruglyak S, Saunders CT. Manta: rapid detection of structural variants and indels for germline and cancer sequencing applications. Bioinformatics. 2016;32:1220-2.

4. Post-VCF filtering steps. (Command lines and parameters)

```
# (1) awk filter for PASS'd variants only
#
# (2) inversionFilter.py script condenses inversions from manta's default
# 1-record per-breakend format to the single-record per inversion
# format presumably expected by the DREAM SCM scoring script.
#
# (3) largeIntrachromFilter.py script removes all intra-chromosomal translocations above
# 100kb. This step was added in response to the DREAM SCM scoring script which
# significantly penalized manta results in IS1 as the result of a single large intra-chromosomal
# call.
#
# (4) overlapFilter.py script removes the larger of any overlapping PASS'd SV calls (except
# translocations)
#
```

```
# Scripts (2), (3) and (4) are also part of v0.19.0-63-ga22d8e2 code distribution.

script_dir=${MANTA_CLONE_DIR}/scratch/util
gzip –dc ${MANTA_RUN_DIR}/results/variants/somaticSV.vcf.gz |\
awk '/^#/ || /PASS/' |\
$script_dir/inversionFilter.py |\
$script_dir/largeIntrachromFilter.py --maxSize 100000 |\
$script_dir/overlapFilter.py |\
bgzip -c >|\
somaticSV.c.filter2.vcf.gz
```

5. (Optional) Any other comments or steps not covered.

ICGC-TCGA DREAM Somatic Mutation Calling Challenge
Submission Documentation Form

Submission name	Manta_sv (synthetic_3 ID: 2478162)
Team	MantaStrelka

Submission type	☐ INDEL or ☒ SV or ☐ SNV (Only choose one)

1. Read alignment. Did you use the BAM files provided?

☒ Yes ☐ No (Specify your aligner below)

Aligner		Version	

Command lines with parameters	

2. Pre-processing steps to calibrate the BAM files. (Command lines and parameters)

3. Mutation calling algorithm.

Algorithm name	Manta	Version	v0.21.0-63-g892f0c5

Command lines with parameters	/home/csaunders/devel/manta-169/install2/bin/configManta.py --referenceFasta /illumina/scratch/TUNE/data/references/broad_assembly19/Homo_sapiens_assembly19.fasta --normalBam /illumina/scratch/TUNE/data/DREAM_SMC/synth3/orig_bams/b19d76a0-a487-4c50-8f9c-3b4d5e53239d/synthetic.challenge.set3.normal.bam --tumorBam /illumina/scratch/TUNE/data/DREAM_SMC/synth3/orig_bams/8fe6fc33-2daf-4393-929f-7c3493d04bef/synthetic.challenge.set3.tumor.bam --runDir=m169_synth3_both_v7 && \ /illumina/scratch/TUNE/shortterm/manta_test/m168_dream_test/synth3/m169_synth3_both_v7/runWorkflow.py -m sge -j 64
Thresholds not indicated by command line	In the manta global parameter file "configManta.py.ini" the minimum quality scored variant size was changed from the default:<br>minScoredVariantSize = 51<br><br>to:<br>minScoredVariantSize = 100<br><br>...so as to agree with the minimum size variant described in the DREAM simulation data.
Reference (check if unpublished ☐)	Chen X, Schulz-Trieglaff O, Shaw R, Barnes B, Schlesinger F, Källberg M, Cox AJ, Kruglyak S, Saunders CT. Manta: rapid detection of structural variants and indels for germline and cancer sequencing applications. Bioinformatics. 2016;32:1220-2.

4. Post-VCF filtering steps. (Command lines and parameters)

```
# (1) reFilterVcf.py script changes default filtration level to filter calls with SOMATICSCORE less than 10 (default is less than 30)
#
# (2) minSVSizeFilter.py script removes SV/indel calls of size 100 or less
#
# (3) awk filter for PASS'd variants only
#
# (4) inversionFilter.py script condenses inversions from manta's default
# 1-record per-breakend format to the single-record per inversion
# format presumably expected by the DREAM SCM scoring script.
#
# (5) largeIntrachromFilter.py script removes all intra-chromosomal translocations above
# 100kb. This step was added in response to the DREAM SCM scoring script which
```

```
# significantly penalized manta results in IS1 as the result of a single large intra-chromosomal
# call.
#
# (6) overlapFilter.py script removes the larger of any overlapping PASS'd SV calls (except
# translocations)
#
# (7) pairSupportFilter.py script removes calls with only one supporting paired read in the
normal sample when SOMATICSCORE is less than or equal to 20
#
# All scripts are also part of v0.21.0-63-g892f0c5 code distribution.

script_dir=${MANTA_CLONE_DIR}/scratch/util
gzip –dc ${MANTA_RUN_DIR}/results/variants/somaticSV.vcf.gz |\
$script_dir/reFilterVcf.py --minSS 10 |\
$script_dir/minSVSizeFilter.py --minSize 100 |\
awk '/^#/ || /PASS/' |\
$script_dir/inversionFilter.py |\
$script_dir/largeIntrachromFilter.py --maxSize 100000 |\
$script_dir/overlapFilter.py |\
$script_dir/pairSupportFilter.py |\
bgzip -c >|\
v7_somaticSV.gt100.filtered.q10.vcf.gz
```

5. (Optional) Any other comments or steps not covered.

ICGC-TCGA DREAM Somatic Mutation Calling Challenge
Submission Documentation Form

Submission name	GROMX1 (2385706, synthetic challenge 2)
Team	Grigoriev-lab
Submission type	☐ INDEL or ☒ SV or ☐ SNV (Only choose one)

1. Read alignment. Did you use the BAM files provided?

☐ Yes ☒ No (Specify your aligner below)

Aligner	BWA	Version	0.7.4
Command lines with parameters	bwa mem -t 32 ./hg19.fa ./synthetic.challenge.set2.normal.1.fastq ./synthetic.challenge.set2.normal.2.fastq > synthetic.challenge.set2.normal.bwa_mem.sam		

2. Pre-processing steps to calibrate the BAM files. (Command lines and parameters)

1) samtools view -bS -h synthetic.challenge.set2.normal.bwa_mem.sam > synthetic.challenge.set2.normal.bwa_mem.bam
2) samtools sort synthetic.challenge.set2.normal.bwa_mem.bam synthetic.challenge.set2.normal.bwa_mem _sorted
3) samtools index synthetic.challenge.set2.normal.bwa_mem _sorted.bam
4) samtools rmdup synthetic.challenge.set2.normal.bwa_mem _sorted.bam synthetic.challenge.set2.normal.bwa_mem _sorted _rmdup.bam
5) samtools index synthetic.challenge.set2.normal.bwa_mem _sorted _rmdup.bam

3. Mutation calling algorithm.

Algorithm name	GROM	Version	0.0.83

Command lines with parameters	python GROM.py -i synthetic.challenge.set2.normal.bwa_mem _sorted _rmdup.bam -r hg19.fa -o synthetic2_normal_GROM_0_0_83_q35_bwa_mem.txt -p 2 -q 35<br><br>python GROM.py -i synthetic.challenge.set2.tumor.bwa_mem _sorted _rmdup.bam -r hg19.fa -o synthetic2_tumor_GROM_0_0_83_q35_bwa_mem.txt -p 2 -q 35
Thresholds not indicated by command line	
Reference (check if unpublished ☐)	Smith SD, Kawash JK, Grigoriev A. Lightning-fast genome variant detection with GROM. GigaScience. 2017;6:1-7. https://doi.org/10.1093/gigascience/gix091

4. Post-VCF filtering steps. (Command lines and parameters)

python vcf_parser20.py -d 35 -u 18 -i 13 -n synthetic2_normal_GROM_0_0_83_q35_bwa_mem.txt -t synthetic2_tumor_GROM_0_0_83_q35_bwa_mem.txt

5. (Optional) Any other comments or steps not covered.

For vcf_parser (4. Post-VCF filtering steps), -d -u -i are thresholds for deletions, duplications, and inversions, respectively, where a higher value is more stringent.

ICGC-TCGA DREAM Somatic Mutation Calling Challenge
Submission Documentation Form

Submission name	GROMX1A (2385713, synthetic challenge 2)
Team	Grigoriev-lab
Submission type	☐ INDEL or ☒ SV or ☐ SNV (Only choose one)

1. Read alignment. Did you use the BAM files provided?

☐ Yes ☒ No (Specify your aligner below)

Aligner	BWA	Version	0.7.4
Command lines with parameters	bwa mem -t 32 ./hg19.fa ./synthetic.challenge.set2.normal.1.fastq ./ synthetic.challenge.set2.normal.2.fastq > synthetic.challenge.set2.normal.bwa_mem.sam		

2. Pre-processing steps to calibrate the BAM files. (Command lines and parameters)

1) samtools view -bS -h synthetic.challenge.set2.normal.bwa_mem.sam > synthetic.challenge.set2.normal.bwa_mem.bam
2) samtools sort synthetic.challenge.set2.normal.bwa_mem.bam synthetic.challenge.set2.normal.bwa_mem _sorted
3) samtools index synthetic.challenge.set2.normal.bwa_mem _sorted.bam
4) samtools rmdup synthetic.challenge.set2.normal.bwa_mem _sorted.bam synthetic.challenge.set2.normal.bwa_mem _sorted _rmdup.bam
5) samtools index synthetic.challenge.set2.normal.bwa_mem _sorted _rmdup.bam

3. Mutation calling algorithm.

Algorithm name	GROM	Version	0.0.83

Command lines with parameters	python GROM.py -i synthetic.challenge.set2.normal.bwa_mem _sorted _rmdup.bam -r hg19.fa -o synthetic2_normal_GROM_0_0_83_q35_bwa_mem.txt -p 2 -q 35 -e<br><br>python GROM.py -i synthetic.challenge.set2.tumor.bwa_mem _sorted _rmdup.bam -r hg19.fa -o synthetic2_tumor_GROM_0_0_83_q35_bwa_mem.txt -p 2 -q 35 -e
Thresholds not indicated by command line	
Reference (check if unpublished ☐)	Smith SD, Kawash JK, Grigoriev A. Lightning-fast genome variant detection with GROM. GigaScience. 2017;6:1-7. https://doi.org/10.1093/gigascience/gix091

4. Post-VCF filtering steps. (Command lines and parameters)

python vcf_parser20.py -d 35 -u 18 -i 13 -n synthetic2_normal_GROM_0_0_83_q35_bwa_mem.txt -t synthetic2_tumor_GROM_0_0_83_q35_bwa_mem.txt

5. (Optional) Any other comments or steps not covered.

For vcf_parser (4. Post-VCF filtering steps), -d -u -i are thresholds for deletions, duplications, and inversions, respectively, where a higher value is more stringent.

ICGC-TCGA DREAM Somatic Mutation Calling Challenge
Submission Documentation Form

Submission name	GROMX2 (2385707, synthetic challenge 2)
Team	Grigoriev-lab
Submission type	☐ INDEL or ☒ SV or ☐ SNV (Only choose one)

1. Read alignment. Did you use the BAM files provided?

☐ Yes ☒ No (Specify your aligner below)

Aligner	BWA	Version	0.7.4
Command lines with parameters	bwa mem -t 32 ./hg19.fa ./synthetic.challenge.set2.normal.1.fastq ./synthetic.challenge.set2.normal.2.fastq > synthetic.challenge.set2.normal.bwa_mem.sam		

2. Pre-processing steps to calibrate the BAM files. (Command lines and parameters)

1) samtools view -bS -h synthetic.challenge.set2.normal.bwa_mem.sam > synthetic.challenge.set2.normal.bwa_mem.bam
2) samtools sort synthetic.challenge.set2.normal.bwa_mem.bam synthetic.challenge.set2.normal.bwa_mem _sorted
3) samtools index synthetic.challenge.set2.normal.bwa_mem _sorted.bam
4) samtools rmdup synthetic.challenge.set2.normal.bwa_mem _sorted.bam synthetic.challenge.set2.normal.bwa_mem _sorted _rmdup.bam
5) samtools index synthetic.challenge.set2.normal.bwa_mem _sorted _rmdup.bam

3. Mutation calling algorithm.

Algorithm name	GROM	Version	0.0.83

Command lines with parameters	python GROM.py -i synthetic.challenge.set2.normal.bwa_mem _sorted _rmdup.bam -r hg19.fa -o synthetic2_normal_GROM_0_0_83_q35_bwa_mem.txt -p 2 -q 35<br><br>python GROM.py -i synthetic.challenge.set2.tumor.bwa_mem _sorted _rmdup.bam -r hg19.fa -o synthetic2_tumor_GROM_0_0_83_q35_bwa_mem.txt -p 2 -q 35
Thresholds not indicated by command line	
Reference (check if unpublished ☐)	Smith SD, Kawash JK, Grigoriev A. Lightning-fast genome variant detection with GROM. GigaScience. 2017;6:1-7. https://doi.org/10.1093/gigascience/gix091

4. Post-VCF filtering steps. (Command lines and parameters)

python vcf_parser20.py -d 41 -u 23 -i 15 -n synthetic2_normal_GROM_0_0_83_q35_bwa_mem.txt -t synthetic2_tumor_GROM_0_0_83_q35_bwa_mem.txt

5. (Optional) Any other comments or steps not covered.

For vcf_parser (4. Post-VCF filtering steps), -d -u -i are thresholds for deletions, duplications, and inversions, respectively, where a higher value is more stringent.

ICGC-TCGA DREAM Somatic Mutation Calling Challenge
Submission Documentation Form

Submission name	GROMX3 (2385708, synthetic challenge 2)
Team	Grigoriev-lab
Submission type	☐ INDEL or ☒ SV or ☐ SNV (Only choose one)

1. Read alignment. Did you use the BAM files provided?

☐ Yes ☒ No (Specify your aligner below)

Aligner	BWA	Version	0.7.4
Command lines with parameters	bwa mem -t 32 ./hg19.fa ./synthetic.challenge.set2.normal.1.fastq ./synthetic.challenge.set2.normal.2.fastq > synthetic.challenge.set2.normal.bwa_mem.sam		

2. Pre-processing steps to calibrate the BAM files. (Command lines and parameters)

1) samtools view -bS -h synthetic.challenge.set2.normal.bwa_mem.sam > synthetic.challenge.set2.normal.bwa_mem.bam
2) samtools sort synthetic.challenge.set2.normal.bwa_mem.bam synthetic.challenge.set2.normal.bwa_mem _sorted
3) samtools index synthetic.challenge.set2.normal.bwa_mem _sorted.bam
4) samtools rmdup synthetic.challenge.set2.normal.bwa_mem _sorted.bam synthetic.challenge.set2.normal.bwa_mem _sorted _rmdup.bam
5) samtools index synthetic.challenge.set2.normal.bwa_mem _sorted _rmdup.bam

3. Mutation calling algorithm.

Algorithm name	GROM	Version	0.0.83

Command lines with parameters	python GROM.py -i synthetic.challenge.set2.normal.bwa_mem _sorted _rmdup.bam -r hg19.fa -o synthetic2_normal_GROM_0_0_83_q35_bwa_mem.txt -p 2 -q 35<br><br>python GROM.py -i synthetic.challenge.set2.tumor.bwa_mem _sorted _rmdup.bam -r hg19.fa -o synthetic2_tumor_GROM_0_0_83_q35_bwa_mem.txt -p 2 -q 35
Thresholds not indicated by command line	
Reference (check if unpublished ☐)	Smith SD, Kawash JK, Grigoriev A. Lightning-fast genome variant detection with GROM. GigaScience. 2017;6:1-7. https://doi.org/10.1093/gigascience/gix091

4. Post-VCF filtering steps. (Command lines and parameters)

python vcf_parser20.py -d 42 -u 24 -i 16 -n synthetic2_normal_GROM_0_0_83_q35_bwa_mem.txt -t synthetic2_tumor_GROM_0_0_83_q35_bwa_mem.txt

5. (Optional) Any other comments or steps not covered.

For vcf_parser (4. Post-VCF filtering steps), -d -u -i are thresholds for deletions, duplications, and inversions, respectively, where a higher value is more stringent.

ICGC-TCGA DREAM Somatic Mutation Calling Challenge
Submission Documentation Form

Submission name	GROMX4 (2385709, synthetic challenge 2)
Team	Grigoriev-lab
Submission type	☐ INDEL or ☒ SV or ☐ SNV (Only choose one)

1. Read alignment. Did you use the BAM files provided?

☐ Yes ☒ No (Specify your aligner below)

Aligner	BWA	Version	0.7.4
Command lines with parameters	bwa mem -t 32 ./hg19.fa ./synthetic.challenge.set2.normal.1.fastq ./synthetic.challenge.set2.normal.2.fastq > synthetic.challenge.set2.normal.bwa_mem.sam		

2. Pre-processing steps to calibrate the BAM files. (Command lines and parameters)

1) samtools view -bS -h synthetic.challenge.set2.normal.bwa_mem.sam > synthetic.challenge.set2.normal.bwa_mem.bam
2) samtools sort synthetic.challenge.set2.normal.bwa_mem.bam synthetic.challenge.set2.normal.bwa_mem _sorted
3) samtools index synthetic.challenge.set2.normal.bwa_mem _sorted.bam
4) samtools rmdup synthetic.challenge.set2.normal.bwa_mem _sorted.bam synthetic.challenge.set2.normal.bwa_mem _sorted _rmdup.bam
5) samtools index synthetic.challenge.set2.normal.bwa_mem _sorted _rmdup.bam

3. Mutation calling algorithm.

Algorithm name	GROM	Version	0.0.83

Command lines with parameters	python GROM.py -i synthetic.challenge.set2.normal.bwa_mem _sorted _rmdup.bam -r hg19.fa -o synthetic2_normal_GROM_0_0_83_q35_bwa_mem.txt -p 2 -q 35<br><br>python GROM.py -i synthetic.challenge.set2.tumor.bwa_mem _sorted _rmdup.bam -r hg19.fa -o synthetic2_tumor_GROM_0_0_83_q35_bwa_mem.txt -p 2 -q 35
Thresholds not indicated by command line	
Reference (check if unpublished ☐)	Smith SD, Kawash JK, Grigoriev A. Lightning-fast genome variant detection with GROM. GigaScience. 2017;6:1-7. https://doi.org/10.1093/gigascience/gix091

4. Post-VCF filtering steps. (Command lines and parameters)

python vcf_parser20.py -d 45 -u 28 -i 18 -n synthetic2_normal_GROM_0_0_83_q35_bwa_mem.txt -t synthetic2_tumor_GROM_0_0_83_q35_bwa_mem.txt

5. (Optional) Any other comments or steps not covered.

For vcf_parser (4. Post-VCF filtering steps), -d -u -i are thresholds for deletions, duplications, and inversions, respectively, where a higher value is more stringent.

ICGC-TCGA DREAM Somatic Mutation Calling Challenge
Submission Documentation Form

Submission name	Syn3_cv1 (2475738, synthetic challenge 3)
Team	Grigoriev-lab
Submission type	☐ INDEL or ☒ SV or ☐ SNV (Only choose one)

1. Read alignment. Did you use the BAM files provided?

☐ Yes ☒ No (Specify your aligner below)

Aligner	BWA	Version	0.7.4
Command lines with parameters	bwa mem -t 32 ./hg19.fa ./synthetic.challenge.set3.normal.1.fastq ./ synthetic.challenge.set3.normal.2.fastq > synthetic.challenge.set3.normal.bwa_mem.sam		

2. Pre-processing steps to calibrate the BAM files. (Command lines and parameters)

1) samtools view -bS -h synthetic.challenge.set3.normal.bwa_mem.sam > synthetic.challenge.set3.normal.bwa_mem.bam
2) samtools sort synthetic.challenge.set3.normal.bwa_mem.bam synthetic.challenge.set3.normal.bwa_mem _sorted
3) samtools index synthetic.challenge.set3.normal.bwa_mem _sorted.bam
4) samtools rmdup synthetic.challenge.set3.normal.bwa_mem _sorted.bam synthetic.challenge.set3.normal.bwa_mem _sorted _rmdup.bam
5) samtools index synthetic.challenge.set3.normal.bwa_mem _sorted _rmdup.bam

3. Mutation calling algorithm.

Algorithm name	GROM	Version	0.0.85

Command lines with parameters	GROM -i synthetic.challenge.set3.normal.bwa_mem _sorted _rmdup.bam -r hg19.fa -o synthetic3_normal_GROM_0_0_85_q0_bwa_mem.txt -p 2 -q 0<br><br>GROM -i synthetic.challenge.set3.tumor.bwa_mem _sorted _rmdup.bam -r hg19.fa -o synthetic3_tumor_GROM_0_0_85_q35_bwa_mem.txt -p 2 -q 35
Thresholds not indicated by command line	
Reference (check if unpublished ☐)	Smith SD, Kawash JK, Grigoriev A. Lightning-fast genome variant detection with GROM. GigaScience. 2017;6:1-7. https://doi.org/10.1093/gigascience/gix091

4. Post-VCF filtering steps. (Command lines and parameters)

python vcf_parser32.py -l -g 50-d 17 -u 11 -i 22 -s 27 -n synthetic3_normal_GROM_0_0_85_q0_bwa_mem.txt -t synthetic3_tumor_GROM_0_0_85_q35_bwa_mem.txt

5. (Optional) Any other comments or steps not covered.

For vcf_parser (4. Post-VCF filtering steps), -d -u -i -s are thresholds for deletions, duplications, inversions, and insertions, respectively, where a higher value is more stringent.

ICGC-TCGA DREAM Somatic Mutation Calling Challenge
Submission Documentation Form

Submission name	Syn3_V34c_S1 (2476543, synthetic challenge 3)
Team	Grigoriev-lab
Submission type	☐ INDEL or ☒ SV or ☐ SNV (Only choose one)

1. Read alignment. Did you use the BAM files provided?

☐ Yes ☒ No (Specify your aligner below)

Aligner	BWA	Version	0.7.4
Command lines with parameters	bwa mem -t 32 ./hg19.fa ./synthetic.challenge.set3.normal.1.fastq ./synthetic.challenge.set3.normal.2.fastq > synthetic.challenge.set3.normal.bwa_mem.sam		

2. Pre-processing steps to calibrate the BAM files. (Command lines and parameters)

1) samtools view -bS -h synthetic.challenge.set3.normal.bwa_mem.sam > synthetic.challenge.set3.normal.bwa_mem.bam
2) samtools sort synthetic.challenge.set3.normal.bwa_mem.bam synthetic.challenge.set3.normal.bwa_mem _sorted
3) samtools index synthetic.challenge.set3.normal.bwa_mem _sorted.bam
4) samtools rmdup synthetic.challenge.set3.normal.bwa_mem _sorted.bam synthetic.challenge.set3.normal.bwa_mem _sorted _rmdup.bam
5) samtools index synthetic.challenge.set3.normal.bwa_mem _sorted _rmdup.bam

3. Mutation calling algorithm.

Algorithm name	GROM	Version	0.0.85

Command lines with parameters	GROM -i synthetic.challenge.set3.normal.bwa_mem _sorted _rmdup.bam -r hg19.fa -o synthetic3_normal_GROM_0_0_85_q0_bwa_mem.txt -p 2 -q 0<br><br>GROM -i synthetic.challenge.set3.tumor.bwa_mem _sorted _rmdup.bam -r hg19.fa -o synthetic3_tumor_GROM_0_0_85_q35_bwa_mem.txt -p 2 -q 35
Thresholds not indicated by command line	
Reference (check if unpublished ☐)	Smith SD, Kawash JK, Grigoriev A. Lightning-fast genome variant detection with GROM. GigaScience. 2017;6:1-7. https://doi.org/10.1093/gigascience/gix091

4. Post-VCF filtering steps. (Command lines and parameters)

python vcf_parser34.py -l -c -d 18 -u 11 -i 21 -s 26 -n synthetic3_normal_GROM_0_0_85_q0_bwa_mem.txt -t synthetic3_tumor_GROM_0_0_85_q35_bwa_mem.txt

5. (Optional) Any other comments or steps not covered.

For vcf_parser (4. Post-VCF filtering steps), -d -u -i -s are thresholds for deletions, duplications, inversions, and insertions, respectively, where a higher value is more stringent.

ICGC-TCGA DREAM Somatic Mutation Calling Challenge
Submission Documentation Form

Submission name	Syn3_V34c_S2 (2476561, synthetic challenge 3)
Team	Grigoriev-lab
Submission type	☐ INDEL or ☒ SV or ☐ SNV (Only choose one)

1. Read alignment. Did you use the BAM files provided?

☐ Yes ☒ No (Specify your aligner below)

Aligner	BWA	Version	0.7.4
Command lines with parameters	bwa mem -t 32 ./hg19.fa ./synthetic.challenge.set3.normal.1.fastq ./synthetic.challenge.set3.normal.2.fastq > synthetic.challenge.set3.normal.bwa_mem.sam		

2. Pre-processing steps to calibrate the BAM files. (Command lines and parameters)

1) samtools view -bS -h synthetic.challenge.set3.normal.bwa_mem.sam > synthetic.challenge.set3.normal.bwa_mem.bam
2) samtools sort synthetic.challenge.set3.normal.bwa_mem.bam synthetic.challenge.set3.normal.bwa_mem _sorted
3) samtools index synthetic.challenge.set3.normal.bwa_mem _sorted.bam
4) samtools rmdup synthetic.challenge.set3.normal.bwa_mem _sorted.bam synthetic.challenge.set3.normal.bwa_mem _sorted _rmdup.bam
5) samtools index synthetic.challenge.set3.normal.bwa_mem _sorted _rmdup.bam

3. Mutation calling algorithm.

Algorithm name	GROM	Version	0.0.85

Command lines with parameters	GROM -i synthetic.challenge.set3.normal.bwa_mem _sorted _rmdup.bam -r hg19.fa -o synthetic3_normal_GROM_0_0_85_q0_bwa_mem.txt -p 2 -q 0<br><br>GROM -i synthetic.challenge.set3.tumor.bwa_mem _sorted _rmdup.bam -r hg19.fa -o synthetic3_tumor_GROM_0_0_85_q35_bwa_mem.txt -p 2 -q 35
Thresholds not indicated by command line	
Reference (check if unpublished ☐)	Smith SD, Kawash JK, Grigoriev A. Lightning-fast genome variant detection with GROM. GigaScience. 2017;6:1-7. https://doi.org/10.1093/gigascience/gix091

4. Post-VCF filtering steps. (Command lines and parameters)

```
python vcf_parser34.py -l -c -d 13 -u 7 -i 16 -s 12 -n
synthetic3_normal_GROM_0_0_85_q0_bwa_mem.txt -t
synthetic3_tumor_GROM_0_0_85_q35_bwa_mem.txt
```

5. (Optional) Any other comments or steps not covered.

For vcf_parser (4. Post-VCF filtering steps), -d -u -i -s are thresholds for deletions, duplications, inversions, and insertions, respectively, where a higher value is more stringent.

ICGC-TCGA DREAM Somatic Mutation Calling Challenge
Submission Documentation Form

Submission name	Syn3_V34c_S3 (2476579, synthetic challenge 3)
Team	Grigoriev-lab
Submission type	☐ INDEL or ☒ SV or ☐ SNV (Only choose one)

1. Read alignment. Did you use the BAM files provided?

☐ Yes ☒ No (Specify your aligner below)

Aligner	BWA	Version	0.7.4
Command lines with parameters	bwa mem -t 32 ./hg19.fa ./synthetic.challenge.set3.normal.1.fastq ./synthetic.challenge.set3.normal.2.fastq > synthetic.challenge.set3.normal.bwa_mem.sam		

2. Pre-processing steps to calibrate the BAM files. (Command lines and parameters)

1) samtools view -bS -h synthetic.challenge.set3.normal.bwa_mem.sam > synthetic.challenge.set3.normal.bwa_mem.bam
2) samtools sort synthetic.challenge.set3.normal.bwa_mem.bam synthetic.challenge.set3.normal.bwa_mem _sorted
3) samtools index synthetic.challenge.set3.normal.bwa_mem _sorted.bam
4) samtools rmdup synthetic.challenge.set3.normal.bwa_mem _sorted.bam synthetic.challenge.set3.normal.bwa_mem _sorted _rmdup.bam
5) samtools index synthetic.challenge.set3.normal.bwa_mem _sorted _rmdup.bam

3. Mutation calling algorithm.

Algorithm name	GROM	Version	0.0.85

Command lines with parameters	GROM -i synthetic.challenge.set3.normal.bwa_mem _sorted _rmdup.bam -r hg19.fa -o synthetic3_normal_GROM_0_0_85_q0_bwa_mem.txt -p 2 -q 0<br><br>GROM -i synthetic.challenge.set3.tumor.bwa_mem _sorted _rmdup.bam -r hg19.fa -o synthetic3_tumor_GROM_0_0_85_q35_bwa_mem.txt -p 2 -q 35
Thresholds not indicated by command line	
Reference (check if unpublished ☐)	Smith SD, Kawash JK, Grigoriev A. Lightning-fast genome variant detection with GROM. GigaScience. 2017;6:1-7. https://doi.org/10.1093/gigascience/gix091

4. Post-VCF filtering steps. (Command lines and parameters)

python vcf_parser34.py -l -c -d 24 -u 17 -i 26 -s 30 -n synthetic3_normal_GROM_0_0_85_q0_bwa_mem.txt -t synthetic3_tumor_GROM_0_0_85_q35_bwa_mem.txt

5. (Optional) Any other comments or steps not covered.

For vcf_parser (4. Post-VCF filtering steps), -d -u -i -s are thresholds for deletions, duplications, inversions, and insertions, respectively, where a higher value is more stringent.

ICGC-TCGA DREAM Somatic Mutation Calling Challenge
Submission Documentation Form

Submission name	Syn3_V37c_S2 (2478182, synthetic challenge 3)
Team	Grigoriev-lab
Submission type	☐ INDEL or ☒ SV or ☐ SNV (Only choose one)

1. Read alignment. Did you use the BAM files provided?

☐ Yes ☒ No (Specify your aligner below)

Aligner	BWA	Version	0.7.4
Command lines with parameters	bwa mem -t 32 ./hg19.fa ./synthetic.challenge.set3.normal.1.fastq ./synthetic.challenge.set3.normal.2.fastq > synthetic.challenge.set3.normal.bwa_mem.sam		

2. Pre-processing steps to calibrate the BAM files. (Command lines and parameters)

1) samtools view -bS -h synthetic.challenge.set3.normal.bwa_mem.sam > synthetic.challenge.set3.normal.bwa_mem.bam
2) samtools sort synthetic.challenge.set3.normal.bwa_mem.bam synthetic.challenge.set3.normal.bwa_mem _sorted
3) samtools index synthetic.challenge.set3.normal.bwa_mem _sorted.bam
4) samtools rmdup synthetic.challenge.set3.normal.bwa_mem _sorted.bam synthetic.challenge.set3.normal.bwa_mem _sorted _rmdup.bam
5) samtools index synthetic.challenge.set3.normal.bwa_mem _sorted _rmdup.bam

3. Mutation calling algorithm.

Algorithm name	GROM	Version	0.0.85

Command lines with parameters	GROM -i synthetic.challenge.set3.normal.bwa_mem _sorted _rmdup.bam -r hg19.fa -o synthetic3_normal_GROM_0_0_85_q0_bwa_mem.txt -p 2 -q 0<br><br>GROM -i synthetic.challenge.set3.tumor.bwa_mem _sorted _rmdup.bam -r hg19.fa -o synthetic3_tumor_GROM_0_0_85_q35_bwa_mem.txt -p 2 -q 35
Thresholds not indicated by command line	
Reference (check if unpublished ☐)	Smith SD, Kawash JK, Grigoriev A. Lightning-fast genome variant detection with GROM. GigaScience. 2017;6:1-7. https://doi.org/10.1093/gigascience/gix091

4. Post-VCF filtering steps. (Command lines and parameters)

python vcf_parser37.py -l -c -d 17 -u 12 -i 30 -s 12 -n synthetic3_normal_GROM_0_0_85_q0_bwa_mem.txt -t synthetic3_tumor_GROM_0_0_85_q35_bwa_mem.txt

5. (Optional) Any other comments or steps not covered.

For vcf_parser (4. Post-VCF filtering steps), -d -u -i -s are thresholds for deletions, duplications, inversions, and insertions, respectively, where a higher value is more stringent.

ICGC-TCGA DREAM Somatic Mutation Calling Challenge
Submission Documentation Form

Submission name	Syn3_V37c_S2.5 (2478148, synthetic challenge 3)
Team	Grigoriev-lab
Submission type	☐ INDEL or ☒ SV or ☐ SNV (Only choose one)

1. Read alignment. Did you use the BAM files provided?

☐ Yes ☒ No (Specify your aligner below)

Aligner	BWA	Version	0.7.4
Command lines with parameters	bwa mem -t 32 ./hg19.fa ./synthetic.challenge.set3.normal.1.fastq ./synthetic.challenge.set3.normal.2.fastq > synthetic.challenge.set3.normal.bwa_mem.sam		

2. Pre-processing steps to calibrate the BAM files. (Command lines and parameters)

1) samtools view -bS -h synthetic.challenge.set3.normal.bwa_mem.sam > synthetic.challenge.set3.normal.bwa_mem.bam
2) samtools sort synthetic.challenge.set3.normal.bwa_mem.bam synthetic.challenge.set3.normal.bwa_mem _sorted
3) samtools index synthetic.challenge.set3.normal.bwa_mem _sorted.bam
4) samtools rmdup synthetic.challenge.set3.normal.bwa_mem _sorted.bam synthetic.challenge.set3.normal.bwa_mem _sorted _rmdup.bam
5) samtools index synthetic.challenge.set3.normal.bwa_mem _sorted _rmdup.bam

3. Mutation calling algorithm.

Algorithm name	GROM	Version	0.0.85

Command lines with parameters	GROM -i synthetic.challenge.set3.normal.bwa_mem _sorted _rmdup.bam -r hg19.fa -o synthetic3_normal_GROM_0_0_85_q0_bwa_mem.txt -p 2 -q 0<br><br>GROM -i synthetic.challenge.set3.tumor.bwa_mem _sorted _rmdup.bam -r hg19.fa -o synthetic3_tumor_GROM_0_0_85_q35_bwa_mem.txt -p 2 -q 35
Thresholds not indicated by command line	
Reference (check if unpublished ☐)	Smith SD, Kawash JK, Grigoriev A. Lightning-fast genome variant detection with GROM. GigaScience. 2017;6:1-7. https://doi.org/10.1093/gigascience/gix091

4. Post-VCF filtering steps. (Command lines and parameters)

python vcf_parser37.py -l -c -d 15 -u 14 -i 34 -s 21 -n synthetic3_normal_GROM_0_0_85_q0_bwa_mem.txt -t synthetic3_tumor_GROM_0_0_85_q35_bwa_mem.txt

5. (Optional) Any other comments or steps not covered.

For vcf_parser (4. Post-VCF filtering steps), -d -u -i -s are thresholds for deletions, duplications, inversions, and insertions, respectively, where a higher value is more stringent.

ICGC-TCGA DREAM Somatic Mutation Calling Challenge
Submission Documentation Form

Submission name	Syn3_V37c_S3 (2478175, synthetic challenge 3)
Team	Grigoriev-lab
Submission type	☐ INDEL or ☒ SV or ☐ SNV (Only choose one)

1. Read alignment. Did you use the BAM files provided?

☐ Yes ☒ No (Specify your aligner below)

Aligner	BWA	Version	0.7.4
Command lines with parameters	bwa mem -t 32 ./hg19.fa ./synthetic.challenge.set3.normal.1.fastq ./ synthetic.challenge.set3.normal.2.fastq > synthetic.challenge.set3.normal.bwa_mem.sam		

2. Pre-processing steps to calibrate the BAM files. (Command lines and parameters)

1) samtools view -bS -h synthetic.challenge.set3.normal.bwa_mem.sam > synthetic.challenge.set3.normal.bwa_mem.bam
2) samtools sort synthetic.challenge.set3.normal.bwa_mem.bam synthetic.challenge.set3.normal.bwa_mem _sorted
3) samtools index synthetic.challenge.set3.normal.bwa_mem _sorted.bam
4) samtools rmdup synthetic.challenge.set3.normal.bwa_mem _sorted.bam synthetic.challenge.set3.normal.bwa_mem _sorted _rmdup.bam
5) samtools index synthetic.challenge.set3.normal.bwa_mem _sorted _rmdup.bam

3. Mutation calling algorithm.

Algorithm name	GROM	Version	0.0.85

Command lines with parameters	GROM -i synthetic.challenge.set3.normal.bwa_mem _sorted _rmdup.bam -r hg19.fa -o synthetic3_normal_GROM_0_0_85_q0_bwa_mem.txt -p 2 -q 0<br><br>GROM -i synthetic.challenge.set3.tumor.bwa_mem _sorted _rmdup.bam -r hg19.fa -o synthetic3_tumor_GROM_0_0_85_q35_bwa_mem.txt -p 2 -q 35
Thresholds not indicated by command line	
Reference (check if unpublished ☐)	Smith SD, Kawash JK, Grigoriev A. Lightning-fast genome variant detection with GROM. GigaScience. 2017;6:1-7. https://doi.org/10.1093/gigascience/gix091

4. Post-VCF filtering steps. (Command lines and parameters)

python vcf_parser37.py -l -c -d 16 -u 15 -i 35 -s 26 -n synthetic3_normal_GROM_0_0_85_q0_bwa_mem.txt -t synthetic3_tumor_GROM_0_0_85_q35_bwa_mem.txt

5. (Optional) Any other comments or steps not covered.

For vcf_parser (4. Post-VCF filtering steps), -d -u -i -s are thresholds for deletions, duplications, inversions, and insertions, respectively, where a higher value is more stringent.

ICGC-TCGA DREAM Somatic Mutation Calling Challenge
Submission Documentation Form

Submission name	Syn3_V37c_S4 (2478176, synthetic challenge 3)
Team	Grigoriev-lab
Submission type	☐ INDEL or ☒ SV or ☐ SNV (Only choose one)

1. Read alignment. Did you use the BAM files provided?

☐ Yes ☒ No (Specify your aligner below)

Aligner	BWA	Version	0.7.4
Command lines with parameters	bwa mem -t 32 ./hg19.fa ./synthetic.challenge.set3.normal.1.fastq ./synthetic.challenge.set3.normal.2.fastq > synthetic.challenge.set3.normal.bwa_mem.sam		

2. Pre-processing steps to calibrate the BAM files. (Command lines and parameters)

1) samtools view -bS -h synthetic.challenge.set3.normal.bwa_mem.sam > synthetic.challenge.set3.normal.bwa_mem.bam
2) samtools sort synthetic.challenge.set3.normal.bwa_mem.bam synthetic.challenge.set3.normal.bwa_mem _sorted
3) samtools index synthetic.challenge.set3.normal.bwa_mem _sorted.bam
4) samtools rmdup synthetic.challenge.set3.normal.bwa_mem _sorted.bam synthetic.challenge.set3.normal.bwa_mem _sorted _rmdup.bam
5) samtools index synthetic.challenge.set3.normal.bwa_mem _sorted _rmdup.bam

3. Mutation calling algorithm.

Algorithm name	GROM	Version	0.0.85

Command lines with parameters	GROM -i synthetic.challenge.set3.normal.bwa_mem _sorted _rmdup.bam -r hg19.fa -o synthetic3_normal_GROM_0_0_85_q0_bwa_mem.txt -p 2 -q 0<br><br>GROM -i synthetic.challenge.set3.tumor.bwa_mem _sorted _rmdup.bam -r hg19.fa -o synthetic3_tumor_GROM_0_0_85_q35_bwa_mem.txt -p 2 -q 35
Thresholds not indicated by command line	
Reference (check if unpublished ☐)	Smith SD, Kawash JK, Grigoriev A. Lightning-fast genome variant detection with GROM. GigaScience. 2017;6:1-7. https://doi.org/10.1093/gigascience/gix091

4. Post-VCF filtering steps. (Command lines and parameters)

python vcf_parser37.py -l -c -d 18 -u 17 -i 38 -s 31 -n synthetic3_normal_GROM_0_0_85_q0_bwa_mem.txt -t synthetic3_tumor_GROM_0_0_85_q35_bwa_mem.txt

5. (Optional) Any other comments or steps not covered.

For vcf_parser (4. Post-VCF filtering steps), -d -u -i -s are thresholds for deletions, duplications, inversions, and insertions, respectively, where a higher value is more stringent.

ICGC-TCGA DREAM Somatic Mutation Calling Challenge
Submission Documentation Form

Submission name	Syn3_V37_S1 (2478118, synthetic challenge 3)
Team	Grigoriev-lab
Submission type	☐ INDEL or ☒ SV or ☐ SNV (Only choose one)

1. Read alignment. Did you use the BAM files provided?

☐ Yes ☒ No (Specify your aligner below)

Aligner	BWA	Version	0.7.4
Command lines with parameters	bwa mem -t 32 ./hg19.fa ./synthetic.challenge.set3.normal.1.fastq ./synthetic.challenge.set3.normal.2.fastq > synthetic.challenge.set3.normal.bwa_mem.sam		

2. Pre-processing steps to calibrate the BAM files. (Command lines and parameters)

1) samtools view -bS -h synthetic.challenge.set3.normal.bwa_mem.sam > synthetic.challenge.set3.normal.bwa_mem.bam
2) samtools sort synthetic.challenge.set3.normal.bwa_mem.bam synthetic.challenge.set3.normal.bwa_mem _sorted
3) samtools index synthetic.challenge.set3.normal.bwa_mem _sorted.bam
4) samtools rmdup synthetic.challenge.set3.normal.bwa_mem _sorted.bam synthetic.challenge.set3.normal.bwa_mem _sorted _rmdup.bam
5) samtools index synthetic.challenge.set3.normal.bwa_mem _sorted _rmdup.bam

3. Mutation calling algorithm.

Algorithm name	GROM	Version	0.0.85

Command lines with parameters	GROM -i synthetic.challenge.set3.normal.bwa_mem _sorted _rmdup.bam -r hg19.fa -o synthetic3_normal_GROM_0_0_85_q0_bwa_mem.txt -p 2 -q 0<br><br>GROM -i synthetic.challenge.set3.tumor.bwa_mem _sorted _rmdup.bam -r hg19.fa -o synthetic3_tumor_GROM_0_0_85_q35_bwa_mem.txt -p 2 -q 35
Thresholds not indicated by command line	
Reference (check if unpublished ☐)	Smith SD, Kawash JK, Grigoriev A. Lightning-fast genome variant detection with GROM. GigaScience. 2017;6:1-7. https://doi.org/10.1093/gigascience/gix091

4. Post-VCF filtering steps. (Command lines and parameters)

python vcf_parser37.py -l -g 50 -d 17 -u 16 -i 36 -s 31 -n synthetic3_normal_GROM_0_0_85_q0_bwa_mem.txt -t synthetic3_tumor_GROM_0_0_85_q35_bwa_mem.txt

5. (Optional) Any other comments or steps not covered.

For vcf_parser (4. Post-VCF filtering steps), -d -u -i -s are thresholds for deletions, duplications, inversions, and insertions, respectively, where a higher value is more stringent.

ICGC-TCGA DREAM Somatic Mutation Calling Challenge
Submission Documentation Form

Submission name	Syn3_V37_S2 (2478117, synthetic challenge 3)
Team	Grigoriev-lab
Submission type	☐ INDEL or ☒ SV or ☐ SNV (Only choose one)

1. Read alignment. Did you use the BAM files provided?

☐ Yes ☒ No (Specify your aligner below)

Aligner	BWA	Version	0.7.4
Command lines with parameters	bwa mem -t 32 ./hg19.fa ./synthetic.challenge.set3.normal.1.fastq ./synthetic.challenge.set3.normal.2.fastq > synthetic.challenge.set3.normal.bwa_mem.sam		

2. Pre-processing steps to calibrate the BAM files. (Command lines and parameters)

1) samtools view -bS -h synthetic.challenge.set3.normal.bwa_mem.sam > synthetic.challenge.set3.normal.bwa_mem.bam
2) samtools sort synthetic.challenge.set3.normal.bwa_mem.bam synthetic.challenge.set3.normal.bwa_mem _sorted
3) samtools index synthetic.challenge.set3.normal.bwa_mem _sorted.bam
4) samtools rmdup synthetic.challenge.set3.normal.bwa_mem _sorted.bam synthetic.challenge.set3.normal.bwa_mem _sorted _rmdup.bam
5) samtools index synthetic.challenge.set3.normal.bwa_mem _sorted _rmdup.bam

3. Mutation calling algorithm.

Algorithm name	GROM	Version	0.0.85

Command lines with parameters	GROM -i synthetic.challenge.set3.normal.bwa_mem _sorted _rmdup.bam -r hg19.fa -o synthetic3_normal_GROM_0_0_85_q0_bwa_mem.txt -p 2 -q 0<br><br>GROM -i synthetic.challenge.set3.tumor.bwa_mem _sorted _rmdup.bam -r hg19.fa -o synthetic3_tumor_GROM_0_0_85_q35_bwa_mem.txt -p 2 -q 35
Thresholds not indicated by command line	
Reference (check if unpublished ☐)	Smith SD, Kawash JK, Grigoriev A. Lightning-fast genome variant detection with GROM. GigaScience. 2017;6:1-7. https://doi.org/10.1093/gigascience/gix091

4. Post-VCF filtering steps. (Command lines and parameters)

python vcf_parser37.py -l -g 50 -d 13 -u 12 -i 30 -s 12 -n synthetic3_normal_GROM_0_0_85_q0_bwa_mem.txt -t synthetic3_tumor_GROM_0_0_85_q35_bwa_mem.txt

5. (Optional) Any other comments or steps not covered.

For vcf_parser (4. Post-VCF filtering steps), -d -u -i -s are thresholds for deletions, duplications, inversions, and insertions, respectively, where a higher value is more stringent.

ICGC-TCGA DREAM Somatic Mutation Calling Challenge
Submission Documentation Form

Submission name	Syn3_V37SS (2478178, synthetic challenge 3)
Team	Grigoriev-lab
Submission type	☐ INDEL or ☒ SV or ☐ SNV (Only choose one)

1. Read alignment. Did you use the BAM files provided?

☐ Yes ☒ No (Specify your aligner below)

Aligner	BWA	Version	0.7.4
Command lines with parameters	bwa mem -t 32 ./hg19.fa ./synthetic.challenge.set3.normal.1.fastq ./synthetic.challenge.set3.normal.2.fastq > synthetic.challenge.set3.normal.bwa_mem.sam		

2. Pre-processing steps to calibrate the BAM files. (Command lines and parameters)

1) samtools view -bS -h synthetic.challenge.set3.normal.bwa_mem.sam > synthetic.challenge.set3.normal.bwa_mem.bam
2) samtools sort synthetic.challenge.set3.normal.bwa_mem.bam synthetic.challenge.set3.normal.bwa_mem _sorted
3) samtools index synthetic.challenge.set3.normal.bwa_mem _sorted.bam
4) samtools rmdup synthetic.challenge.set3.normal.bwa_mem _sorted.bam synthetic.challenge.set3.normal.bwa_mem _sorted _rmdup.bam
5) samtools index synthetic.challenge.set3.normal.bwa_mem _sorted _rmdup.bam

3. Mutation calling algorithm.

Algorithm name	GROM	Version	0.0.85

Command lines with parameters	GROM -i synthetic.challenge.set3.normal.bwa_mem _sorted _rmdup.bam -r hg19.fa -o synthetic3_normal_GROM_0_0_85_q0_bwa_mem.txt -p 2 -q 0<br><br>GROM -i synthetic.challenge.set3.tumor.bwa_mem _sorted _rmdup.bam -r hg19.fa -o synthetic3_tumor_GROM_0_0_85_q35_bwa_mem.txt -p 2 -q 35
Thresholds not indicated by command line	
Reference (check if unpublished ☐)	Smith SD, Kawash JK, Grigoriev A. Lightning-fast genome variant detection with GROM. GigaScience. 2017;6:1-7. https://doi.org/10.1093/gigascience/gix091

4. Post-VCF filtering steps. (Command lines and parameters)

python vcf_parser37.py -l -c -b -d 17 -u 16 -i 37 -s 30 -n synthetic3_normal_GROM_0_0_85_q0_bwa_mem.txt -t synthetic3_tumor_GROM_0_0_85_q35_bwa_mem.txt

5. (Optional) Any other comments or steps not covered.

For vcf_parser (4. Post-VCF filtering steps), -d -u -i -s are thresholds for deletions, duplications, inversions, and insertions, respectively, where a higher value is more stringent.

ICGC-TCGA DREAM Somatic Mutation Calling Challenge
Submission Documentation Form

Submission name	pavfinder.set1.140324
Team	abyss
Submission type	☐ INDEL or ☒ SV or ☐ SNV (Only choose one)

1. Read alignment. Did you use the BAM files provided?

☒ Yes ☐ No (Specify your aligner below)

Aligner		Version	
Command lines with parameters			

2. Pre-processing steps to calibrate the BAM files. (Command lines and parameters)

```
# input files
TUMOR_INPUT=synthetic.challenge.set1.tumor.v2.bam
NORMAL_INPUT=synthetic.challenge.set1.normal.v2.bam

# extract FASTQs with Picard Tools version 1.105
java -jar SamToFastq.jar INPUT=${TUMOR_INPUT} FASTQ=tumor_1.fq
SECOND_END_FASTQ=tumor_2.fq VALIDATION_STRINGENCY=SILENT
INCLUDE_NON_PF_READS=false
java -jar SamToFastq.jar INPUT=${NORMAL_INPUT} FASTQ=normal_1.fq
SECOND_END_FASTQ=normal_2.fq VALIDATION_STRINGENCY=SILENT
INCLUDE_NON_PF_READS=false
```

3. Mutation calling algorithm.

Algorithm name	PAVFinder	Version	r99287

Command lines with parameters

```
# 1. DE NOVO ASSEMBLY with ABySS

# (i) assemble tumor read pairs into scaffolds
abyss-pe k=50 in="tumor_1.fq tumor_2.fq" name="tumor"

# (ii) break scaffolds into scaftigs
faunscaffold tumor-8.fa > tumor-final.fa

# (iii) extend junctions with `abyss-junction`
abyss-junction -i tumor-5.path -v tumor-5.adj tumor-
3.dist |MergeContigs -v -k 50 --merged <(cat tumor-
3.fa tumor-4.fa tumor-5.fa) tumor-5.adj - |sed -e
's/^>/>J/g' >> tumor-final.fa

{ awk '/^>/ {sub(">", "", $1); print $1}' tumor-
indel.fa; cut -d ' ' -f1 tumor-1.adj; } |sort -n |uniq
-u > tumor-nb.path
```

```
abyss-junction -i tumor-nb.path -v tumor-1.adj |
MergeContigs -v -k 50 --merged tumor-1.fa tumor-1.adj
- |sed -e 's/^>/>I/g' >> tumor-final.fa

# 2. ALIGNMENT with BWA

# (i) align assembly to hg19
bwa mem -a hg19.fa tumor-final.fa |samtools view -hSu
- |samtools sort -o - $TMPDIR > tumor.c2g.bam
samtools index tumor.c2g.bam

# (ii) align tumor read pairs to assembly
bwa index tumor-final.fa
bwa mem tumor-final.fa tumor_1.fq tumor_2.fq |samtools
view -hSu - |samtools sort -o - $TMPDIR >
tumor.r2c.bam
samtools index tumor.r2c.bam

# (iii) align normal read pairs to assembly
bwa mem tumor-final.fa normal_1.fq normal_2.fq |
samtools view -hSu - |samtools sort -o - $TMPDIR >
normal.r2c.bam
samtools index normal.r2c.bam

# 3. CALL SVs with PAVFinder
pavfinder genome tumor.c2g.bam bwa_mem tumor-final.fa
hg19.fa pavfinder_output_dir -b tumor.r2c.bam
--normal_bam normal.r2c.bam --min_size 100
```

Thresholds not indicated by command line	
Reference (check if unpublished ☒)	

4. Post-VCF filtering steps. (Command lines and parameters)

5. (Optional) Any other comments or steps not covered.

The above commands require the following software packages:

1. Picard Tools version 1.105
2. ABySS version 1.3.7
3. faunscaffold < https://github.com/sjackman/fastascripts >
4. BWA version 0.7.4-r385
5. SAMtools version 0.1.19-44428cd
6. PAVFinder version r99287

ICGC-TCGA DREAM Somatic Mutation Calling Challenge
Submission Documentation Form

Submission name	pavfinder.set2.140401
Team	abyss
Submission type	☐ INDEL or ☒ SV or ☐ SNV (Only choose one)

1. Read alignment. Did you use the BAM files provided?

☒ Yes ☐ No (Specify your aligner below)

Aligner		Version	
Command lines with parameters			

2. Pre-processing steps to calibrate the BAM files. (Command lines and parameters)

```
# input files
TUMOR_INPUT=synthetic.challenge.set2.tumor.bam
NORMAL_INPUT=synthetic.challenge.set2.normal.bam

# extract FASTQs with Picard Tools version 1.105
java -jar SamToFastq.jar INPUT=${TUMOR_INPUT} FASTQ=tumor_1.fq
SECOND_END_FASTQ=tumor_2.fq VALIDATION_STRINGENCY=SILENT
INCLUDE_NON_PF_READS=false
java -jar SamToFastq.jar INPUT=${NORMAL_INPUT} FASTQ=normal_1.fq
SECOND_END_FASTQ=normal_2.fq VALIDATION_STRINGENCY=SILENT
INCLUDE_NON_PF_READS=false
```

3. Mutation calling algorithm.

Algorithm name	PAVFinder	Version	r99356

Command lines with parameters

```
# 1. DE NOVO ASSEMBLY with ABySS

# (i) assemble tumor read pairs into scaffolds
abyss-pe k=50 in="tumor_1.fq tumor_2.fq" name="tumor"

# (ii) break scaffolds into scaftigs
faunscaffold tumor-8.fa > tumor-final.fa

# (iii) extend junctions with `abyss-junction`
abyss-junction -i tumor-5.path -v tumor-5.adj tumor-
3.dist |MergeContigs -v -k 50 --merged <(cat tumor-
3.fa tumor-4.fa tumor-5.fa) tumor-5.adj - |sed -e
's/^>/>J/g' >> tumor-final.fa

{ awk '/^>/ {sub(">", "", $1); print $1}' tumor-
indel.fa; cut -d ' ' -f1 tumor-1.adj; } |sort -n |uniq
-u > tumor-nb.path
```

	```<br>abyss-junction -i tumor-nb.path -v tumor-1.adj | MergeContigs -v -k 50 --merged tumor-1.fa tumor-1.adj<br>- |sed -e 's/^>/>I/g' >> tumor-final.fa<br><br># 2. ALIGNMENT with BWA<br><br># (i) align assembly to hg19<br>bwa mem -a hg19.fa tumor-final.fa |samtools view -hSu<br>- |samtools sort -o - $TMPDIR > tumor.c2g.bam<br>samtools index tumor.c2g.bam<br><br># (ii) align tumor read pairs to assembly<br>bwa index tumor-final.fa<br>bwa mem tumor-final.fa tumor_1.fq tumor_2.fq |samtools<br>view -hSu - |samtools sort -o - $TMPDIR ><br>tumor.r2c.bam<br>samtools index tumor.r2c.bam<br><br># (iii) align normal read pairs to assembly<br>bwa mem tumor-final.fa normal_1.fq normal_2.fq | samtools view -hSu - |samtools sort -o - $TMPDIR ><br>normal.r2c.bam<br>samtools index normal.r2c.bam<br><br># 3. CALL SVs with PAVFinder<br>pavfinder genome tumor.c2g.bam bwa_mem tumor-final.fa<br>hg19.fa pavfinder_output_dir -b tumor.r2c.bam<br>--normal_bam normal.r2c.bam --min_size 100<br>```
Thresholds not indicated by command line	
Reference (check if unpublished ☒)	

4. Post-VCF filtering steps. (Command lines and parameters)

5. (Optional) Any other comments or steps not covered.

The above commands require the following software packages:
1. Picard Tools version 1.105
2. ABySS version 1.3.7
3. faunscaffold < https://github.com/sjackman/fastascripts >
4. BWA version 0.7.4-r385
5. SAMtools version 0.1.19-44428cd
6. PAVFinder version r99356

ICGC-TCGA DREAM Somatic Mutation Calling Challenge
Submission Documentation Form

Submission name	pavfinder.set3.140509
Team	abyss
Submission type	☐ INDEL or ☒ SV or ☐ SNV (Only choose one)

1. Read alignment. Did you use the BAM files provided?

☒ Yes ☐ No (Specify your aligner below)

Aligner		Version	
Command lines with parameters			

2. Pre-processing steps to calibrate the BAM files. (Command lines and parameters)

```
# input files
TUMOR_INPUT=synthetic.challenge.set3.tumor.bam
NORMAL_INPUT=synthetic.challenge.set3.normal.bam

# extract FASTQs with Picard Tools
java -jar SamToFastq.jar INPUT=${TUMOR_INPUT} FASTQ=tumor_1.fq
SECOND_END_FASTQ=tumor_2.fq VALIDATION_STRINGENCY=SILENT
INCLUDE_NON_PF_READS=false
java -jar SamToFastq.jar INPUT=${NORMAL_INPUT} FASTQ=normal_1.fq
SECOND_END_FASTQ=normal_2.fq VALIDATION_STRINGENCY=SILENT
INCLUDE_NON_PF_READS=false
```

3. Mutation calling algorithm.

Algorithm name	PAVFinder	Version	r100231

Command lines with parameters

```
# 1. DE NOVO ASSEMBLY with ABySS

# (i) assemble tumor read pairs into scaffolds
abyss-pe k=50 in="tumor_1.fq tumor_2.fq" name="tumor"

# (ii) break scaffolds into scaftigs
faunscaffold tumor-8.fa > tumor-final.fa

# (iii) extend junctions with `abyss-junction`
abyss-junction -i tumor-5.path -v tumor-5.adj tumor-
3.dist |MergeContigs -v -k 50 --merged <(cat tumor-3.fa
tumor-4.fa tumor-5.fa) tumor-5.adj - |sed -e
's/^>/>J/g' >> tumor-final.fa

{ awk '/^>/ {sub(">", "", $1); print $1}' tumor-
indel.fa; cut -d ' ' -f1 tumor-1.adj; } |sort -n |uniq
-u > tumor-nb.path
```

	```<br>abyss-junction -i tumor-nb.path -v tumor-1.adj | MergeContigs -v -k 50 --merged tumor-1.fa tumor-1.adj -<br>|sed -e 's/^>/>I/g' >> tumor-final.fa<br><br># 2. ALIGNMENT with BWA<br><br># (i) align assembly to hg19<br>bwa mem -a hg19.fa tumor-final.fa |samtools view -hSu -<br>|samtools sort -o - $TMPDIR > tumor.c2g.bam<br>samtools index tumor.c2g.bam<br><br># (ii) align tumor read pairs to assembly<br>bwa index tumor-final.fa<br>bwa mem tumor-final.fa tumor_1.fq tumor_2.fq |samtools<br>view -hSu - |samtools sort -o - $TMPDIR > tumor.r2c.bam<br>samtools index tumor.r2c.bam<br><br># (iii) align normal read pairs to assembly<br>bwa mem tumor-final.fa normal_1.fq normal_2.fq | samtools view -hSu - |samtools sort -o - $TMPDIR ><br>normal.r2c.bam<br>samtools index normal.r2c.bam<br><br># 3. CALL SVs with PAVFinder<br>pavfinder genome tumor.c2g.bam bwa_mem tumor-final.fa<br>hg19.fa pavfinder_output_dir -b tumor.r2c.bam<br>--normal_bam normal.r2c.bam --min_size 100<br>```
Thresholds not indicated by command line	
Reference (check if unpublished ☒)	

4. Post-VCF filtering steps. (Command lines and parameters)

5. (Optional) Any other comments or steps not covered.

The above commands require the following software packages:

1. Picard Tools version 1.105
2. ABySS version 1.3.7
3. faunscaffold < https://github.com/sjackman/fastascripts >
4. BWA version 0.7.4-r385
5. SAMtools version 0.1.19-44428cd
6. PAVFinder version r100231

ICGC-TCGA DREAM Somatic Mutation Calling Challenge
Submission Documentation Form

Submission name	dtest
Team	Team2014
Submission type	☒ SV or ☐ SNV (Only choose one)

1. Read alignment. Did you use the BAM files provided?

☒ Yes ☐ No (Specify your aligner below)

Aligner		Version	
Command lines with parameters			

2. Pre-processing steps to calibrate the BAM files. (Command lines and parameters)

No pre-processing steps

3. Mutation calling algorithm.

Algorithm name	Delly	Version	0.3.3

Command lines with parameters

```
#!/bin/bash

for type in DEL DUP INV TRA

do
./delly_v0.3.3_parallel_linux_x86_64bit  -t $type - o set1.$type.vcf  -g
/home/dw670/broad/Homo_sapiens_assembly19.fasta
/home/dw670/dream/synthetic.challenge.set1v2.normal.bam
/home/dw670/dream/synthetic.challenge.set1v2.tumor.bam
done
```

Thresholds not indicated by command line	
Reference (check if unpublished ☐)	Tobias Rausch, Thomas Zichner, Andreas Schlattl, Adrian M. Stuetz, Vladimir Benes, Jan O. Korbel.DELLY: structural variant discovery by integrated paired-end and split-read analysis. Bioinformatics 2012 28: i333-i339.

4. Post-VCF filtering steps. (Command lines and parameters)

```
python ./somaticFilter.py -v set1.DEL.vcf -o set1.DEL.somatic.m100.vcf -t DEL -m 100 –f
python ./somaticFilter.py -v set1.DUP.vcf -o set1.DUP.somatic.m100.vcf -t DUP -m 100 –f
python ./somaticFilter.py -v set1.INV.vcf -o set1.INV.somatic.m100.vcf -t INV -m 100 –f
python ./somaticFilter.py -v set1.TRA.vcf -o set1.TRA.somatic.m100.vcf -t TRA -m 100 -f
```

5. (Optional) Any other comments or steps not covered.

set1.DEL.somatic.m100.vcf, set1.DUP.somatic.m100.vcf, set1.INV.somatic.m100.vcf and set1.TRA.somatic.m100.vcf were merged together as set1.sv.somatic.m100.vcf for submission.

ICGC-TCGA DREAM Somatic Mutation Calling Challenge
Submission Documentation Form

Submission name	dtest
Team	Team2014
Submission type	☒ SV or ☐ SNV (Only choose one)

1. Read alignment. Did you use the BAM files provided?

☒ Yes ☐ No (Specify your aligner below)

Aligner		Version	
Command lines with parameters			

2. Pre-processing steps to calibrate the BAM files. (Command lines and parameters)

No pre-processing steps

3. Mutation calling algorithm.

Algorithm name	Delly	Version	0.3.3

Command lines with parameters

```
#!/bin/bash

for type in DEL DUP INV TRA

do
./delly_v0.3.3_parallel_linux_x86_64bit  -t $type - o set1.$type.vcf  -g
/home/dw670/broad/Homo_sapiens_assembly19.fasta
/home/dw670/dream/synthetic.challenge.set1v2.normal.bam
/home/dw670/dream/synthetic.challenge.set1v2.tumor.bam
done
```

Thresholds not indicated by command line	
Reference (check if unpublished ☐)	Tobias Rausch, Thomas Zichner, Andreas Schlattl, Adrian M. Stuetz, Vladimir Benes, Jan O. Korbel.DELLY: structural variant discovery by integrated paired-end and split-read analysis. Bioinformatics 2012 28: i333-i339.

4. Post-VCF filtering steps. (Command lines and parameters)

python ./somaticFilter.py -v set1.DEL.vcf -o set1.DEL.somatic.m100.vcf -t DEL -m 200 –f
python ./somaticFilter.py -v set1.DUP.vcf -o set1.DUP.somatic.m100.vcf -t DUP -m 200 –f
python ./somaticFilter.py -v set1.INV.vcf -o set1.INV.somatic.m100.vcf -t INV -m 200 –f
python ./somaticFilter.py -v set1.TRA.vcf -o set1.TRA.somatic.m100.vcf -t TRA -m 200 -f

5. (Optional) Any other comments or steps not covered.

set1.DEL.somatic.m200.vcf, set1.DUP.somatic.m200.vcf, set1.INV.somatic.m200.vcf and set1.TRA.somatic.m200.vcf were merged together as set1.sv.somatic.m200.vcf for submission.

ICGC-TCGA DREAM Somatic Mutation Calling Challenge
Submission Documentation Form

Submission name	d9bp
Team	Team2014
Submission type	☒ SV or ☐ SNV (Only choose one)

1. Read alignment. Did you use the BAM files provided?

☒ Yes ☐ No (Specify your aligner below)

Aligner		Version	
Command lines with parameters			

2. Pre-processing steps to calibrate the BAM files. (Command lines and parameters)

No pre-processing steps

3. Mutation calling algorithm.

Algorithm name	Delly	Version	0.3.3

Command lines with parameters

```
#!/bin/bash

for type in DEL DUP INV TRA

do
./delly_v0.3.3_parallel_linux_x86_64bit  -t $type - o set2.$type.vcf  -g
/home/dw670/broad/Homo_sapiens_assembly19.fasta
/home/dw670/dream/synthetic.challenge.set2.normal.bam
/home/dw670/dream/synthetic.challenge.set2.tumor.bam
done
```

Thresholds not indicated by command line	
Reference (check if unpublished ☐)	Tobias Rausch, Thomas Zichner, Andreas Schlattl, Adrian M. Stuetz, Vladimir Benes, Jan O. Korbel.DELLY: structural variant discovery by integrated paired-end and split-read analysis. Bioinformatics 2012 28: i333-i339.

4. Post-VCF filtering steps. (Command lines and parameters)

```
python ./somaticFilter.py -v set2.DEL.vcf -o set2.DEL.somatic.m100.vcf -t DEL -m 100 –f
python ./somaticFilter.py -v set2.DUP.vcf -o set2.DUP.somatic.m100.vcf -t DUP -m 100 –f
python ./somaticFilter.py -v set2.INV.vcf -o set2.INV.somatic.m100.vcf -t INV -m 100 –f
python ./somaticFilter.py -v set2.TRA.vcf -o set2.TRA.somatic.m100.vcf -t TRA -m 100 -f
```

5. (Optional) Any other comments or steps not covered.

```
#!/bin/bash
for type in DEL DUP INV TRA
do
grep 'CIEND=0,0;CIPOS=0,0' set2.$type.somatic.m100.vcf > set2.$type.somatic.m100-0.0.vcf
grep 'CIEND=-1,1;CIPOS=-1,1' set2.$type.somatic.m100.vcf > set2.$type.somatic.m100-1.1.vcf
grep 'CIEND=-2,2;CIPOS=-2,2' set2.$type.somatic.m100.vcf > set2.$type.somatic.m100-2.2.vcf
grep 'CIEND=-3,3;CIPOS=-3,3' set2.$type.somatic.m100.vcf > set2.$type.somatic.m100-3.3.vcf
grep 'CIEND=-3,3;CIPOS=-4,4' set2.$type.somatic.m100.vcf > set2.$type.somatic.m100-4.4.vcf

cat set2.$type.somatic.m100-0.0.vcf >> set2.sv.somatic.m100-9mer.vcf
cat set2.$type.somatic.m100-1.1.vcf >> set2.sv.somatic.m100-9mer.vcf
cat set2.$type.somatic.m100-2.2.vcf >> set2.sv.somatic.m100-9mer.vcf
cat set2.$type.somatic.m100-3.3.vcf >> set2.sv.somatic.m100-9mer.vcf
cat set2.$type.somatic.m100-4.4.vcf >> set2.sv.somatic.m100-9mer.vcf

done
```

ICGC-TCGA DREAM Somatic Mutation Calling Challenge
Submission Documentation Form

Submission name	dhqpres
Team	Team2014
Submission type	☒ SV or ☐ SNV (Only choose one)

1. Read alignment. Did you use the BAM files provided?

☒ Yes ☐ No (Specify your aligner below)

Aligner		Version	
Command lines with parameters			

2. Pre-processing steps to calibrate the BAM files. (Command lines and parameters)

No pre-processing steps

3. Mutation calling algorithm.

Algorithm name	Delly	Version	0.3.3

Command lines with parameters

```
#!/bin/bash

for type in DEL DUP INV TRA

do
./delly_v0.3.3_parallel_linux_x86_64bit  -t $type - o set2.$type.vcf  -g
/home/dw670/broad/Homo_sapiens_assembly19.fasta
/home/dw670/dream/synthetic.challenge.set2.normal.bam
/home/dw670/dream/synthetic.challenge.set2.tumor.bam
done
```

Thresholds not indicated by command line	
Reference (check if unpublished ☐)	Tobias Rausch, Thomas Zichner, Andreas Schlattl, Adrian M. Stuetz, Vladimir Benes, Jan O. Korbel.DELLY: structural variant discovery by integrated paired-end and split-read analysis. Bioinformatics 2012 28: i333-i339.

4. Post-VCF filtering steps. (Command lines and parameters)

```
#!/bin/bash
for type in DEL DUP INV TRA
do
python ./somaticFilter.py -v set2.$type.vcf -o set2.DEL.somatic.m100.vcf -t $type -m 100 –f
cat set2.$type.somatic.m100.vcf >> set2.sv.somatic.m100.vcf
done
```

5. (Optional) Any other comments or steps not covered.

From set2.sv.somatic.m100.vcf

1> Extract 'CIEND=-1,1;CIPOS=-1,1' records.
2> removed LowQual records.
3> removed IMPRECISE records.
4> extract PASS records
5> generated set2.sv.somatic.m100.sub2.vcf for submission.

ICGC-TCGA DREAM Somatic Mutation Calling Challenge
Submission Documentation Form

Submission name	dtest_m100
Team	Team2014
Submission type	☒ SV or ☐ SNV (Only choose one)

1. Read alignment. Did you use the BAM files provided?

☒ Yes ☐ No (Specify your aligner below)

Aligner		Version	
Command lines with parameters			

2. Pre-processing steps to calibrate the BAM files. (Command lines and parameters)

No pre-processing steps

3. Mutation calling algorithm.

Algorithm name	Delly	Version	0.3.3

Command lines with parameters

```
#!/bin/bash

for type in DEL DUP INV TRA

do
./delly_v0.3.3_parallel_linux_x86_64bit  -t $type - o set3.$type.vcf  -g
/home/dw670/broad/Homo_sapiens_assembly19.fasta
/home/dw670/dream/synthetic.challenge.set3.normal.bam
/home/dw670/dream/synthetic.challenge.set3.tumor.bam
done
```

Thresholds not indicated by command line	
Reference (check if unpublished ☐)	Tobias Rausch, Thomas Zichner, Andreas Schlattl, Adrian M. Stuetz, Vladimir Benes, Jan O. Korbel.DELLY: structural variant discovery by integrated paired-end and split-read analysis. Bioinformatics 2012 28: i333-i339.

4. Post-VCF filtering steps. (Command lines and parameters)

python ./somaticFilter.py -v set3.DEL.vcf -o set3.DEL.somatic.m100.vcf -t DEL -m 100 –f
python ./somaticFilter.py -v set3.DUP.vcf -o set3.DUP.somatic.m100.vcf -t DUP -m 100 –f
python ./somaticFilter.py -v set3.INV.vcf -o set3.INV.somatic.m100.vcf -t INV -m 100 –f
python ./somaticFilter.py -v set3.TRA.vcf -o set3.TRA.somatic.m100.vcf -t TRA -m 100 -f

5. (Optional) Any other comments or steps not covered.

set3.DEL.somatic.m100.vcf, set3.DUP.somatic.m100.vcf, set3.INV.somatic.m100.vcf and set3.TRA.somatic.m100.vcf were merged together as set13.sv.somatic.m100.vcf for submission.

ICGC-TCGA DREAM Somatic Mutation Calling Challenge
Submission Documentation Form

Submission name	dtest
Team	Team2014
Submission type	☒ SV or ☐ SNV (Only choose one)

1. Read alignment. Did you use the BAM files provided?

☒ Yes ☐ No (Specify your aligner below)

Aligner		Version	
Command lines with parameters			

2. Pre-processing steps to calibrate the BAM files. (Command lines and parameters)

No pre-processing steps

3. Mutation calling algorithm.

Algorithm name	Delly	Version	0.3.3

Command lines with parameters

```
#!/bin/bash

for type in DEL DUP INV TRA

do
./delly_v0.3.3_parallel_linux_x86_64bit  -t $type - o set2.$type.vcf  -g
/home/dw670/broad/Homo_sapiens_assembly19.fasta
/home/dw670/dream/synthetic.challenge.set2.normal.bam
/home/dw670/dream/synthetic.challenge.set2.tumor.bam
done
```

Thresholds not indicated by command line	
Reference (check if unpublished ☐)	Tobias Rausch, Thomas Zichner, Andreas Schlattl, Adrian M. Stuetz, Vladimir Benes, Jan O. Korbel.DELLY: structural variant discovery by integrated paired-end and split-read analysis. Bioinformatics 2012 28: i333-i339.

4. Post-VCF filtering steps. (Command lines and parameters)

```
python ./somaticFilter.py -v set3.DEL.vcf -o set3.DEL.somatic.m100.vcf -t DEL -m 200 –f
python ./somaticFilter.py -v set3.DUP.vcf -o set3.DUP.somatic.m100.vcf -t DUP -m 200 –f
python ./somaticFilter.py -v set3.INV.vcf -o set3.INV.somatic.m100.vcf -t INV -m 200 –f
python ./somaticFilter.py -v set3.TRA.vcf -o set3.TRA.somatic.m100.vcf -t TRA -m 200 -f
```

5. (Optional) Any other comments or steps not covered.

set3.DEL.somatic.m200.vcf, set3.DUP.somatic.m200.vcf, set3.INV.somatic.m200.vcf and set3.TRA.somatic.m200.vcf were merged together as set13.sv.somatic.m200.vcf for submission.

ICGC-TCGA DREAM Somatic Mutation Calling Challenge
Submission Documentation Form

Submission name	destruct-8 (ID: 2385623)
Team	SFU
Submission type	☒ SV or ☐ SNV (Only choose one)

1. Read alignment. Did you use the BAM files provided?

☒ Yes ☐ No (Specify your aligner below)

Aligner		Version	
Command lines with parameters			

2. Pre-processing steps to calibrate the BAM files. (Command lines and parameters)

3. Mutation calling algorithm.

Algorithm name	deStruct	Version	0.1.0
Command lines with parameters	python destruct.py –myconfig.ini --tmpdir silico2-temp --submit asyncqsub --maxjobs 100 --repopulate --verbose silico2.lib silico2-result/breakpoints silico2-result/breakreads		

Thresholds not indicated by command line	
Reference (check if unpublished ☒)	

4. Post-VCF filtering steps. (Command lines and parameters)

5. (Optional) Any other comments or steps not covered.

After execution of deStruct, the output file 'breakpoints' is converted to VCF format using 'destruct2VCF.py' supplementary tool.

Alignment probability is set to be higher than 0.8; chimeric and valid probabilities are set to be higher than 0.5. Calls with tumor read support below 3 are filtered:

python destruct2VCF.py -b silico2-result/breakpoints -f silico2-result/filtered-reads -o silico2-result/test.vcf -a 0.8 -c 0.5 -v 0.5 -t 3

ICGC-TCGA DREAM Somatic Mutation Calling Challenge
Submission Documentation Form

Submission name	destruct-8-o
Team	SFU
Submission type	☒ SV or ☐ SNV (Only choose one)

1. Read alignment. Did you use the BAM files provided?			
☒ Yes	☐ No (Specify your aligner below)		
Aligner		Version	
Command lines with parameters			

2. Pre-processing steps to calibrate the BAM files. (Command lines and parameters)

3. Mutation calling algorithm.			
Algorithm name	deStruct	Version	0.1.0
Command lines with parameters	python destruct.py –myconfig.ini --tmpdir silico2-temp --submit asyncqsub --maxjobs 100 --repopulate --verbose silico2.lib silico2-result/breakpoints silico2-result/breakreads		

Thresholds not indicated by command line	
Reference (check if unpublished ☒)	

4. Post-VCF filtering steps. (Command lines and parameters)

5. (Optional) Any other comments or steps not covered.

After execution of deStruct, the output file 'breakpoints' is converted to VCF format using 'destruct2VCF.py' supplementary tool.

Alignment probability is set to be higher than 0.8; chimeric and valid probabilities are set to be higher than 0.5:

python destruct2VCF.py -b silico2-result/breakpoints -f silico2-result/filtered-reads -o silico2-result/original.vcf -a 0.8 -c 0.5 -v 0.5 -t 0

ICGC-TCGA DREAM Somatic Mutation Calling Challenge
Submission Documentation Form

Submission name	destruct-8 (ID: 2385598)
Team	SFU
Submission type	☒ SV or ☐ SNV (Only choose one)

1. Read alignment. Did you use the BAM files provided?

☒ Yes ☐ No (Specify your aligner below)

Aligner		Version	
Command lines with parameters			

2. Pre-processing steps to calibrate the BAM files. (Command lines and parameters)

3. Mutation calling algorithm.

Algorithm name	deStruct	Version	0.1.0
Command lines with parameters	python destruct.py –myconfig.ini --tmpdir silico2-temp --submit asyncqsub --maxjobs 100 --repopulate --verbose silico2.lib silico2-result/breakpoints silico2-result/breakreads		

Thresholds not indicated by command line	
Reference (check if unpublished ☒)	

4. Post-VCF filtering steps. (Command lines and parameters)

5. (Optional) Any other comments or steps not covered.

After execution of deStruct, the output file 'breakpoints' is converted to VCF format using 'destruct2VCF.py' supplementary tool.

Alignment probability is set to be higher than 0.8; chimeric and valid probabilities are set to be higher than 0.5. Calls with tumor read support below 2 are filtered:

```
python destruct2VCF.py -b silico2-result/breakpoints -f silico2-result/filtered-reads -o silico2-result/result.vcf -a 0.8 -c 0.5 -v 0.5 -t 2
```

ICGC-TCGA DREAM Somatic Mutation Calling Challenge
Submission Documentation Form

Submission name	destruct-8-q
Team	SFU
Submission type	☒ SV or ☐ SNV (Only choose one)

1. Read alignment. Did you use the BAM files provided?

☒ Yes ☐ No (Specify your aligner below)

Aligner		Version	
Command lines with parameters			

2. Pre-processing steps to calibrate the BAM files. (Command lines and parameters)

3. Mutation calling algorithm.

Algorithm name	deStruct	Version	0.1.0
Command lines with parameters	python destruct.py –myconfig.ini --tmpdir silico2-temp --submit asyncqsub --maxjobs 100 --repopulate --verbose silico2.lib silico2-result/breakpoints silico2-result/breakreads		

Thresholds not indicated by command line	
Reference (check if unpublished ☒)	

4. Post-VCF filtering steps. (Command lines and parameters)

5. (Optional) Any other comments or steps not covered.

After execution of deStruct, the output file 'breakpoints' is converted to VCF format using 'destruct2VCF.py' supplementary tool.

Alignment probability is set to be higher than 0.8:

python destruct2VCF.py -b silico2-result/breakpoints -f silico2-result/filtered-reads -o silico2-result/noqfilt.vcf -a 0.8 -c 0 -v 0 -t 0

ICGC-TCGA DREAM Somatic Mutation Calling Challenge
Submission Documentation Form

Submission name	destruct-align_prob_filter
Team	SFU
Submission type	☒ SV or ☐ SNV (Only choose one)

1. Read alignment. Did you use the BAM files provided?

☒ Yes ☐ No (Specify your aligner below)

Aligner		Version	
Command lines with parameters			

2. Pre-processing steps to calibrate the BAM files. (Command lines and parameters)

3. Mutation calling algorithm.

Algorithm name	deStruct	Version	0.1.0
Command lines with parameters	python destruct.py –myconfig.ini --tmpdir silico2-temp --submit asyncqsub --maxjobs 100 --repopulate --verbose silico2.lib silico2-result/breakpoints silico2-result/breakreads		

Thresholds not indicated by command line	
Reference (check if unpublished ☒)	

4. Post-VCF filtering steps. (Command lines and parameters)

5. (Optional) Any other comments or steps not covered.

After execution of deStruct, the output file 'breakpoints' is converted to VCF format using 'destruct2VCF.py' supplementary tool.

Calls with alignment probability below 0.9 are filtered:

python destruct2VCF.py -b silico2-result/breakpoints -f silico2-result/filtered-reads -o silico2-result/result-gt0.9.vcf -a 0.9 -c 0 -v 0 -t 0

ICGC-TCGA DREAM Somatic Mutation Calling Challenge
Submission Documentation Form

Submission name	destruct-chimeric_prob_filter
Team	SFU
Submission type	☒ SV or ☐ SNV (Only choose one)

1. Read alignment. Did you use the BAM files provided?

☒ Yes ☐ No (Specify your aligner below)

Aligner		Version	
Command lines with parameters			

2. Pre-processing steps to calibrate the BAM files. (Command lines and parameters)

3. Mutation calling algorithm.

Algorithm name	deStruct	Version	0.1.0
Command lines with parameters	python destruct.py –myconfig.ini --tmpdir silico2-temp --submit asyncqsub --maxjobs 100 --repopulate --verbose silico2.lib silico2-result/breakpoints silico2-result/breakreads		

Thresholds not indicated by command line	
Reference (check if unpublished ☒)	

4. Post-VCF filtering steps. (Command lines and parameters)

5. (Optional) Any other comments or steps not covered.

After execution of deStruct, the output file 'breakpoints' is converted to VCF format using 'destruct2VCF.py' supplementary tool.

Calls with chimeric probability below 0.9 are filtered:

```
python destruct2VCF.py -b silico2-result/breakpoints -f silico2-result/filtered-reads -o silico2-result/result-gt0.9.vcf -a 0 -c 0.9 -v 0 -t 0
```

ICGC-TCGA DREAM Somatic Mutation Calling Challenge
Submission Documentation Form

Submission name	destruct-default_g_excluded
Team	SFU
Submission type	☒ SV or ☐ SNV (Only choose one)

1. Read alignment. Did you use the BAM files provided?

☒ Yes ☐ No (Specify your aligner below)

Aligner		Version	
Command lines with parameters			

2. Pre-processing steps to calibrate the BAM files. (Command lines and parameters)

3. Mutation calling algorithm.

Algorithm name	deStruct	Version	0.1.0
Command lines with parameters	python destruct.py --myconfig.ini --tmpdir silico2-temp --submit asyncqsub --maxjobs 100 --repopulate --verbose silico2.lib silico2-result/breakpoints silico2-result/breakreads		

Thresholds not indicated by command line	
Reference (check if unpublished ☒)	

4. Post-VCF filtering steps. (Command lines and parameters)

5. (Optional) Any other comments or steps not covered.

After execution of deStruct, low quality calls are manually filtered from the 'breakpoints' output file. Then the output is converted to VCF format using 'destruct2VCF.py' supplementary tool.

Alignment, chimeric and valid probabilities are set to be higher than 0.5:

python destruct2VCF.py -b silico2-result/breakpoints -f silico2-result/filtered-reads -o silico2-result/exclude-greens.vcf -a 0.5 -c 0.5 -v 0.5 -t 0

ICGC-TCGA DREAM Somatic Mutation Calling Challenge
Submission Documentation Form

Submission name	destruct-default
Team	SFU
Submission type	☒ SV or ☐ SNV (Only choose one)

1. Read alignment. Did you use the BAM files provided?

☒ Yes ☐ No (Specify your aligner below)

Aligner		Version	
Command lines with parameters			

2. Pre-processing steps to calibrate the BAM files. (Command lines and parameters)

3. Mutation calling algorithm.

Algorithm name	deStruct	Version	0.1.0
Command lines with parameters	python destruct.py --myconfig.ini --tmpdir silico2-temp --submit asyncqsub --maxjobs 100 --repopulate --verbose silico2.lib silico2-result/breakpoints silico2-result/breakreads		

Thresholds not indicated by command line	
Reference (check if unpublished ☒)	

4. Post-VCF filtering steps. (Command lines and parameters)

5. (Optional) Any other comments or steps not covered.

After execution of deStruct, the output file 'breakpoints' is converted to VCF format using 'destruct2VCF.py' supplementary tool.

Alignment, chimeric and valid probabilities are set to be higher than 0.5:

python destruct2VCF.py -b silico2-result/breakpoints -f silico2-result/filtered-reads -o silico2-result/result.vcf -a 0.5 -c 0.5 -v 0.5 -t 0

ICGC-TCGA DREAM Somatic Mutation Calling Challenge
Submission Documentation Form

Submission name	destruct-final2
Team	SFU
Submission type	☒ SV or ☐ SNV (Only choose one)

1. Read alignment. Did you use the BAM files provided?

☒ Yes ☐ No (Specify your aligner below)

Aligner		Version	
Command lines with parameters			

2. Pre-processing steps to calibrate the BAM files. (Command lines and parameters)

3. Mutation calling algorithm.

Algorithm name	deStruct	Version	0.1.0
Command lines with parameters	python destruct.py –myconfig.ini --tmpdir silico2-temp --submit asyncqsub --maxjobs 100 --repopulate --verbose silico2.lib silico2-result/breakpoints silico2-result/breakreads		

Thresholds not indicated by command line	
Reference (check if unpublished ☒)	

4. Post-VCF filtering steps. (Command lines and parameters)

5. (Optional) Any other comments or steps not covered.

After execution of deStruct, the output file 'breakpoints' is converted to VCF format using 'destruct2VCF.py' supplementary tool.

Alignment, chimeric and valid probabilities are set to be higher than 0.8. Calls with tumor read support below 2 are filtered:

python destruct2VCF.py -b silico2-result/breakpoints -f silico2-result/filtered-reads -o silico2-result/destruct_tumour_2.vcf -a 0.8 -c 0.8 -v 0.8 -t 2

ICGC-TCGA DREAM Somatic Mutation Calling Challenge
Submission Documentation Form

Submission name	destruct-final3
Team	SFU
Submission type	☒ SV or ☐ SNV (Only choose one)

1. Read alignment. Did you use the BAM files provided?

☒ Yes ☐ No (Specify your aligner below)

Aligner		Version	
Command lines with parameters			

2. Pre-processing steps to calibrate the BAM files. (Command lines and parameters)

3. Mutation calling algorithm.

Algorithm name	deStruct	Version	0.1.0
Command lines with parameters	python destruct.py –myconfig.ini --tmpdir silico2-temp --submit asyncqsub --maxjobs 100 --repopulate --verbose silico2.lib silico2-result/breakpoints silico2-result/breakreads		

Thresholds not indicated by command line	
Reference (check if unpublished ☒)	

4. Post-VCF filtering steps. (Command lines and parameters)

5. (Optional) Any other comments or steps not covered.

After execution of deStruct, the output file 'breakpoints' is converted to VCF format using 'destruct2VCF.py' supplementary tool.

Alignment, chimeric and valid probabilities are set to be higher than 0.8. Calls with tumor read support below 3 are filtered:

python destruct2VCF.py -b silico2-result/breakpoints -f silico2-result/filtered-reads -o silico2-result/destruct_tumour_3.vcf -a 0.8 -c 0.8 -v 0.8 -t 3

ICGC-TCGA DREAM Somatic Mutation Calling Challenge
Submission Documentation Form

Submission name	destruct-final4
Team	SFU
Submission type	☒ SV or ☐ SNV (Only choose one)

1. Read alignment. Did you use the BAM files provided?

☒ Yes ☐ No (Specify your aligner below)

Aligner		Version	
Command lines with parameters			

2. Pre-processing steps to calibrate the BAM files. (Command lines and parameters)

3. Mutation calling algorithm.

Algorithm name	deStruct	Version	0.1.0
Command lines with parameters	python destruct.py –myconfig.ini --tmpdir silico2-temp --submit asyncqsub --maxjobs 100 --repopulate --verbose silico2.lib silico2-result/breakpoints silico2-result/breakreads		

Thresholds not indicated by command line	
Reference (check if unpublished ☒)	

4. Post-VCF filtering steps. (Command lines and parameters)

5. (Optional) Any other comments or steps not covered.

After execution of deStruct, the output file 'breakpoints' is converted to VCF format using 'destruct2VCF.py' supplementary tool.

Alignment, chimeric and valid probabilities are set to be higher than 0.8. Calls with tumor read support below 4 are filtered:

python destruct2VCF.py -b silico2-result/breakpoints -f silico2-result/filtered-reads -o silico2-result/destruct_tumour_4.vcf -a 0.8 -c 0.8 -v 0.8 -t 4

ICGC-TCGA DREAM Somatic Mutation Calling Challenge
Submission Documentation Form

Submission name	destruct-noqfilt
Team	SFU
Submission type	☒ SV or ☐ SNV (Only choose one)

1. Read alignment. Did you use the BAM files provided?

☒ Yes ☐ No (Specify your aligner below)

Aligner		Version	
Command lines with parameters			

2. Pre-processing steps to calibrate the BAM files. (Command lines and parameters)

3. Mutation calling algorithm.

Algorithm name	deStruct	Version	0.1.0
Command lines with parameters	python destruct.py –myconfig.ini --tmpdir silico2-temp --submit asyncqsub --maxjobs 100 --repopulate --verbose silico2.lib silico2-result/breakpoints silico2-result/breakreads		

Thresholds not indicated by command line	
Reference (check if unpublished ☒)	

4. Post-VCF filtering steps. (Command lines and parameters)

5. (Optional) Any other comments or steps not covered.

After execution of deStruct, the output file 'breakpoints' is converted to VCF format using 'destruct2VCF.py' supplementary tool.

Alignment, chimeric and valid probabilities are set to be higher than 0.7. Calls with tumor read support below 2 are filtered:

python destruct2VCF.py -b silico2-result/breakpoints -f silico2-result/filtered-reads -o silico2-result/noqfilt.vcf -a 0.7 -c 0.7 -v 0.7 -t 2

ICGC-TCGA DREAM Somatic Mutation Calling Challenge
Submission Documentation Form

Submission name	destruct-split_read_filter
Team	SFU
Submission type	☒ SV or ☐ SNV (Only choose one)

1. Read alignment. Did you use the BAM files provided?

☒ Yes ☐ No (Specify your aligner below)

Aligner		Version	
Command lines with parameters			

2. Pre-processing steps to calibrate the BAM files. (Command lines and parameters)

3. Mutation calling algorithm.

Algorithm name	deStruct	Version	0.1.0
Command lines with parameters	python destruct.py --myconfig.ini --tmpdir silico2-temp --submit asyncqsub --maxjobs 100 --repopulate --verbose silico2.lib silico2-result/breakpoints silico2-result/breakreads		

Thresholds not indicated by command line	
Reference (check if unpublished ☒)	

4. Post-VCF filtering steps. (Command lines and parameters)

5. (Optional) Any other comments or steps not covered.

After execution of deStruct, the output file 'breakpoints' is converted to VCF format using 'destruct2VCF.py' supplementary tool.

Alignment, chimeric and valid probabilities are set to be higher than 0.5. Also calls not supported by split read mappings are filtered from output:

python destruct2VCF.py -b silico2-result/breakpoints -f silico2-result/filtered-reads -o silico2-result/result.vcf -a 0.5 -c 0.5 -v 0.5 -t 0

ICGC-TCGA DREAM Somatic Mutation Calling Challenge
Submission Documentation Form

Submission name	destruct-test5
Team	SFU
Submission type	☒ SV or ☐ SNV (Only choose one)

1. Read alignment. Did you use the BAM files provided?

☒ Yes ☐ No (Specify your aligner below)

Aligner		Version	
Command lines with parameters			

2. Pre-processing steps to calibrate the BAM files. (Command lines and parameters)

3. Mutation calling algorithm.

Algorithm name	deStruct	Version	0.1.0
Command lines with parameters	python destruct.py –myconfig.ini --tmpdir silico2-temp --submit asyncqsub --maxjobs 100 --repopulate --verbose silico2.lib silico2-result/breakpoints silico2-result/breakreads		

Thresholds not indicated by command line	
Reference (check if unpublished ☒)	

4. Post-VCF filtering steps. (Command lines and parameters)

5. (Optional) Any other comments or steps not covered.

After execution of deStruct, the output file 'breakpoints' is converted to VCF format using 'destruct2VCF.py' supplementary tool.

Alignment, chimeric and valid probabilities are set to be higher than 0.7. Calls with tumor read support below 3 are filtered:

python destruct2VCF.py -b silico2-result/breakpoints -f silico2-result/filtered-reads -o silico2-result/original5.vcf -a 0.7 -c 0.7 -v 0.7 -t 3

ICGC-TCGA DREAM Somatic Mutation Calling Challenge
Submission Documentation Form

Submission name	destruct-test6
Team	SFU
Submission type	☒ SV or ☐ SNV (Only choose one)

1. Read alignment. Did you use the BAM files provided?

☒ Yes ☐ No (Specify your aligner below)

Aligner		Version	
Command lines with parameters			

2. Pre-processing steps to calibrate the BAM files. (Command lines and parameters)

3. Mutation calling algorithm.

Algorithm name	deStruct	Version	0.1.0
Command lines with parameters	python destruct.py –myconfig.ini --tmpdir silico2-temp --submit asyncqsub --maxjobs 100 --repopulate --verbose silico2.lib silico2-result/breakpoints silico2-result/breakreads		

Thresholds not indicated by command line	
Reference (check if unpublished ☒)	

4. Post-VCF filtering steps. (Command lines and parameters)

5. (Optional) Any other comments or steps not covered.

After execution of deStruct, the output file 'breakpoints' is converted to VCF format using 'destruct2VCF.py' supplementary tool.

Alignment, chimeric and valid probabilities are set to be higher than 0.7. Calls with tumor read support below 4 are filtered:

python destruct2VCF.py -b silico2-result/breakpoints -f silico2-result/filtered-reads -o silico2-result/original5.vcf -a 0.7 -c 0.7 -v 0.7 -t 4

ICGC-TCGA DREAM Somatic Mutation Calling Challenge
Submission Documentation Form

Submission name	destruct-tumor_count_filter
Team	SFU
Submission type	☒ SV or ☐ SNV (Only choose one)

1. Read alignment. Did you use the BAM files provided?

☒ Yes ☐ No (Specify your aligner below)

Aligner		Version	
Command lines with parameters			

2. Pre-processing steps to calibrate the BAM files. (Command lines and parameters)

3. Mutation calling algorithm.

Algorithm name	deStruct	Version	0.1.0
Command lines with parameters	python destruct.py –myconfig.ini --tmpdir silico2-temp --submit asyncqsub --maxjobs 100 --repopulate --verbose silico2.lib silico2-result/breakpoints silico2-result/breakreads		

Thresholds not indicated by command line	
Reference (check if unpublished ☒)	

4. Post-VCF filtering steps. (Command lines and parameters)

5. (Optional) Any other comments or steps not covered.

After execution of deStruct, the output file 'breakpoints' is converted to VCF format using 'destruct2VCF.py' supplementary tool.

Alignment, chimeric and valid probabilities are set to be higher than 0.5, and calls with tumor read support below 4 are filtered:

python destruct2VCF.py -b silico2-result/breakpoints -f silico2-result/filtered-reads -o silico2-result/result-gt4.vcf -a 0.5 -c 0.5 -v 0.5 -t 4

ICGC-TCGA DREAM Somatic Mutation Calling Challenge
Submission Documentation Form

Submission name	destruct-tumor_filter_del
Team	SFU
Submission type	☒ SV or ☐ SNV (Only choose one)

1. Read alignment. Did you use the BAM files provided?

☒ Yes ☐ No (Specify your aligner below)

Aligner		Version	
Command lines with parameters			

2. Pre-processing steps to calibrate the BAM files. (Command lines and parameters)

3. Mutation calling algorithm.

Algorithm name	deStruct	Version	0.1.0
Command lines with parameters	python destruct.py --myconfig.ini --tmpdir silico2-temp --submit asyncqsub --maxjobs 100 --repopulate --verbose silico2.lib silico2-result/breakpoints silico2-result/breakreads		

Thresholds not indicated by command line	
Reference (check if unpublished ☒)	

4. Post-VCF filtering steps. (Command lines and parameters)

5. (Optional) Any other comments or steps not covered.

After execution of deStruct, the output file 'breakpoints' is converted to VCF format using 'destruct2VCF.py' supplementary tool.

Alignment, chimeric and valid probabilities are set to be higher than 0.5. Output only contains deletion calls supported by tumor sample:

python destruct2VCF.py -b silico2-result/breakpoints -f silico2-result/filtered-reads -o silico2-result/del.vcf -a 0.5 -c 0.5 -v 0.5 -t 0

ICGC-TCGA DREAM Somatic Mutation Calling Challenge
Submission Documentation Form

Submission name	destruct-tumor_filter_dup
Team	SFU
Submission type	☒ SV or ☐ SNV (Only choose one)

1. Read alignment. Did you use the BAM files provided?

☒ Yes ☐ No (Specify your aligner below)

Aligner		Version	
Command lines with parameters			

2. Pre-processing steps to calibrate the BAM files. (Command lines and parameters)

3. Mutation calling algorithm.

Algorithm name	deStruct	Version	0.1.0
Command lines with parameters	python destruct.py --myconfig.ini --tmpdir silico2-temp --submit asyncqsub --maxjobs 100 --repopulate --verbose silico2.lib silico2-result/breakpoints silico2-result/breakreads		

Thresholds not indicated by command line	
Reference (check if unpublished ☒)	

4. Post-VCF filtering steps. (Command lines and parameters)

5. (Optional) Any other comments or steps not covered.

After execution of deStruct, the output file 'breakpoints' is converted to VCF format using 'destruct2VCF.py' supplementary tool.

Alignment, chimeric and valid probabilities are set to be higher than 0.5. Output only contains duplication calls supported by tumor sample.

python destruct2VCF.py -b silico2-result/breakpoints -f silico2-result/filtered-reads -o silico2-result/del.vcf -a 0.5 -c 0.5 -v 0.5 -t 0

ICGC-TCGA DREAM Somatic Mutation Calling Challenge
Submission Documentation Form

Submission name	destruct-tumor_filter_inv
Team	SFU
Submission type	☒ SV or ☐ SNV (Only choose one)

1. Read alignment. Did you use the BAM files provided?

☒ Yes ☐ No (Specify your aligner below)

Aligner		Version	
Command lines with parameters			

2. Pre-processing steps to calibrate the BAM files. (Command lines and parameters)

3. Mutation calling algorithm.

Algorithm name	deStruct	Version	0.1.0
Command lines with parameters	python destruct.py --myconfig.ini --tmpdir silico2-temp --submit asyncqsub --maxjobs 100 --repopulate --verbose silico2.lib silico2-result/breakpoints silico2-result/breakreads		

Thresholds not indicated by command line	
Reference (check if unpublished ☒)	

4. Post-VCF filtering steps. (Command lines and parameters)

5. (Optional) Any other comments or steps not covered.

After execution of deStruct, the output file 'breakpoints' is converted to VCF format using 'destruct2VCF.py' supplementary tool.

Alignment, chimeric and valid probabilities are set to be higher than 0.5. Output only contains inversion calls supported by tumor sample.

python destruct2VCF.py -b silico2-result/breakpoints -f silico2-result/filtered-reads -o silico2-result/del.vcf -a 0.5 -c 0.5 -v 0.5 -t 0

ICGC-TCGA DREAM Somatic Mutation Calling Challenge
Submission Documentation Form

Submission name	destruct-valid_prob_filter
Team	SFU
Submission type	☒ SV or ☐ SNV (Only choose one)

1. Read alignment. Did you use the BAM files provided?

☒ Yes ☐ No (Specify your aligner below)

Aligner		Version	
Command lines with parameters			

2. Pre-processing steps to calibrate the BAM files. (Command lines and parameters)

3. Mutation calling algorithm.

Algorithm name	deStruct	Version	0.1.0
Command lines with parameters	python destruct.py –myconfig.ini --tmpdir silico2-temp --submit asyncqsub --maxjobs 100 --repopulate --verbose silico2.lib silico2-result/breakpoints silico2-result/breakreads		

Thresholds not indicated by command line	
Reference (check if unpublished ☒)	

4. Post-VCF filtering steps. (Command lines and parameters)

5. (Optional) Any other comments or steps not covered.

After execution of deStruct, the output file 'breakpoints' is converted to VCF format using 'destruct2VCF.py' supplementary tool. Calls with valid probability below 0.9 are filtered:

```
python destruct2VCF.py -b silico2-result/breakpoints -f silico2-result/filtered-reads -o silico2-result/result-gt0.9.vcf -a 0 -c 0 -v 0.9 -t 0
```

ICGC-TCGA DREAM Somatic Mutation Calling Challenge
Submission Documentation Form

Submission name	destruct-vcftest1 (ID: 2385643)
Team	SFU
Submission type	☒ SV or ☐ SNV (Only choose one)

1. Read alignment. Did you use the BAM files provided?

☒ Yes ☐ No (Specify your aligner below)

Aligner		Version	
Command lines with parameters			

2. Pre-processing steps to calibrate the BAM files. (Command lines and parameters)

3. Mutation calling algorithm.

Algorithm name	deStruct	Version	0.1.0
Command lines with parameters	python destruct.py –myconfig.ini --tmpdir silico2-temp --submit asyncqsub --maxjobs 100 --repopulate --verbose silico2.lib silico2-result/breakpoints silico2-result/breakreads		

Thresholds not indicated by command line	
Reference (check if unpublished ☒)	

4. Post-VCF filtering steps. (Command lines and parameters)

5. (Optional) Any other comments or steps not covered.

After execution of deStruct, the output file 'breakpoints' is converted to VCF format using 'destruct2VCF.py' supplementary tool.

Alignment, chimeric and valid probabilities are set to be higher than 0.6. Calls with tumor read support below 2 are filtered:

```
python destruct2VCF.py -b silico2-result/breakpoints -f silico2-result/filtered-reads -o silico2-result/original.vcf -a 0.6 -c 0.6 -v 0.6 -t 2
```

ICGC-TCGA DREAM Somatic Mutation Calling Challenge
Submission Documentation Form

Submission name	destruct-vcftest1 (ID: 2385670)
Team	SFU
Submission type	☒ SV or ☐ SNV (Only choose one)

1. Read alignment. Did you use the BAM files provided?

☒ Yes ☐ No (Specify your aligner below)

Aligner		Version	
Command lines with parameters			

2. Pre-processing steps to calibrate the BAM files. (Command lines and parameters)

3. Mutation calling algorithm.

Algorithm name	deStruct	Version	0.1.0
Command lines with parameters	python destruct.py –myconfig.ini --tmpdir silico2-temp --submit asyncqsub --maxjobs 100 --repopulate --verbose silico2.lib silico2-result/breakpoints silico2-result/breakreads		

Thresholds not indicated by command line	
Reference (check if unpublished ☒)	

4. Post-VCF filtering steps. (Command lines and parameters)

5. (Optional) Any other comments or steps not covered.

After execution of deStruct, the output file 'breakpoints' is converted to VCF format using 'destruct2VCF.py' supplementary tool.

Alignment, chimeric and valid probabilities are set to be higher than 0.6. Calls with tumor read support below 3 are filtered:

python destruct2VCF.py -b silico2-result/breakpoints -f silico2-result/filtered-reads -o silico2-result/original.vcf -a 0.6 -c 0.6 -v 0.6 -t 3

ICGC-TCGA DREAM Somatic Mutation Calling Challenge
Submission Documentation Form

Submission name	destruct-vcftest1 (ID: 2385691)
Team	SFU
Submission type	☒ SV or ☐ SNV (Only choose one)

1. Read alignment. Did you use the BAM files provided?

☒ Yes ☐ No (Specify your aligner below)

Aligner		Version	
Command lines with parameters			

2. Pre-processing steps to calibrate the BAM files. (Command lines and parameters)

3. Mutation calling algorithm.

Algorithm name	deStruct	Version	0.1.0
Command lines with parameters	python destruct.py –myconfig.ini --tmpdir silico2-temp --submit asyncqsub --maxjobs 100 --repopulate --verbose silico2.lib silico2-result/breakpoints silico2-result/breakreads		

Thresholds not indicated by command line	
Reference (check if unpublished ☒)	

4. Post-VCF filtering steps. (Command lines and parameters)

5. (Optional) Any other comments or steps not covered.

After execution of deStruct, the output file 'breakpoints' is converted to VCF format using 'destruct2VCF.py' supplementary tool.

Alignment, chimeric and valid probabilities are set to be higher than 0.6. Calls with tumor read support below 4 are filtered:

python destruct2VCF.py -b silico2-result/breakpoints -f silico2-result/filtered-reads -o silico2-result/original2.vcf -a 0.6 -c 0.6 -v 0.6 -t 4

ICGC-TCGA DREAM Somatic Mutation Calling Challenge
Submission Documentation Form

Submission name	destruct-vcftest1 (ID: 2385711)
Team	SFU
Submission type	☒ SV or ☐ SNV (Only choose one)

1. Read alignment. Did you use the BAM files provided?

☒ Yes ☐ No (Specify your aligner below)

Aligner		Version	
Command lines with parameters			

2. Pre-processing steps to calibrate the BAM files. (Command lines and parameters)

3. Mutation calling algorithm.

Algorithm name	deStruct	Version	0.1.0
Command lines with parameters	python destruct.py –myconfig.ini --tmpdir silico2-temp --submit asyncqsub --maxjobs 100 --repopulate --verbose silico2.lib silico2-result/breakpoints silico2-result/breakreads		

Thresholds not indicated by command line	
Reference (check if unpublished ☒)	

4. Post-VCF filtering steps. (Command lines and parameters)

5. (Optional) Any other comments or steps not covered.

After execution of deStruct, the output file 'breakpoints' is converted to VCF format using 'destruct2VCF.py' supplementary tool.

Alignment, chimeric and valid probabilities are set to be higher than 0.6. Calls with tumor read support below 5 are filtered:

python destruct2VCF.py -b silico2-result/breakpoints -f silico2-result/filtered-reads -o silico2-result/original3.vcf -a 0.6 -c 0.6 -v 0.6 -t 5

ICGC-TCGA DREAM Somatic Mutation Calling Challenge
Submission Documentation Form

Submission name	deStruct_1
Team	SFU
Submission type	☒ SV or ☐ SNV (Only choose one)

1. Read alignment. Did you use the BAM files provided?

☒ Yes ☐ No (Specify your aligner below)

Aligner		Version	
Command lines with parameters			

2. Pre-processing steps to calibrate the BAM files. (Command lines and parameters)

3. Mutation calling algorithm.

Algorithm name	deStruct	Version	0.1.1 (mrsFAST integrated)
Command lines with parameters	python destruct.py –myconfig.ini --tmpdir silico3-temp --submit asyncqsub --maxjobs 100 --repopulate --verbose silico3.lib silico3-result/breakpoints silico3-result/breakreads		

Thresholds not indicated by command line	
Reference (check if unpublished ☒)	

4. Post-VCF filtering steps. (Command lines and parameters)

5. (Optional) Any other comments or steps not covered.

After execution of deStruct, the output file 'breakpoints' is converted to VCF format using 'destruct2VCF.py' supplementary tool.

Alignment, chimeric and valid probabilities are set to be higher than 0.95. Calls with tumor read support below 4 are filtered:

python destruct2VCF.py -b silico3-result/breakpoints -f silico3-result/filtered-reads -o silico3-result/out16-p0.95-t4-sub1.vcf -a 0.95 -c 0.95 -v 0.95 -t 4

Note that in this test version of deStruct, re-alignment of reads are performed with mrsFAST-Ultra as an alternative to bowtie2.

ICGC-TCGA DREAM Somatic Mutation Calling Challenge
Submission Documentation Form

Submission name	deStruct_2
Team	SFU
Submission type	☒ SV or ☐ SNV (Only choose one)

1. Read alignment. Did you use the BAM files provided?

☒ Yes ☐ No (Specify your aligner below)

Aligner		Version	
Command lines with parameters			

2. Pre-processing steps to calibrate the BAM files. (Command lines and parameters)

3. Mutation calling algorithm.

Algorithm name	deStruct	Version	0.1.1 (mrsFAST integrated)
Command lines with parameters	python destruct.py –myconfig.ini --tmpdir silico3-temp --submit asyncqsub --maxjobs 100 --repopulate --verbose silico3.lib silico3-result/breakpoints silico3-result/breakreads		

Thresholds not indicated by command line	
Reference (check if unpublished ☒)	

4. Post-VCF filtering steps. (Command lines and parameters)

5. (Optional) Any other comments or steps not covered.

After execution of deStruct, the output file 'breakpoints' is converted to VCF format using 'destruct2VCF.py' supplementary tool.

Alignment, chimeric and valid probabilities are set to be higher than 0.6. Calls with tumor read support below 2 are filtered:

python destruct2VCF.py -b silico3-result/breakpoints -f silico3-result/filtered-reads -o silico3-result/out23-p0.6-t2-sub2.vcf -a 0.6 -c 0.6 -v 0.6 -t 2

Note that in this test version of deStruct, re-alignment of reads are performed with mrsFAST-Ultra as an alternative to bowtie2.

ICGC-TCGA DREAM Somatic Mutation Calling Challenge
Submission Documentation Form

Submission name	deStruct_o2
Team	SFU
Submission type	☒ SV or ☐ SNV (Only choose one)

1. Read alignment. Did you use the BAM files provided?

☒ Yes ☐ No (Specify your aligner below)

Aligner		Version	
Command lines with parameters			

2. Pre-processing steps to calibrate the BAM files. (Command lines and parameters)

3. Mutation calling algorithm.

Algorithm name	deStruct	Version	0.1.1
Command lines with parameters	python destruct.py –myconfig.ini --tmpdir silico3-temp --submit asyncqsub --maxjobs 100 --repopulate --verbose silico3.lib silico3-result/breakpoints silico3-result/breakreads		

Thresholds not indicated by command line	
Reference (check if unpublished ☒)	

4. Post-VCF filtering steps. (Command lines and parameters)

5. (Optional) Any other comments or steps not covered.

After execution of deStruct, the output file 'breakpoints' is converted to VCF format using 'destruct2VCF.py' supplementary tool.

Alignment, chimeric and valid probabilities are set to be higher than 0.2:

python destruct2VCF.py -b silico3-result/breakpoints -f silico3-result/filtered-reads -o silico3-result/out3-p0.2-t0-sub2.vcf -a 0.2 -c 0.2 -v 0.2 -t 0

ICGC-TCGA DREAM Somatic Mutation Calling Challenge
Submission Documentation Form

Submission name	deStruct_o3
Team	SFU
Submission type	☒ SV or ☐ SNV (Only choose one)

1. Read alignment. Did you use the BAM files provided?

☒ Yes ☐ No (Specify your aligner below)

Aligner		Version	
Command lines with parameters			

2. Pre-processing steps to calibrate the BAM files. (Command lines and parameters)

3. Mutation calling algorithm.

Algorithm name	deStruct	Version	0.1.1
Command lines with parameters	python destruct.py –myconfig.ini --tmpdir silico3-temp --submit asyncqsub --maxjobs 100 --repopulate --verbose silico3.lib silico3-result/breakpoints silico3-result/breakreads		

Thresholds not indicated by command line	
Reference (check if unpublished ☒)	

4. Post-VCF filtering steps. (Command lines and parameters)

5. (Optional) Any other comments or steps not covered.

After execution of deStruct, the output file 'breakpoints' is converted to VCF format using 'destruct2VCF.py' supplementary tool.

Alignment, chimeric and valid probabilities are set to be higher than 0.1:

python destruct2VCF.py -b silico3-result/breakpoints -f silico3-result/filtered-reads -o silico3-result/merge-1and3-sub3.vcf -a 0.1 -c 0.1 -v 0.1 -t 0

ICGC-TCGA DREAM Somatic Mutation Calling Challenge
Submission Documentation Form

Submission name	deStruct-org
Team	SFU
Submission type	☒ SV or ☐ SNV (Only choose one)

1. Read alignment. Did you use the BAM files provided?

☒ Yes ☐ No (Specify your aligner below)

Aligner		Version	
Command lines with parameters			

2. Pre-processing steps to calibrate the BAM files. (Command lines and parameters)

3. Mutation calling algorithm.

Algorithm name	deStruct	Version	0.1.1
Command lines with parameters	python destruct.py –myconfig.ini --tmpdir silico3-temp --submit asyncqsub --maxjobs 100 --repopulate --verbose silico3.lib silico3-result/breakpoints silico3-result/breakreads		

Thresholds not indicated by command line	
Reference (check if unpublished ☒)	

4. Post-VCF filtering steps. (Command lines and parameters)

5. (Optional) Any other comments or steps not covered.

After execution of deStruct, the output file 'breakpoints' is converted to VCF format using 'destruct2VCF.py' supplementary tool.

Alignment, chimeric and valid probabilities are set to be higher than 0.6. Calls with tumor read support below 2 are filtered:

python destruct2VCF.py -b silico3-result/breakpoints -f silico3-result/filtered-reads -o silico3-result/out1-p0.6-t2-sub1.vcf -a 0.6 -c 0.6 -v 0.6 -t 2
